# Decoupled phenotypic constraints framed by respiratory adaptation in the rise of land vertebrates

**DOI:** 10.1126/sciadv.aeb0801

**Published:** 2026-04-01

**Authors:** Yilun Yu, Xing Xu, Roger B. J. Benson

**Affiliations:** ^1^ American Museum of Natural History, New York NY, USA.; ^2^ Key Laboratory of Vertebrate Evolution and Human Origins, Institute of Vertebrate Paleontology and Paleoanthropology, Chinese Academy of Sciences, Beijing, China.; ^3^ Centre for Vertebrate Evolutionary Biology, Yunnan University, Kunming, China.

## Abstract

Land vertebrates today comprise amniotes and lissamphibians, which have highly different modes of gas exchange, and distinct skull shapes and body size distributions. A central hypothesis of tetrapod evolution proposes a connection between these traits during their initial divergence, 335 million years ago. However, this has yet to be tested in a broad phylogenetic context. We investigate the evolution of body size, skull proportions, and respiratory traits among early land vertebrates, from the Middle Devonian to Early Permian using quantitative analysis of a dataset incorporating 344 species. We find that lissamphibian precursors show stronger constraints on body size than stem amniotes and increases in relative skull height were facilitated by relaxed constraints on the amniote stem lineage. These differences can be explained by respiratory innovations. Dependence on cutaneous gas exchange constrained lissamphibians and their close relatives to small body sizes, whereas rib-based lung ventilation relaxed constraints on skull shape and maximum body size in terrestrial amniotes.

## Teaser

Macroevolutionary models show that rib-based breathing released constraints on body size and cranial evolution in early amniotes.

## INTRODUCTION

The water-to-land transition is one of the most iconic events in vertebrate evolution (*1*) and gave rise to the two major groups of living tetrapods with highly divergent traits: Amniota and Lissamphibia (*2*, *3*). Amniotes have disparate body sizes (0.2 to 180,000,000 g among living species) and have been the dominant component of terrestrial ecosystem since the Early Permian, about 299 million years ago (Ma) (*4*), shortly after their first appearance in late Carboniferous. Lissamphibians are also diverse today but are limited to small body sizes (0.03 to 10,800 g). This limitation may result from constraints imposed by their respiratory system that is characterized by cutaneous gas exchange and buccal pumping (i.e., using the mouth cavity) for lung ventilation (*5*–*7*). These respiratory modes are efficient in water but are less efficient in air due to slower excretion of waste CO_2_ (*8*–*11*), favoring small body sizes, which maximize the ratio between surface area and volume for cutaneous gas exchange. In contrast, amniotes ventilate their lungs using rib motions (costal lung ventilation), which allows high-volume lung ventilation and thereby efficient CO_2_ excretion on land, allowing them to overcome evolutionary constraints on upper body size in terrestrial vertebrates (*9*, *10*). Therefore, important hypotheses of vertebrate terrestrialization propose a plausible connection between body size evolution and the advent of costal lung ventilation during the deep divergence between amniote and lissamphibian antecedents (*9*, *10*). However, these have not yet been evaluated in a broad phylogenetic context.

Previous quantitative studies of body size evolution among early tetrapods have focused on subgroups only and discussed their relationship with hypothesized respiratory strategies (*9*, *10*, *12*–*14*). These works have suggested that large-bodied stem tetrapods and temnospondyls mainly excreted CO_2_ using internal gills, in water (*10*, *15*, *16*), with the assistance of buccal lung ventilation and skin breathing (*10*). The functional requirements of buccal pumping are hypothesized as constraining these taxa to have short necks and low, broad head shapes (*9*, *10*). Gills were then lost on the lissamphibian stem lineage, in Dissorophoidea, in which the skin became more permeable, indicating a strengthening of cutaneous gas exchange (*10*, *15*, *17*). In contrast, osteological correlates of costal lung ventilation, including mesiodistally curved trunk ribs, a neck elongate, and steeper skull, evolved in stem amniotes. Costal lung ventilation allows more efficient CO_2_ excretion in air and is hypothesized to have released constraints on body size, head shape, and neck length, facilitating the evolutionary origin of herbivory and other ecological traits (*9*). However, most studies of the impact of these respiratory adaptations have used qualitative approaches, were limited to study of either amniotes or non-amniotes (*8*–*10*), or used small datasets or nonphylogenetic methods that do not evaluate evolutionary dynamics (*12*–*14*, *18*). Thus, the effects of respiratory innovation on the evolution of body size, skull proportions, and other traits are poorly understood, and long-standing hypotheses remain incompletely tested.

We evaluate the evolution of body size, cranial proportions, neck length, and respiratory traits using a notably expanded dataset of 344 fossil species, focusing on early-diverging tetrapods from the Middle Devonian to the Early Permian, encompassing the Carboniferous origin of the amniote crown group. We quantify variation in phylogenetic optima and constraints on both the body size and the relative skull height among groups and interpret these in light of reconstructions of the evolutionary divergences of traits associated with respiratory adaptation, including rib morphology and length of the cervical region, using evolutionary model fitting, integrated with sensitivity analyses on phylogenies with alternative topologies and branch durations (*19*, *20*).

## RESULT

**Fig. 1.**
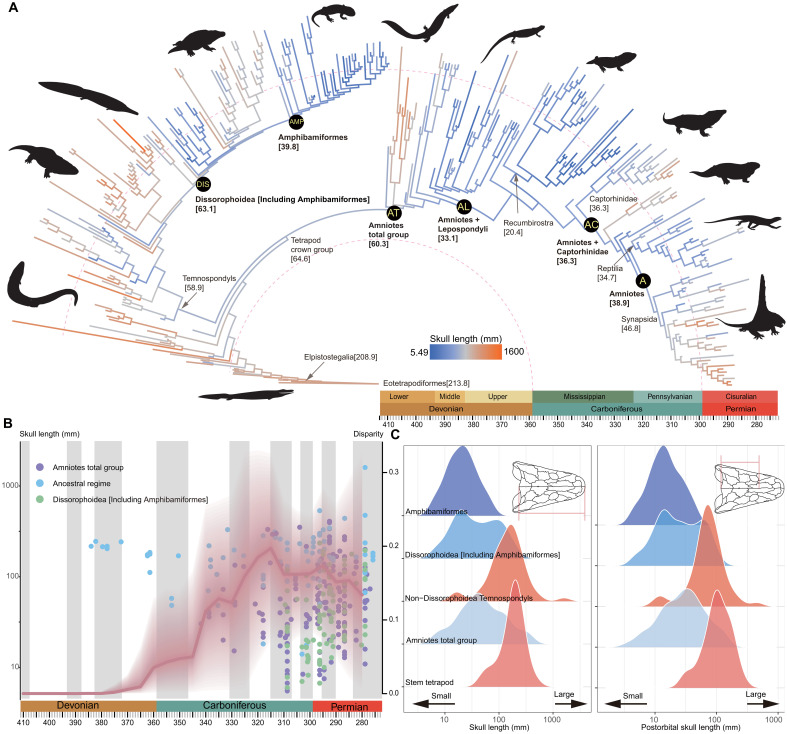
Patterns of body size evolution and distribution among early-diverging tetrapods. (**A**) Ancestral state reconstruction of body size (log_10_ SL) with the calibrated majority rule consensus tree constrained as Topology 1. Yellow abbreviations with black background represent regimes used in subsequent evolutionary model fitting. (**B**) Body size disparity (red line) estimated from the phylogenetic gradual split model using all dated trees constrained as Topology 1, with the body size plotted through time (log_10_ SL). The red dashed area represents 95% confidence interval. (**C**) Body size distributions (both log_10_ SL and OSL) among different groups of early-diverging tetrapods. The analyses and original plots were performed with log_10_-transformed data, whereas the legends on trees and axis are back-transformed to the original scale for ease of reader interpretation. Silhouettes are from http://phylopic.org/. We thank Obsidian Soul, T. M. Keesey, D. Bogdanov, Jagged Fang Designs, B. Otoo, Smokeybjb, R. Díaz Sibaja, Nix Illustration, and Z. Lewis for creating silhouettes in http://phylopic.org/. The silhouettes by T.M. Keesey, D. Bogdanov, Smokeybjb, and Obsidian Soul are under a CC BY-SA 3.0 license (https://creativecommons.org/licenses/by-sa/3.0/deed.en). No changes have been made to these silhouettes. The *Pantylus cordatus* silhouette by D. Bogdanov is under a CC BY 4.0 license (https://creativecommons.org/licenses/by/4.0/deed.en). The original drawing by D. Bogdanov, vectorized by R. Díaz Sibaja, is under a CC BY 3.0 license (https://creativecommons.org/licenses/by/3.0/deed.en). The silhouette by Nix Illustration is under a CC BY-NC 3.0 license (https://creativecommons.org/licenses/by-nc/3.0/deed.en).

We use log_10_-transformed midline skull length (SL) and postorbital skull length (OSL) as proxies of body size (*12*, *21*) (Fig. 1 and figs. S1 to S5). Body size disparity remained constant and low through the first 40 million years (Myr) of our study interval (i.e., between 410 and 370 Ma). This was followed by a consistent increase for ~60 Myr, associated with the divergence of the amniote and lissamphibian stem lineages and uninterrupted by the end-Devonian mass extinction. Disparity then remained high from the late Carboniferous to Early Permian (Fig. 1B and figs. S6 to S11).

Ancestral state reconstructions show that tetrapod originated at large body sizes, as seen in most Devonian stem tetrapods, but decreased on the direct stem lineage leading to crown tetrapods (Fig. 1A and figs. S1 to S5). Body size then increased in most temnospondyl lineages, except for Dissorophoidea, in which multiple lineages exhibit even smaller body sizes from the Pennsylvanian onward. Especially small sizes are present in Amphibamiformes, which are widely considered to include lissamphibians (*22*, *23*). Body size remained small along the amniote stem lineage, with a slight increase near the origin of crown amniotes. Within the amniote crown group, most stem reptiles exhibited small sizes by the late Carboniferous, similar to Amphibamiformes and well below the minimum body sizes of other groups. Stem mammals (early Synapsida) showed a size increase in multiple groups of both herbivores and carnivores (Fig. 1A and figs. S1 to S5) (*12*, *24*).

These results indicate that early tetrapod subclades occupied somewhat distinct body size distributions. We evaluated the dynamics of body size evolution underlying these patterns by fitting Ornstein-Uhlenbeck [OU; i.e., constrained evolution (*25*, *26*)] and Brownian motion [BM; i.e., unconstrained evolution (*27*)] models of trait macroevolution. These included both uniform and three-regime models, specifying two regime shifts from an ancestral tetrapod regime: (i) on the lissamphibian stem lineage, either at the node of Dissorophoidea or Amphibamiformes, and (ii) at different points on the amniote stem lineage (Fig. 1A and figs. S1 to S5). Different possible regime-shift locations were evaluated by comparing models using the Akaike Information Criterion with finite sample size correction (AICc) weight.

**Fig. 2.**
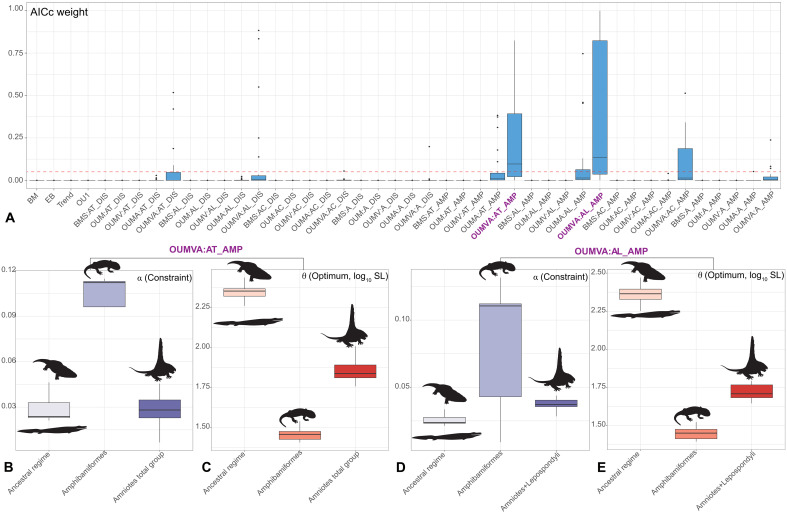
Results of evolutionary model fitting for body size (using log_10_ SL and dated trees constrained as Topology 1). (**A**) AICc weight of uniform and three-regime models. The red dashed line occurred at 0.05. Nonnegligible models are marked in purple. Constraint (α) (**B** and **D**) and optimum (θ) (**C** and **E**) estimated from nonnegligible models. For model and regime names, see Materials and Methods and Fig. 1A. Silhouettes are from http://phylopic.org/. We thank Obsidian Soul, T.M. Keesey, D. Bogdanov, and B. Otoo for creating silhouettes and uploading them to http://phylopic.org/. The silhouettes by T.M. Keesey, D. Bogdanov, Smokeybjb, and Obsidian Soul are under a CC BY-SA 3.0 license (https://creativecommons.org/licenses/by-sa/3.0/deed.en). No changes have been made to these silhouettes. The Pantylus cordatus silhouette by D. Bogdanov is under a CC BY 4.0 license (https://creativecommons.org/licenses/by/4.0/deed.en). The original drawing by D. Bogdanov, vectorized by R. Díaz Sibaja, is under a CC BY 3.0 license (https://creativecommons.org/licenses/by/3.0/deed.en).

The best models, based on the AICc weight, all suggest that the ancestral tetrapod regime has a larger optimal body size (θ) than other regimes. Dissorophoidea or Amphibamiformes always has the highest constraint (or attraction; α) parameter among all three regimes (Fig. 2 and figs. S12 to S23). Models in which both the strength of constraint and the stochastic rate of evolution (σ*^2^*) (OUMVA models) vary among groups greatly outperform others. The best-supported positions for regime shifts occur at Amphibamiformes on the lissamphibian stem lineage and either at the base of the amniote total group or at an amniotes+lepospondyls clade on the amniote stem lineage, for Topologies 1, 2, and 3 (Fig. 2A and figs. S12, S14, S16, S18, S20, and S22). We also found nonnegligible support for the stem lissamphibian regime shift occurring at the Dissorophoidea node, for analyses of log_10_ OSL on Topologies 1 and 2 (figs. S14 and S18). All nonnegligible models support a strong constraint in Amphibamiformes (median α = 0.11) and low constraint in the ancestral regime (median α = 0.024) and the amniote-line regime (median α = 0.028 and 0.037) (Fig. 2, B and D, and figs. S15, S17, S19, S21, and S23). These equate to a phylogenetic half-life (*t*_0.5_) of 6.3 Myr in Amphibamiformes, 28.88 Myr in the ancestral regime, and 24.76 or 18.73 Myr in the amniote-line regime, indicating the timescale on which evolutionary constraints (α) become more influential than a diffusive, Brownian process on body size disparity. Models also return distinct macroevolutionary optima (θ) for the each of the three regimes, with each of them falling within the range of observed size measurements of the included species. The ancestral regime has a large size optimum (median θ log_10_ SL = 2.35 or 2.36), whereas Amphibamiformes have the smallest size of optimum (median θ log_10_ SL = 1.46 or 1.45). The amniote-line regime has a body size optimum intermediate between those of Amphibamiformes and the ancestral regime (median θ log_10_ SL = 1.84 or 1.71) (Fig. 2, C and E, and figs. S15, S17, S19, S21, and S23).

**Fig. 3.**
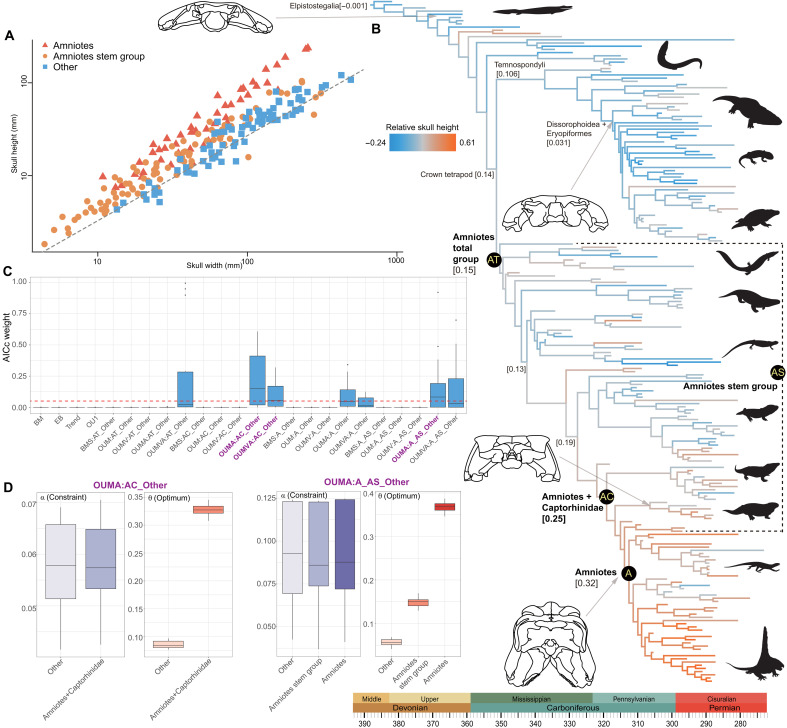
Evolution of relative skull height across early-diverging tetrapods (based on Topology 1). (**A**) Phylogenetic linear regression between the log_10_ skull height and the log_10_ skull width. (**B**) Ancestral state reconstruction of relative skull height with the calibrated majority rule consensus tree. Yellow abbreviations with black background represent regimes used in subsequent evolutionary model fitting. (**C**) AICc weight of uniform and two- and three-regime models, using regimes defined by lineages. The red dashed line occurred at 0.05. Nonnegligible models are marked in purple. (**D**) Constraint (α) and optimum (θ) estimated from nonnegligible models. For model and regime names, see Materials and Methods and (B). The analyses and original plots were performed with log_10_-transformed data, whereas the legends on the axis in (A) are back-transformed to the original scale for ease of reader interpretation. Silhouettes are from http://phylopic.org/. We thank Obsidian Soul, T. M. Keesey, D. Bogdanov, Jagged Fang Designs, B. Otoo, Smokeybjb, R. Díaz Sibaja, Nix Illustration, Z. Lewis, and W. Toosey for creating silhouettes in http://phylopic.org/. The silhouettes by T.M. Keesey, D. Bogdanov, Smokeybjb, and Obsidian Soul are under a CC BY-SA 3.0 license (https://creativecommons.org/licenses/by-sa/3.0/deed.en). No changes have been made to these silhouettes. The Pantylus cordatus silhouette by D. Bogdanov is under a CC BY 4.0 license (https://creativecommons.org/licenses/by/4.0/deed.en). The silhouette by W. Toosey is also under a CC BY 4.0 license. The original drawing by D. Bogdanov, vectorized by R. Díaz Sibaja, is under a CC BY 3.0 license (https://creativecommons.org/licenses/by/3.0/deed.en ). The silhouette by Nix Illustration is under a CC BY-NC 3.0 license (https://creativecommons.org/licenses/by-nc/3.0/deed.en).

An abrupt increase in relative skull height occurred close to the origin of amniotes. We show this using the residuals from phylogenetic regressions of log_10_ skull height on log_10_ skull width, as an index of relative skull height (Fig. 3A and fig. S24). Ancestral state reconstructions indicate that the relative skull height remained low in most stem tetrapods and temnospondyls, with the exception of Whatcheeriidae, which evolved proportionally high skulls independently of amniotes, potentially as an adaption for more efficient vertical biting (*28*, *29*). The ancestral broad, flat skull was also present in most temnospondyls (Fig. 3B and figs. S25 and S26).

Evolutionary increases in relative skull height occurred on the line leading to amniotes, and most stem amniotes have quantitatively intermediate skull proportions (Fig. 3B and figs. S25 and S26). Two-regime model with constant stochastic rate but varying constraints among groups (OUMA), with regime shifts at an amniotes+Captorhinidae clade, are most favored in analyses with Topology 1 (Fig. 3C). A two-regime OUMVA model, with regime shifts at an amniotes+Captorhinidae clade, and a three-regime OUMA model, with regime shifts at the base of amniotes and the origin of crown amniotes, also receive nonnegligible support (Fig. 3C and fig. S27). When analyses are conducted with Topology 2, a two-regime OUMVA model specifying a regime shift at the base of amniote-line tetrapod receives the strongest support, and two-regime OUMVA and OUMA models specifying a regime shift at an amniotes+Captorhinidae clade receive nonnegligible support (fig. S29). A two-regime OUMA model, with regime shifts at the origin of crown amniotes, is the best model in analyses with Topology 3 (fig. S31). In three-regime models, crown amniotes have the largest optimum (median θ = 0.37), whereas the ancestral regime has the smallest optimum (median θ = 0.056) and stem amniote have an intermediate optimum (median θ = 0.15) (Fig. 3D). In two regime models, crown amniotes or inclusive clades subtending to crown amniotes exhibits a larger optimum (median θ = 0.33 or 0.34) than that in the ancestral regime (median θ = 0.084 or 0.086) (Fig. 3D and figs. S28, S30, and S32). Most of the two- and three-regime models return almost the same constraints in different regimes (median α*_amniotes+Captorhinidae_* = 0.057 or 0.063, *t*_0.5_ = 12.16 or 11.00 Myr; median α*_ancestral regime_* = 0.058 or 0.068, *t*_0.5_ = 11.95 or 10.19 Myr; median α*_amniotes_* = 0.088, with *t*_0.5_ = 7.88 Myr; median α*_amniote stem group_* = 0.086, with *t*_0.5_ = 8.06 Myr; median α*_ancestral regime_* = 0.093, with *t*_0.5_ = 7.45 Myr) (Fig. 3D and figs. S28, S30, and S32). Notably, when analyses are conducted with Topology 2 using the OUMVA model, the amniotes+Captorhinidae or amniotes total group exhibits a median optimum exceeding the range of observed values and constraint lower than that in the ancestral regime, suggesting a directional trend-like increase in skull height within early crown amniotes and their closest relatives (fig. S30) (*25*, *30*, *31*).

**Fig. 4.**
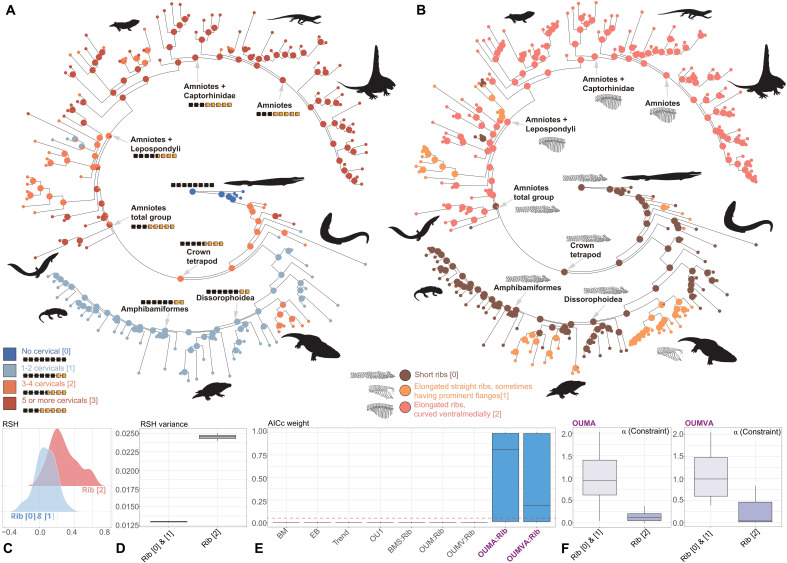
Evolution of cervical count and rib morphology across early-diverging tetrapods and the release of skull shape resulted from evolution of costal lung ventilation (based on Topology 1). (**A**) Primary ancestral state reconstruction of cervical count using the SYM model. (**B**) Ancestral state reconstruction of rib morphology using the SYM model. (**C**) Density plots show the distribution of relative skull height of regimes defined by rib morphology. (**D**) Boxplot visualizes the difference of variance across the regimes defined by rib morphology, summarizing from the relative skull height estimated using all dated trees. (**E**) AICc weight of uniform and two-regime models, using regimes defined by rib morphology. (**F**) Constraint (α) estimated from nonnegligible models (OUMA and OUMVA). Silhouettes are from http://phylopic.org/. We thank Obsidian Soul, T. M. Keesey, D. Bogdanov, B. Otoo, Nix Illustration, and Z. Lewis for creating silhouettes and uploading them to http://phylopic.org/. The silhouettes by T.M. Keesey, D. Bogdanov, Smokeybjb, and Obsidian Soul are under a CC BY-SA 3.0 license (https://creativecommons.org/licenses/by-sa/3.0/deed.en). No changes have been made to these silhouettes. The Pantylus cordatus silhouette by D. Bogdanov is under a CC BY 4.0 license (https://creativecommons.org/licenses/by/4.0/deed.en). The silhouette by W. Toosey is also under a CC BY 4.0 license. The original drawing by D. Bogdanov, vectorized by R. Díaz Sibaja, is under a CC BY 3.0 license (https://creativecommons.org/licenses/by/3.0/deed.en). The silhouette by Nix Illustration is under a CC BY-NC 3.0 license (https://creativecommons.org/licenses/by-nc/3.0/deed.en).

**Fig. 5.**
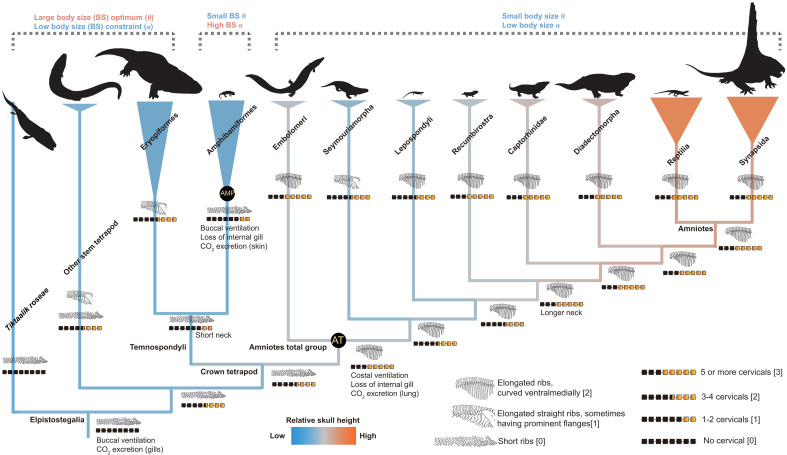
The summarizing figure illustrates the major macroevolutionary patterns observed in body size, skull proportion, and respiratory traits. The pattern of trait evolution is mapped on a simplified phylogeny based on results from analyses with Topology 1. Evolution of rib morphology and cervical count are mapped according to results from the SYM model (see Fig. 4). Branches are colored according to relative skull height. Yellow abbreviations with black background represent regimes used in the nonnegligible models describing body size evolution. Silhouettes are from http://phylopic.org/. We thank Obsidian Soul, T. M. Keesey, D. Bogdanov, Jagged Fang Designs, B. Otoo, Smokeybjb, R. Díaz Sibaja, Nix Illustration, Z. Lewis, and W. Toosey for creating silhouettes in http://phylopic.org/. The silhouettes by T.M. Keesey, D. Bogdanov, Smokeybjb, and Obsidian Soul are under a CC BY-SA 3.0 license (https://creativecommons.org/licenses/by-sa/3.0/deed.en). No changes have been made to these silhouettes. The Pantylus cordatus silhouette by D. Bogdanov is under a CC BY 4.0 license (https://creativecommons.org/licenses/by/4.0/deed.en). The silhouette by W. Toosey is also under a CC BY 4.0 license. The original drawing by D. Bogdanov, vectorized by R. Díaz Sibaja, is under a CC BY 3.0 license (https://creativecommons.org/licenses/by/3.0/deed.en). The silhouette by Nix Illustration is under a CC BY-NC 3.0 license (https://creativecommons.org/licenses/by-nc/3.0/deed.en).

Long, mesiodistally curved ribs and longer cervical region evolved in amniote-line tetrapods, whereas short ribs and a short cervical region evolved in Dissorophoidea (Figs. 4 and 5). Ancestral state reconstructions of discrete characters describing variation in cervical count (neck length) and rib morphology indicate similar support for three different models of transition frequencies. A symmetric rate (SYM) model performs marginally best in our primary analyses of cervical count and rib morphology (figs. S33 and S53), and all-rates-different (ARD) model performs marginally best on our additional analyses of cervical count evolution with all temnospondyls are coded as having only two cervical vertebrae: the atlas and the axis (fig. S43).

Early elpistostegalians had short ribs and a short neck lacking distinct cervical vertebrae (*32*, *33*). Cervical count increased in the earliest tetrapods, and an even longer cervical region was present in crown amniotes (*34*). In contrast, shorter cervical regions evolved along the lissamphibian stem lineage (Fig. 4A and figs. S34 to S52). Elongated ribs having prominent flanges or uncinate processes evolved in more derived stem tetrapods, such as *Ichthyostega* and *Whatcheeria*, but the inferred conditions of the common ancestors of crown tetrapods and total group amniotes are short ribs. Mesiodistally curved, elongate ribs evolved early on the amniotes stem lineage and were inherited by crown amniotes. Among temnospondyls, eryopiforms and olsoniforms evolved elongate ribs, whereas amphibamiforms retained shortened straight ribs that were inherited by lissamphibians (Fig. 4B and figs. S54 to S62).

Our density plot show that taxa with mesiodistally curved elongate ribs suitable for costal lung ventilation have not only larger relative skull heights but also larger variation, shown by the variance of residuals from skull height versus skull width (Fig. 4, C and D, and fig. S63). OUMA and OUMVA models, in which groups with long, curving ribs have distinct dynamics of relative skull height evolution, greatly outperform uniform models (Fig. 4E and fig. S64). All nonnegligible models support a distinctly stronger constraint in taxa bearing ribs not suitable for costal ventilation (median α = 0.94 or 0.99, *t*_0.5_ = 0.74 or 0.7) and much lower constraint in taxa with long, curving ribs (median α = 0.11 or 0.032, *t*_0.5_ = 6.3 or 21.7) (Fig. 4F and fig. S65). Meanwhile, the estimation of optimum in regime of mesiodistally curved elongate ribs is unstable compared to that in another regime, showing great variation in analyses with trees in different topology and branch durations. This instability is likely caused by the high variability of residual skull height within taxa developing mesiodistally curved elongate ribs (fig. S65).

## DISCUSSION

Our analyses strongly support that different lineages of early tetrapods exhibited distinct modes of body size evolution. Antecedents of both amniotes and lissamphibians shifted independently into small-sized adaptive zones, from a large-bodied common ancestor (Fig. 2, C and E, and figs. S15, S17, S19, S21, and S23). This pattern strongly contradicts the hypothesis of general tendency toward increasing of body size during early tetrapod evolution (*35*), as is evident in the negligible statistical support of a directional trend model (Fig. 2A and figs. S12, S14, S16, S18, S20, and S22). Among amniotes, some synapsids and herbivorous stem reptile lineages show evolutionary increases in body size (Fig. 1A and figs. S1 to S5). However, body size increases are not universal among early amniote lineages and, in our models, can be explained by evolutionary expansion of upper body sizes under low constraint (low α), rather than directional, trend-like evolution (*30*, *31*) (Fig. 2, B and D, and figs. S15, S17, S19, S21, and S23). We also found no evidence for an important impact of the end-Devonian mass extinction on tetrapod body size evolution. An uninterrupted increase in disparity through the Devonian-Carboniferous boundary indicates that tetrapods recovered quickly in the Tournaisian, shortly after the end-Devonian mass extinction (Fig. 1B and figs. S6 to S11) and as confirmed by recently found tetrapod faunas of the early Carboniferous (*36*, *37*). We therefore reject the hypothesis of a prolonged Lilliput effect in post-Devonian tetrapod evolution suggested by a previous study (*13*) that used a nonphylogenetic methodology.

Our data confirm that buccal lung ventilation was the plesiomorphic respiratory mode of all land vertebrates and inherited by the precursors of lissamphibians (*10*) (Fig. 5). Most stem tetrapods and temnospondyls had short, straight ribs, with several lineages independently evolving elongate ribs (Fig. 4B and figs. S54 to S62). However, these elongated ribs are straight or only slightly curved, exhibiting prominent, overlapping flanges or uncinate process that suggest a function of postural support rather than lung ventilation (*9*). These traits also co-occur with proportionally flat skulls (Figs. 4 and 5), a short cervical region (Fig. 5 and figs. S34 to S52), both of which are expected under the hypothesis that the absence of costal ventilation places constraints on a short neck and head shape to a wide and shallow morphology (*9*). Traits associated with costal lung ventilation exhibit a mosaic evolutionary pattern on the amniote stem lineage. Mesiodistally curved ribs evolved early in stem amniotes (Figs. 4B and 5), suggesting that these precursors of modern reptiles and mammals used costal lung ventilation (*9*), probably related to unilateral rib motion during locomotion (*38*).

Amphibamiform stem lissamphibians exhibit stronger constraints on body size evolution than either ancestral tetrapods or amniotes and their stem linage (Figs. 2 and 5). Our findings are consistent with the hypothesis that these differences reflect an underlying divergence of respiratory adaptations. Stem tetrapods and most temnospondyls had large body sizes, with unfavorable skin surface-volume ratios. They were unlikely to excrete CO_2_ completely relying on cutaneous respiration and inefficient buccal lung ventilation (*9*–*11*). Given that osteological correlates of internal gills have been reported in a variety of stem tetrapods and temnospondyls, CO_2_ is likely to have mainly excreted via gills in these groups (*10*, *15*, *16*), which may explain how they were able to obtain large body sizes, particularly in aquatic settings. Pronounced dermal sculpture may also be helpful in buffering excess blood CO_2_ (*11*). Cutaneous respiration might have been an auxiliary mode of CO_2_ excretion in stem tetrapods and temnospondyls (*10*) and is widely present in extant tetrapods (*39*). This is especially relevant given the environmental context of early tetrapod evolution. Increasing atmospheric O_2_ and decreasing CO_2_ content started from Late Devonian and continued across Carboniferous and Early Permian (*40*–*42*), which would have facilitated O_2_ uptake and CO_2_ excretion (*9*). Large-sized species have an unfavorable skin surface-volume ratio at which cutaneous respiration is a viable means of CO_2_ excretion in water. Meanwhile, small-sized species have a higher skin surface-volume ratio and greater advantage of cutaneous CO_2_ excretion in both aquatic and terrestrial settings. This environmental context may also have enabled lineages leading to both Lissamphibia and Amniota to shift out of the ancestral size-related adaptive zone and develop different solutions to the common problem of respiratory acidosis. Loss of internal gills in adults occurred in terrestrially adapted Dissorophoidea (including Amphibamiformes) (*10*, *17*), resulting in enhanced reliance on cutaneous CO_2_ excretion. This is consistent with the occurrence of strong constraints toward small body size in this group and inherited by lissamphibians (*10*, *43*).

We find strong evidence that the evolution of costal lung ventilation on the amniote stem lineage led to relaxation of body size constraints and released skull proportions from functional constraints imposed by buccal lung ventilation (Fig. 5). Our findings suggests that lineages with rib morphology suitable for costal lung ventilation show much lower constraints on relative skull height (Fig. 4, C to F), enabling the evolutionary expansion of the upper limit of skull height, especially among crown amniotes (Figs. 3 and 5) (*9*). It is possible that costal lung ventilation in stem amniotes was not as efficient as that of crown amniotes, evident in the proportionally lower skull heights and shorter cervical regions of these animals and supported by our analyses (Figs. 3D and 5). This implies that at least some stem amniotes still relied to some extent on cutaneous respiration for gas exchange (*9*, *10*). The evolution of deeper skulls in stem and especially crown amniotes enabled functional partitioning of adductor muscles, resulting in enhanced ability to exert static pressure during tooth occlusion, an important prerequisite for the evolution of herbivory (*9*, *44*, *45*). This innovation allowed amniotes to radiate into diverse previously unexplored niches by digesting plant resources (*46*), with multiple lineages of both herbivores and their predators overcoming constraints on maximum body size during the Early Permian (*18*, *24*).

The changes that we document here, during the first ~130 Myr of tetrapod evolution, foreshadow the distributions of body size and respiratory adaptations of their living members. Today, amniotes occupy even greater disparity of body sizes among terrestrially adapted species, ranging from those multiton gigantic mammalian herbivores to dwarfed lizards weighing only a few grams, facilitated by the high efficiency of costal lung ventilation in CO_2_ excretion and acid-base homeostasis (*9*, *10*). In contrast, lissamphibians are restricted to small sizes as a consequence of their reliance on cutaneous CO_2_ excretion (*10*). Our findings corroborate that these highly different biological traits in living members of both crown groups have diverged shortly after their origination, establishing the foundation of modern terrestrial ecosystem, hundreds of millions of years predating the extensive radiation of extant species.

## MATERIALS AND METHODS

### Supertree assembly and time calibration

We investigate macroevolutionary patterns of body size and traits related to respiratory function of fossil tetrapods from Middle Devonian to Early Permian. Three composite trees (referring as Topology 1, Topology 2, and Topology 3 in the main text) representing alternative hypotheses of early tetrapod evolution were reconstructed manually in Mesquite 3.81 according to published references (*19*, *20*, *22*, *37*, *45*–*59*). Topology 1 uses the latest topology of amniote-line tetrapod published by Jenkins *et al.* (*59*), placing Captorhinidae as the sister group to an amniotes+Diadectomorpha clade and Recumbirostra as the sister group to an amniotes+*Hylonomus lyelli* clade. Adelospondyls and aistopods are recovered as lepospondyls (*20*), and Embolomeri is maintained as a basal branch of the amniote stem group. *Eldeceeon rolfei*, *Termonerpeton makrydactylus*, and *Silvanerpeton miripedes* form a small clade, placing as the sister group of amniotes+Gephyrostegidae (*48*) (Fig. 1 and fig. S1). Topology 2 represents a phylogenetic framework proposed in recent studies (figs. S2 and S3) (*19*, *57*). Recumbirostra is treated as crown amniotes and placed as the sister group of Reptilia (*57*, *60*). Adelospondyls, aistopods, and Embolomeri are recovered as stem tetrapods and together form a monophyletic group, which is placed as the sister group of Colosteidae (*19*). Topology 3 uses a more traditional topology for amniote-line tetrapod, with Captorhinidae placing as the sister taxon to Reptilia (figs. S4 and S5). All final supertrees include 344 taxa, with *Eusthenopteron* used as the outgroup of all remaining taxa.

We calibrated the trees to stratigraphy using the fossilized birth-death (FBD) model in MrBayes 3.2.8 (*61*), maintaining topological constraints while using an empty character matrix, ensuring that the temporal information was inferred solely from species occurrence time (*62*–*65*). Fossil age priors of each species were represented by a uniform distribution between the bounds of uncertainties on their temporal occurrences, using data originally from the Paleobiology database (PBDB) and revised according to recent published studies (*46*, *52*, *59*, *66*) and an open accessible dataset TEMNOS (*67*) (the final date of data collection and revision is 20 October 2025). We revised the age range for *Hapsidopareion lepton* and other taxa from the Hennessey Formation in Oklahoma by adopting the Artinskian timescale instead of the previously used Kungurian timescale (*52*). We fixed the root age as 412.9 Ma according to ref. (*66*). This value was chosen because it was estimated from a Bayesian tip-dating analysis using a partitioned morphological clock and considering the chronological information of tetrapod trackway in ref. (*66*) [in Extended Data fig. 3c in (*66*)]. Considering the lack of meaningful prior information for net diversification, relative extinction, and relative fossilization rates, a uniform prior U (0,1) was assigned to net diversification rate, and a uniform-like Beta prior B (1,1) was assigned to both the relative extinction and the relative fossil-sampling rate. We conducted two independent Markov chain Monte Carlo (MCMC) runs with four chains (one cold and three hot) per run for 200,000,000 iterations and sampled every 40,000 iterations. The first 30% samples were discarded as burn-in for each run.

Our analyses were conducted on 24 trees sampled at random from the posterior distributions of time-calibrated trees for each topology. With the addition of three majority rule consensus trees, for a total of 75 trees. Species with missing data for each analysis were dropped from our trees before analysis.

### Body size proxies and respiratory traits dataset

Extinct early-diverged tetrapods are morphologically diverse and frequently represent by incomplete remains. This presents challenges for estimating body mass across a wide phylogenetic scope. To maximize the number of species, we used midline SL as a body size index. This was measured as the distance between the snout tip and the posterior dorsal midpoint of the skull. Considering that SL is sometimes strongly correlated with ecological factors that cause variation in relative snout length, we also collected the data of OSL, measuring from the anterior margin of the orbit to the posterior dorsal midpoint of the skull (*21*). We primarily sample taxa that have an almost complete skull preserved, with photos, line drawings, or reconstructions present in a peer-reviewed publication. This allows us to easily measure or calculate data using the figures. We also used peer-reviewed restorations of the incompletely preserved skulls of some taxa based on close relatives, and estimated SLs stated in the text, for incomplete skulls in which the preservation is sufficient to allow estimates of other data (e.g., OSL). Last, we compared our data to the TEMNOS database of temnospondyl SL (*67*), rechecking there we found large discrepancies and replacing notably smaller skull measurements with the largest value present in TEMNOS, where the sources could be verified. The final dataset including the SL and OSL of 338 species. All measurements, including those described below, were log_10_ transformed for all analyses (figs. S1 to S5).

We also measured both the skull height and the skull width of 214 early tetrapod species, with photos, line drawing, or reconstructions present in published studies. The skull height was measured as the vertical distance from the articular plane of the jaw joint to the highest point on the skull roof. Skull width was defined as the maximum transverse width in dorsal view (Fig. 3 and figs. S25 and S26).

Last, we assembled data on cervical vertebral count and dorsal rib morphology. We use the transition of rib morphology as the standard of identifying cervicodorsal boundary as this standard is widely used to identify the cervical region in stem tetrapods (*68*, *69*), stem amniotes, and amniotes (*34*, *70*–*72*). Information of change of rib morphology, including the presence or absence of ribs, are observed from the description and illustrated figures in published studies. Cervical counts of most crown amniotes are taken from a published broad-scale study of vertebral number evolution in amniotes (*34*). The final dataset comprised cervical counts for 142 species. Considering the incomplete preservation of cervical series and the vague of cervicodorsal boundary in some species, we then discretized cervical count into four states, including (0) no cervical vertebrae, (1) one to two cervical vertebrae, (2) three to four cervical vertebrae, and (3) five or more cervical vertebrae. Trunk rib morphology is observable in 198 taxa, and we divided them into three discrete states, including (0) extreme short rib, which is shorter than or approximately equal to twice of the transverse width of a centrum; (1) elongated ribs with relatively straight shaft, sometimes bearing extensive flanges or uncinate processes that create broad intercostal overlap; and (2) mesiodistally curved and elongated ribs, with no or weak flanges (Fig. 4 and figs. S34 to S52 and S54 to S62).

All data are in millimeters and collected from studies published on peer-reviewed journals, either from a digital copy or a printed book (*Edops craigi*). For how we collect the data of each species, see the description in data S1 (*73*). The final date of data collection and revision is 20 October 2025.

### Body size macroevolutionary analyses

To visualize patterns of body size evolution, we performed maximum likelihood ancestral state reconstruction on log_10_-transformed body size proxies across all time-calibrated phylogenies, implementing the “fastAnc” function in the R package “phytools” (*74*). We then conducted body size disparity analyses, using variance as the metric of disparity. We used two variants of the phylogenetic time-slicing method: the “equal split” and “gradual split” models (*75*). Both methods not only consider internal nodes and tips but also branch length information, which can inflate the sample through time and avoid disparity gaps for those time periods with poor fossil records. The “equal split” method samples the value from both the ancestor and the descendent with equal probability, whereas the latter samples value using different probabilities, which are defined as the distance of slice and ancestor/descendent divided by branch duration (*75*). The trees were sliced with 5-Myr intervals. The distribution of disparity metric at each time point was simulated with 100 bootstraps, with rarefaction limiting the number of elements drawn from each bootstrap replication. We then plotted disparity through time by summarizing the distribution of disparity metric of each time slice estimated from multiple time trees (Fig. 1B and figs. S6 to S11).

We then performed model fitting to characterize the macroevolutionary dynamics of body size evolution across early tetrapods. Comparison of uniform to multiregime models tests the hypothesis that dynamics of body size evolution varies among groups of early tetrapods and comparison of BM to OU models tests between diffusive or unconstrained dynamics versus constrained dynamics (represented by α for constraint and θ for the optimal trait value in OU). We fitted four uniform models, including BM, early burst (EB), directional trend (Trend), and OU. We also fitted three-regime models, specifying two regime shifts from an ancestral tetrapod regime: (i) on the lissamphibian stem lineage, either at the node of Dissorophoidea or Amphibamiformes, and (ii) at different points on the amniote stem lineage (Fig. 1A and figs. S1 to S5). The fitted models include the multirate Brownian motion (BMS), multioptimum OU model (OUM), multioptimum OU model with regime-specific stochastic spreads (OUMV), multioptimum OU model with regime-specific constraints (OUMA), and OU model with all three regime-specific parameters (OUMVA). Model comparison was performed using the AICc, where lower AICc scores corresponding to better fitting models. AICc weights for each family of analyses (based on specified topology and measurement) were calculated with the function “akaike.weights” implemented in the R package “qpcR” (*76*). The BM, EB, and Trend models were fitted with function “fitContinuous” implemented in the R package “geiger” (*77*), whereas other models were fitted with the function “OUwie” in the R package “OUwie” (*26*). Values from both packages are directly comparable according to our preliminary analyses and published studies, including log likelihood and AICc value (*78*). Considering that potential bias might be introduced by missing the measurements of even larger specimens or from other sources of disturbances, such as error introduced by the scale bar in references, we incorporate a theoretical SE in all our model fitting (also in model fitting described below). We used 0.0217/3 as the measurement error of both log_10_-transformed SL and OSL within species (because body mass follows cubic scaling relative to linear morphometric traits), which has been shown to be a reasonable magnitude of variation in size-related traits in vertebrates (*31*). Distributions of model parameters and AICc values (including AICc weights) were given from analyses of all 25 calibrated trees for each topology (Fig. 2 and figs. S12 to S23). Models with a median AICc weight larger than 0.05 are treated as nonnegligible models (*30*).

### Relative skull height macroevolutionary analyses

We indexed variation in relative skull height using the residuals from a phylogenetic linear regression between log_10_-transformed skull height and skull width for each time-scaled phylogeny, using the function “phylolm” implemented in the R package “phylolm” (*79*, *80*). Pagel’s λ, a phylogenetic signal parameter, was estimated using maximum likelihood (Fig. 3A and fig. S24). We performed ancestral state reconstruction for visualizing the evolutionary pattern of relative skull height residuals (Fig. 3B and figs. S25 and S26). To test the hypothesis that released skull shape from constraints imposed by buccal pumping occurred on amniote-line tetrapods (*9*), we compared macroevolutionary models using the same approaches described above for body size indexes. We compared uniform models to multiregime models specifying a distinct regime in crown amniotes, amniotes+Captorhinidae (only for Topologies 1 and 2), or amniote total group (two-regime models). Then, we tested the differences of evolutionary mode between amniotes, amniote stem groups, and the remaining part of the tree (three-regime models). According to our preliminary analyses, the optimizations of three-regime OUMA and OUMVA models were often failed when analyses are conducted on Topologies 2 and 3. This might be owing to the large number of parameters and the explicit inclusion of branch duration uncertainty and estimation error in our analyses (*30*, *31*). Therefore, we compare both two-regime models and three-regime models for Topology 1, and we only compare different two-regime models for Topologies 2 and 3.

The SE of residuals was calculated as the following function (*81*)

RSE=SE(Log10skull height)×1−R2 (1)

where *R*^2^ is the coefficient of determination of a phylogenetic regression model, and the SE of log_10_ skull height (SE in the formula) was used as the same value of log_10_ SL as both of them are size-related traits. Distributions of parameters and AICc values (including AICc weights) were given from model fitting of all calibrated trees for each topology (Fig. 3 and figs. S27 to S32). Models with a median AICc weight larger than 0.05 are treated as nonnegligible models (*30*).

To further test the hypothesis that the evolution of costal lung ventilation leads to relaxation of constraints in relative skull height, we performed the following analyses with regimes defined by rib morphology (available for 136 species). Species having long, mesiodistally curved trunk ribs (rib code 2) are placed in one regime, and species having other trunk ribs (rib codes 0 and 1) are placed in the second regime. To compare the distribution of relative skull height between the two regimes, we used density plots (Fig. 4C and fig. S63). We also assessed the difference in variance across the two regimes by summarizing the variances calculated from the relative skull height data derived from all dated phylogenies for each topology (Fig. 4D and fig. S63). Then, we tested the differences of evolutionary mode between the two regimes by fitting uniform and two-regime macroevolutionary models. The distributions of parameters and AICc values (including AICc weights) were given from model fitting of all calibrated trees for each topology. Models with a median AICc weight larger than 0.05 are treated as nonnegligible models (Fig. 4, E to F, and figs. S64 and S65).

### Ancestral state reconstruction of discrete characters

To test the hypothesis that the presence of coastal lung ventilation releases constraint on a short cervical region (*9*), we performed ancestral state reconstruction of rib morphology (198 samples) and cervical counts (142 samples) using models for discrete characters. Analyses were conducted with the function “corHMM” implemented in the R package “corHMM” (*82*), with “rate.cat” setting to 1 and “nstarts” setting to 50 according to our preliminary analyses. We used three different models, which use different rate transition matrices, for the analyses of rib morphology, including equal rate (ER), SYM, and ARD models. For cervical vertebral count evolution, we adapted the standard models (ER, SYM, and ARD) to constrain state transitions to adjacent counts only, prohibiting saltational changes, which are shown as follows

$$\text{Modified}\;\text{ER}\;\text{Matrix}\;=\;\left[\begin{array}{cccc} - & a & - & -\\ a & - & a & -\\ - & a & - & a\\ - & - & a & - \end{array}\right]$$ (2)

$$\text{Modified}\;\text{SYM}\;\text{Matrix}\;=\;\left[\begin{array}{cccc} - & a & - & -\\ a & - & b & -\\ - & b & - & c\\ - & - & c & - \end{array}\right]$$ (3)

$$\text{Modified}\;\text{ARD}\;\text{Matrix}\;=\;\left[\begin{array}{cccc} - & a & - & -\\ d & - & b & -\\ - & e & - & c\\ - & - & f & - \end{array}\right]$$ (4)

[in R: modified ER: matrix (c (0, 1, 0, 0, 1, 0, 1, 0, 0, 1, 0, 1, 0, 0, 1, 0), nrow = 4); modified SYM: matrix (c (0, 1, 0, 0, 1, 0, 2, 0, 0, 2, 0, 3, 0, 0, 3, 0), nrow = 4); modified ARD: matrix (c (0, 1, 0, 0, 4, 0, 2, 0, 0, 5, 0, 3, 0, 0, 6, 0), nrow = 4)]. The analyses were performed with all time trees of both topologies, and model performance was compared using the distribution of AICc value. For each node of all time trees, we selected the likeliest state as the ancestral state and calculated the frequency of each state occurred as the ancestral state at every node over the time tree set. We then mapped these summarizing results on the majority rule consensus trees, which are exhibited in our main text figures and supplementary figures (Fig. 4, A and B, and figs. S33 to S42 and S53 to S62).

Given the uncertainties surrounding the cervical counts of temnospondyls (*33*), we performed additional analyses with all available temnospondyls coded as having two cervical vertebrae, the atlas and the axis, using the models described above. The results are reported in figs. S43 to S52.

## References

[1] B. V. Dickson, J. A. Clack, T. R. Smithson, S. E. Pierce, Functional adaptive landscapes predict terrestrial capacity at the origin of limbs. Nature 589, 242–245 (2021). 33239789 10.1038/s41586-020-2974-5

[2] M. I. Coates, M. Ruta, M. Friedman, Ever since Owen: Changing perspectives on the early evolution of tetrapods. Annu. Rev. Ecol. Evol. Syst. 39, 571–592 (2008).

[3] J. A. Clack, *Gaining Ground, Second Edition: The Origin and Evolution of Tetrapods* (Indiana Univ. Press, 2012).

[4] E. M. Dunne, R. A. Close, D. J. Button, N. Brocklehurst, D. D. Cashmore, G. T. Lloyd, R. J. Butler, Diversity change during the rise of tetrapods and the impact of the ‘Carboniferous rainforest collapse’. Proc. Biol. Sci. 285, 20172730 (2018). 29436503 10.1098/rspb.2017.2730 PMC5829207

[5] F. S. Caron, M. R. Pie, The evolution of body size in terrestrial tetrapods. Evol. Biol. 51, 283–294 (2024).

[6] B. T. Kligman, B. M. Gee, A. D. Marsh, S. J. Nesbitt, M. E. Smith, W. G. Parker, M. R. Stocker, Triassic stem caecilian supports dissorophoid origin of living amphibians. Nature 614, 102–107 (2023). 36697827 10.1038/s41586-022-05646-5 PMC9892002

[7] J. S. Anderson, R. R. Reisz, D. Scott, N. B. Fröbisch, S. S. Sumida, A stem batrachian from the Early Permian of Texas and the origin of frogs and salamanders. Nature 453, 515–518 (2008). 18497824 10.1038/nature06865

[8] C. M. Janis, J. G. Napoli, D. E. Warren, Palaeophysiology of pH regulation in tetrapods. Philos. Trans. R Soc. London Ser. B Biol. Sci. 375, 20190131 (2020). 31928199 10.1098/rstb.2019.0131 PMC7017442

[9] C. M. Janis, J. C. Keller, Modes of ventilation in early tetrapods: Costal aspiration as a key feature of amniotes. Acta Palaeontol. Pol. 46, 137–170 (2001).

[10] F. Witzmann, CO_2_-metabolism in early tetrapods revisited: Inferences from osteological correlates of gills, skin and lung ventilation in the fossil record. Lethaia 49, 492–506 (2015).

[11] C. M. Janis, K. Devlin, D. E. Warren, F. Witzmann, Dermal bone in early tetrapods: A palaeophysiological hypothesis of adaptation for terrestrial acidosis. Proc. Biol. Sci. 279, 3035–3040 (2012). 22535781 10.1098/rspb.2012.0558 PMC3385491

[12] M. Laurin, The evolution of body size, Cope’s rule and the origin of amniotes. Syst. Biol. 53, 594–622 (2004). 15371249 10.1080/10635150490445706

[13] L. Sallan, A. K. Galimberti, Body-size reduction in vertebrates following the end-Devonian mass extinction. Science 350, 812–815 (2015). 26564854 10.1126/science.aac7373

[14] C. M. Pérez-Ben, R. R. Schoch, A. M. Báez, Miniaturization and morphological evolution in Paleozoic relatives of living amphibians: A quantitative approach. Paleobiology 44, 58–75 (2018).

[15] F. Witzmann, Phylogenetic patterns of character evolution in the hyobranchial apparatus of early tetrapods. Earth Environ. Sci. Trans. R. Soc. Edinb. 104, 145–167 (2013).

[16] F. Witzmann, E. Brainerd, Modeling the physiology of the aquatic temnospondyl *Archegosaurus decheni* from the early Permian of Germany. Foss. Rec. 20, 105–127 (2017).

[17] R. R. Schoch, F. Witzmann, The evolution of larvae in temnospondyls and the stepwise origin of amphibian metamorphosis. Biol. Rev. 99, 1613–1637 (2024). 38599802 10.1111/brv.13084

[18] N. Brocklehurst, D. P. Ford, R. B. J. Benson, Early origins of divergent patterns of morphological evolution on the mammal and reptile stem-lineages. Syst. Biol. 71, 1195–1209 (2022). 35274702 10.1093/sysbio/syac020 PMC9366456

[19] C. A. Marsicano, J. D. Pardo, R. M. H. Smith, A. C. Mancuso, L. C. Gaetano, H. Mocke, Giant stem tetrapod was apex predator in Gondwanan late Palaeozoic ice age. Nature 631, 577–582 (2024). 38961286 10.1038/s41586-024-07572-0

[20] M. Ruta, J. E. Jeffery, M. I. Coates, A supertree of early tetrapods. Proc. Biol. Sci. 270, 2507–2516 (2003). 14667343 10.1098/rspb.2003.2524 PMC1691537

[21] P. L. Godoy, R. B. J. Benson, M. Bronzati, R. J. Butler, The multi-peak adaptive landscape of crocodylomorph body size evolution. BMC Evol. Biol. 19, 167 (2019). 31390981 10.1186/s12862-019-1466-4 PMC6686447

[22] R. R. Schoch, Phylogeny of the amphibamiform temnospondyls: The relationship of taxa known by adults, larvae and neotenes. J. Syst. Palaeontol. 20, 1–30 (2022).

[23] M. Ruta, F. Witzmann, J. Klembara, N. FrÖBisch, Tempo and mode of skull size evolution in Temnospondyli (Tetrapoda: Amphibia) and lineage diversification in the largest group of early tetrapods. Earth Environ. Sci. Trans. R. Soc. Edinb. 115, 212–234 (2024).

[24] R. R. Reisz, J. Fröbisch, The oldest caseid synapsid from the Late Pennsylvanian of Kansas, and the evolution of herbivory in terrestrial vertebrates. PLOS ONE 9, e94518 (2014). 24739998 10.1371/journal.pone.0094518 PMC3989228

[25] T. F. Hansen, Stabilizing selection and the comparative analysis of adaptation. Evolution 51, 1341–1351 (1997). 28568616 10.1111/j.1558-5646.1997.tb01457.x

[26] J. M. Beaulieu, D. C. Jhwueng, C. Boettiger, B. C. O’Meara, Modeling stabilizing selection: Expanding the Ornstein–Uhlenbeck model of adaptive evolution. Evolution 66, 2369–2383 (2012). 22834738 10.1111/j.1558-5646.2012.01619.x

[27] J. Felsenstein, Phylogenies and the comparative method. Am. Nat. 125, 1–15 (1985). 10.1086/703055 31094602

[28] J. A. Clack, An early tetrapod from ‘Romer’s Gap’. Nature 418, 72–76 (2002). 12097908 10.1038/nature00824

[29] J. R. G. Rawson, L. B. Porro, E. Martin-Silverstone, E. J. Rayfield, Osteology and digital reconstruction of the skull of the early tetrapod *Whatcheeria deltae*. J. Vertebr. Paleontol. 41, e1927749 (2021).

[30] R. B. J. Benson, R. A. Frigot, A. Goswami, B. Andres, R. J. Butler, Competition and constraint drove Cope’s rule in the evolution of giant flying reptiles. Nat. Commun. 5, 3567 (2014). 24694584 10.1038/ncomms4567 PMC3988819

[31] R. B. J. Benson, G. Hunt, M. T. Carrano, N. Campione, Cope’s rule and the adaptive landscape of dinosaur body size evolution. Palaeontology 61, 13–48 (2018).

[32] T. A. Stewart, J. B. Lemberg, E. J. Hillan, I. Magallanes, E. B. Daeschler, N. H. Shubin, The axial skeleton of *Tiktaalik roseae*. Proc. Natl. Acad. Sci. U.S.A. 121, e2316106121 (2024). 38564638 10.1073/pnas.2316106121 PMC11009633

[33] D. E. Korneisel, H. C. Maddin, Review of the tetrapod skull–neck boundary: Implications for the evolution of the atlas–axis complex. Biol. Rev. 100, 2435–2470 (2025). 40695774 10.1111/brv.70053 PMC12586291

[34] J. Müller, T. M. Scheyer, J. J. Head, P. M. Barrett, I. Werneburg, P. G. P. Ericson, D. Pol, M. R. Sánchez-Villagra, Homeotic effects, somitogenesis and the evolution of vertebral numbers in recent and fossil amniotes. Proc. Natl. Acad. Sci. U.S.A. 107, 2118–2123 (2010). 20080660 10.1073/pnas.0912622107 PMC2836685

[35] C. Depéret, *Les Transformations Du Monde Animal*, Bibliothèque de philosophie scientifique (Ernest Flammarion, 1907), vol. 1, pp. 1–360. [The Transformations of the Animal World, Library of Scientific Philosophy].

[36] T. R. Smithson, S. P. Wood, J. E. A. Marshall, J. A. Clack, Earliest Carboniferous tetrapod and arthropod faunas from Scotland populate Romer’s Gap. Proc. Natl. Acad. Sci. U.S.A. 109, 4532–4537 (2012). 22393016 10.1073/pnas.1117332109 PMC3311392

[37] J. A. Clack, C. E. Bennett, D. K. Carpenter, S. J. Davies, N. C. Fraser, T. I. Kearsey, J. E. A. Marshall, D. Millward, B. K. A. Otoo, E. J. Reeves, A. J. Ross, M. Ruta, K. Z. Smithson, T. R. Smithson, S. A. Walsh, Phylogenetic and environmental context of a Tournaisian tetrapod fauna. Nat. Ecol. Evol. 1, 2 (2016). 28812555 10.1038/s41559-016-0002

[38] R. L. Cieri, S. T. Hatch, J. G. Capano, E. L. Brainerd, Locomotor rib kinematics in two species of lizards and a new hypothesis for the evolution of aspiration breathing in amniotes. Sci. Rep. 10, 7739 (2020). 32398656 10.1038/s41598-020-64140-y PMC7217971

[39] M. E. Feder, W. W. Burggren, Cutaneous gas exchange in vertebrates: Design, patterns, control and implications. Biol. Rev. 60, 1–45 (1985). 3919777 10.1111/j.1469-185x.1985.tb00416.x

[40] D. J. Beerling, Low atmospheric CO_2_ levels during the Permo-Carboniferous glaciation inferred from fossil lycopsids. Proc. Natl. Acad. Sci. U.S.A. 99, 12567–12571 (2002). 12235372 10.1073/pnas.202304999 PMC130500

[41] B. J. Mills, A. J. Krause, I. Jarvis, B. D. Cramer, Evolution of atmospheric O_2_ through the Phanerozoic, revisited. Annu. Rev. Earth Planet. Sci. 51, 253–276 (2023).

[42] J. P. Wilson, I. P. Montañez, J. D. White, W. A. DiMichele, J. C. McElwain, C. J. Poulsen, M. T. Hren, Dynamic Carboniferous tropical forests: New views of plant function and potential for physiological forcing of climate. New Phytol. 215, 1333–1353 (2017). 28742257 10.1111/nph.14700

[43] F. Witzmann, The evolution of the scalation pattern in temnospondyl amphibians. Zool. J. Linn. Soc. 150, 815–834 (2007).

[44] H.-D. Sues, R. R. Reisz, Origins and early evolution of herbivory in tetrapods. Trends Ecol. Evol. 13, 141–145 (1998). 21238234 10.1016/s0169-5347(97)01257-3

[45] P. S. L. Anderson, M. Friedman, M. Ruta, Late to the table: Diversification of tetrapod mandibular biomechanics lagged behind the evolution of terrestriality. Integr. Comp. Biol. 53, 197–208 (2013). 23526337 10.1093/icb/ict006

[46] N. Brocklehurst, R. B. J. Benson, Multiple paths to morphological diversification during the origin of amniotes. Nat. Ecol. Evol. 5, 1243–1249 (2021). 34312521 10.1038/s41559-021-01516-x

[47] M. Ruta, D. Pisani, G. T. Lloyd, M. J. Benton, A supertree of Temnospondyli: Cladogenetic patterns in the most species-rich group of early tetrapods. Proc. Biol. Sci. 274, 3087–3095 (2007). 17925278 10.1098/rspb.2007.1250 PMC2293949

[48] J. A. Clack, T. R. Smithson, M. Ruta, A Mississippian (early Carboniferous) tetrapod showing early diversification of the hindlimbs. Commun. Biol. 5, 283 (2022). 35422092 10.1038/s42003-022-03199-x PMC9010477

[49] S. M. Hellert, D. M. Grossnickle, G. T. Lloyd, C. F. Kammerer, K. D. Angielczyk, Derived faunivores are the forerunners of major synapsid radiations. Nat. Ecol. Evol. 7, 1903–1913 (2023). 37798433 10.1038/s41559-023-02200-y

[50] D. P. Ford, R. B. J. Benson, The phylogeny of early amniotes and the affinities of Parareptilia and Varanopidae. Nat. Ecol. Evol. 4, 57–65 (2020). 31900445 10.1038/s41559-019-1047-3

[51] C. So, J. D. Pardo, A. Mann, A new amphibamiform from the Early Permian of Texas elucidates patterns of cranial diversity among terrestrial amphibamiforms. Zool. J. Linn. Soc. 203, zlae012 (2024).

[52] X. A. Jenkins, H.-D. Sues, S. Webb, Z. Schepis, B. R. Peecook, A. Mann, The recumbirostran *Hapsidopareion lepton* from the early Permian (Cisuralian: Artinskian) of Oklahoma reassessed using HRμCT, and the placement of Recumbirostra on the amniote stem. Pap. Palaeontol. 11, e1610 (2025).

[53] R. R. Schoch, The evolution of major temnospondyl clades: An inclusive phylogenetic analysis. J. Syst. Palaeontol 11, 673–705 (2013).

[54] K. D. Angielczyk, M. Ruta, The roots of amphibian morphospace: A geometric morphometric analysis of Paleozoic temnospondyls. Fieldiana, Life Earth Sci. 2012, 40–58 (2012).

[55] R. Werneburg, F. Witzmann, J. W. Schneider, R. Rößler, A new basal zatracheid temnospondyl from the early Permian Chemnitz Fossil Lagerstätte, central-east Germany. PalZ 97, 105–128 (2023).

[56] R. Werneburg, F. Witzmann, The last eryopids: *Clamorosaurus* and *Syndyodosuchus* from the late Kungurian (Cisuralian, Permian) of Russia revisited. Foss. Rec. 27, 353–380 (2024).

[57] A. Mann, J. D. Pardo, H.-D. Sues, Osteology and phylogenetic position of the diminutive ‘microsaur’ *Odonterpeton triangulare* from the Pennsylvanian of Linton, Ohio, and major features of recumbirostran phylogeny. Zool. J. Linn. Soc. 197, 641–655 (2022).

[58] M. Ruta, A. R. Milner, M. I. Coates, The tetrapod *Caerorhachis bairdi* Holmes and Carroll from the Lower Carboniferous of Scotland. Earth Environ. Sci. Trans. R. Soc. Edinb. 92, 229–261 (2001).

[59] X. A. Jenkins, R. B. Benson, D. P. Ford, C. Browning, V. Fernandez, K. Dollman, T. Gomes, E. Griffiths, J. N. Choiniere, B. R. Peecook, Evolutionary assembly of crown reptile anatomy clarified by late Paleozoic relatives of Neodiapsida. Peer Community J. 5, e89 (2025).

[60] J. D. Pardo, M. Szostakiwskyj, P. E. Ahlberg, J. S. Anderson, Hidden morphological diversity among early tetrapods. Nature 546, 642–645 (2017). 28636600 10.1038/nature22966

[61] F. Ronquist, M. Teslenko, P. van der Mark, D. L. Ayres, A. Darling, S. Höhna, B. Larget, L. Liu, M. A. Suchard, J. P. Huelsenbeck, MrBayes 3.2: Efficient Bayesian phylogenetic inference and model choice across a large model space. Syst. Biol. 61, 539–542 (2012). 22357727 10.1093/sysbio/sys029 PMC3329765

[62] E. M. Dunne, A. Farnsworth, R. B. J. Benson, P. L. Godoy, S. E. Greene, P. J. Valdes, D. J. Lunt, R. J. Butler, Climatic controls on the ecological ascendancy of dinosaurs. Curr. Biol. 33, 206–214.e4 (2023). 36528026 10.1016/j.cub.2022.11.064

[63] D. W. Bapst, paleotree: An R package for paleontological and phylogenetic analyses of evolution. Methods Ecol. Evol. 3, 803–807 (2012).

[64] D. W. Bapst, M. J. Hopkins, Comparing cal3 and other a posteriori time-scaling approaches in a case study with the pterocephaliid trilobites. Paleobiology 43, 49–67 (2017).

[65] D. J. Button, L. E. Zanno, Repeated evolution of divergent modes of herbivory in non-avian dinosaurs. Curr. Biol. 30, 158–168.e4 (2020). 31813611 10.1016/j.cub.2019.10.050

[66] T. R. Simões, S. E. Pierce, Sustained high rates of morphological evolution during the rise of tetrapods. Nat. Ecol. Evol. 5, 1403–1414 (2021). 34426679 10.1038/s41559-021-01532-x

[67] B. M. Gee, TEMNOS (Temnospondyl Evolution, Morphology, Nomenclature, and Other Stuff), v1.1.0, Zenodo (2025); 10.5281/zenodo.16729855.

[68] P. E. Ahlberg, J. A. Clack, H. Blom, The axial skeleton of the Devonian tetrapod *Ichthyostega*. Nature 437, 137–140 (2005). 16136143 10.1038/nature03893

[69] B. K. A. Otoo, J. R. Bolt, R. E. Lombard, K. D. Angielczyk, M. I. Coates, The postcranial anatomy of *Whatcheeria deltae* and its implications for the family Whatcheeriidae. Zool. J. Linn. Soc. 193, 700–745 (2021).

[70] R. Holmes, The skull and axial skeleton of the Lower Permian anthracosauroid amphibian *Archeria crassidisca* Cope. Palaeontographica Abteilung A 207, 161–206 (1989).

[71] M. C. Brough, J. Brough, E. I. White III, The genus *Gephyrostegus*. Philos. Trans. R. Soc. London Ser. B Biol. Sci. 252, 147–165 (1967).

[72] A. S. Romer, L. W. Price, L. I. Price, Review of the Pelycosauria. Geol. Soc. Am. Bull. 28, 1–533 (1940).

[73] Y. Yu, X. Xu, B. J. R. Benson, Data and materials for “Decoupled phenotypic constraints framed by respiratory adaptation in the rise of land vertebrates”, figshare (2025); 10.6084/m9.figshare.29366372. PMC13041750 41921005

[74] L. J. Revell, phytools 2.0: An updated R ecosystem for phylogenetic comparative methods (and other things). PeerJ 12, e16505 (2024). 38192598 10.7717/peerj.16505 PMC10773453

[75] T. Guillerme, N. Cooper, Time for a rethink: Time sub-sampling methods in disparity-through-time analyses. Palaeontology 61, 481–493 (2018).

[76] C. Ritz, A.-N. Spiess, qpcR: An R package for sigmoidal model selection in quantitative real-time polymerase chain reaction analysis. Bioinformatics 24, 1549–1551 (2008). 18482995 10.1093/bioinformatics/btn227

[77] M. W. Pennell, J. M. Eastman, G. J. Slater, J. W. Brown, J. C. Uyeda, R. G. FitzJohn, M. E. Alfaro, L. J. Harmon, geiger v2.0: An expanded suite of methods for fitting macroevolutionary models to phylogenetic trees. Bioinformatics 30, 2216–2218 (2014). 24728855 10.1093/bioinformatics/btu181

[78] R. B. J. Benson, N. E. Campione, M. T. Carrano, P. D. Mannion, C. Sullivan, P. Upchurch, D. C. Evans, Rates of dinosaur body mass evolution indicate 170 million years of sustained ecological innovation on the avian stem lineage. PLOS Biol. 12, e1001853 (2014). 24802911 10.1371/journal.pbio.1001853 PMC4011683

[79] L. s. T. Ho, C. Ané, A linear-time algorithm for Gaussian and non-Gaussian trait evolution models. Syst. Biol. 63, 397–408 (2014). 24500037 10.1093/sysbio/syu005

[80] L. J. Revell, Size-correction and principal components for interspecific comparative studies. Evolution 63, 3258–3268 (2009). 19663993 10.1111/j.1558-5646.2009.00804.x

[81] A. B. Manual, *An Introduction to Statistical Learning with Applications in R* (Springer, 2013).

[82] J. M. Beaulieu, B. C. O’Meara, M. J. Donoghue, Identifying hidden rate changes in the evolution of a binary morphological character: The evolution of plant habit in campanulid angiosperms. Syst. Biol. 62, 725–737 (2013). 23676760 10.1093/sysbio/syt034

