# Supplementary material for: Decoupled phenotypic constraints framed by respiratory adaptation in the rise of land vertebrates
Source: Sci Adv. 2026 Apr 1;12(14):eaeb0801. doi: 10.1126/sciadv.aeb0801 (PMC13041750; doi:10.1126/sciadv.aeb0801)
Supplement: Supplementary file 1 — Figs. S1 to S65 Legend for data S1 [file sciadv.aeb0801_sm.pdf]

Science Advances
AAAS

Supplementary Materials for

Decoupled phenotypic constraints framed by respiratory adaptation in the rise of land vertebrates

Yilun Yu *et al.*

Corresponding author: Yilun Yu, yuyilun@ivpp.ac.cn; Roger B. J. Benson, rbenson@amnh.org

Sci. Adv. **12**, eaeb0801 (2026)
DOI: 10.1126/sciadv.aeb0801

The PDF file includes:

Figs. S1 to S65
Legend for data S1

Other Supplementary Material for this manuscript includes the following:

Data S1

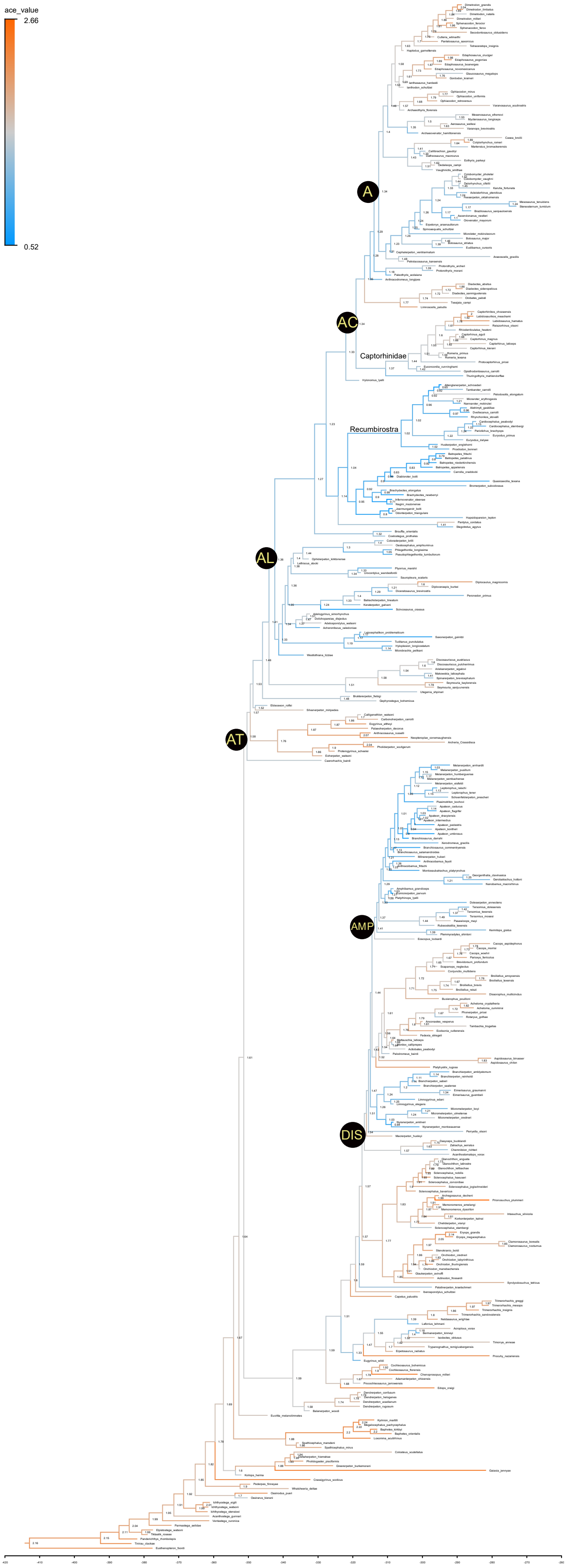
ace_value
2.66
0.52
A
AC
Captorhinidae
Recumbirostra
AL
AT
AMP
DIS

Fig. S1. log10 OSL mapped on dated majority rule consensus tree constrained as **topology 1**. **Abbreviations: AT, Amniotes Total Group; AL, Amniotes + Lepospondyli Clade; AC, Amniotes + Captorhinidae Clade (Only for topology 1 and 2); A, Amniotes; DIS, Dissorophoidea (including Amphibamiformes); AMP, Amphibamiformes.**

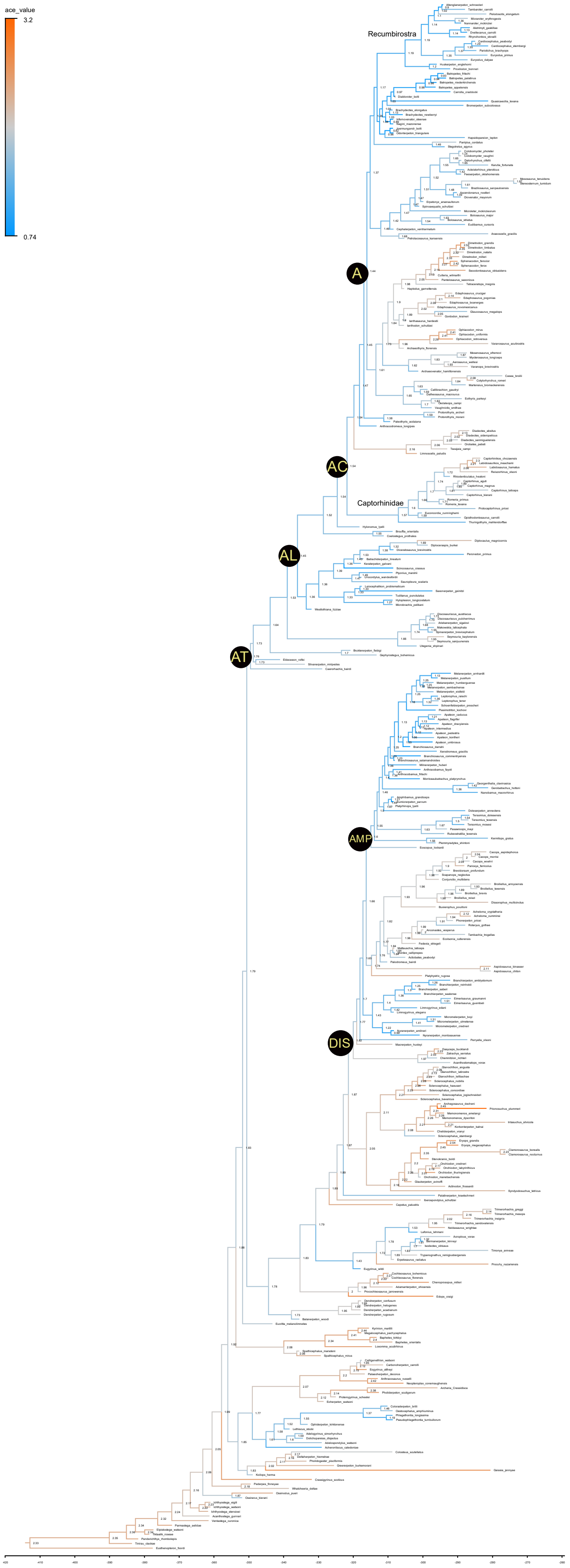
ace_value
3.2
0.74
Recumbirostra
A
AC
Captorhinidae
AL
AT
AMP
DIS

Fig. S2. log10 SL mapped on dated majority rule consensus tree constrained as **topology 2**. **Abbreviations: AT, Amniotes Total Group; AL, Amniotes + Lepospondyli Clade; AC, Amniotes + Captorhinidae Clade (Only for topology 1 and 2); A, Amniotes; DIS, Dissorophoidea (including Amphibamiformes); AMP, Amphibamiformes.**

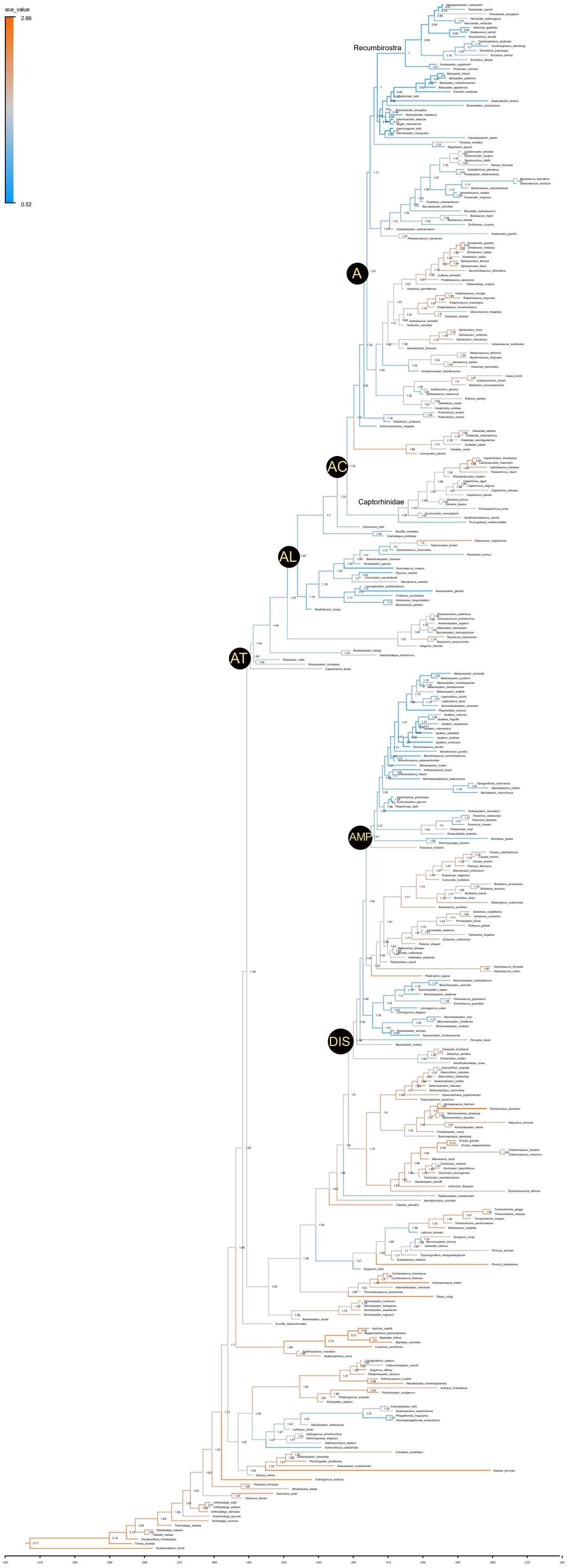

ace_value
2.66
0.52
Recumbirostra
A
AC
Captorhinidae
AL
AT
AMP
DIS

Fig. S3. log10 OSL mapped on dated majority rule consensus tree constrained as **topology 2**. **Abbreviations: AT, Amniotes Total Group; AL, Amniotes + Lepospondyli Clade; AC, Amniotes + Captorhinidae Clade (Only for topology 1 and 2); A, Amniotes; DIS, Dissorophoidea (including Amphibamiformes); AMP, Amphibamiformes.**

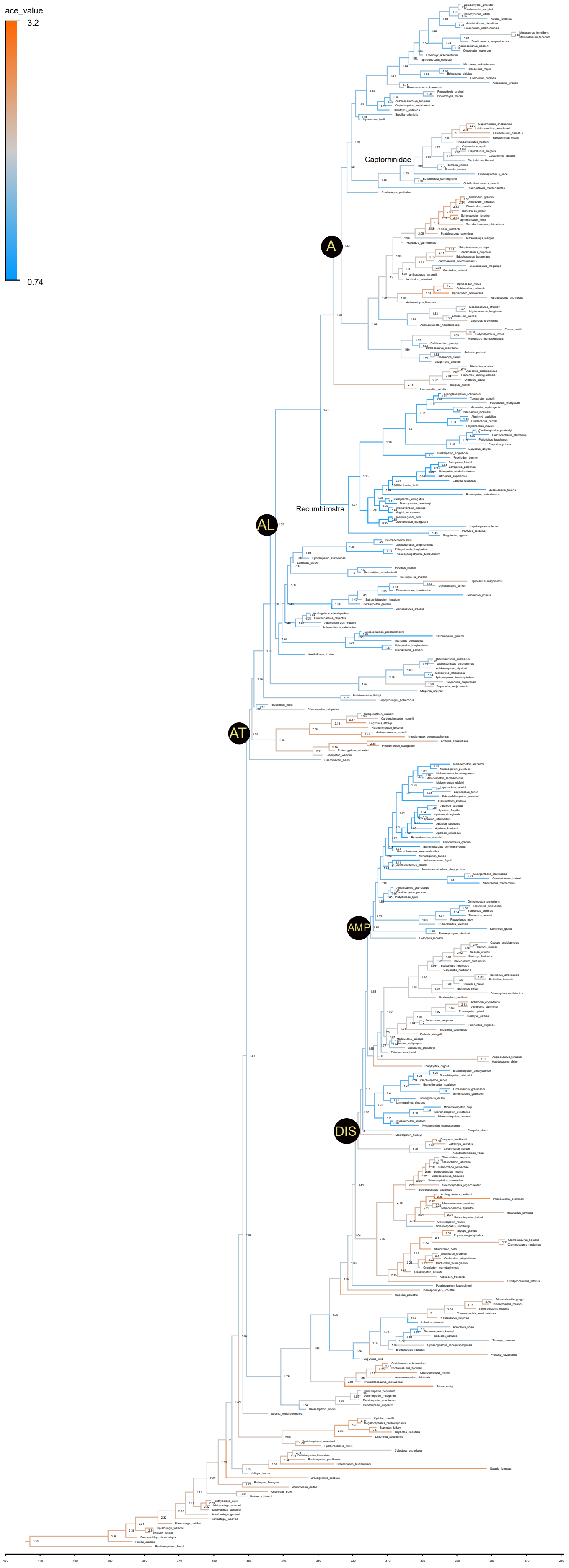
ace_value
3.2
0.74
Captorhinidae
A
Recumbirostra
AL
AT
AMP
DIS

Fig. S4. log10 SL mapped on dated majority rule consensus tree constrained as **topology 3**. **Abbreviations: AT, Amniotes Total Group; AL, Amniotes + Lepospondyli Clade; A, Amniotes; DIS, Dissorophoidea (including Amphibamiformes); AMP, Amphibamiformes.**

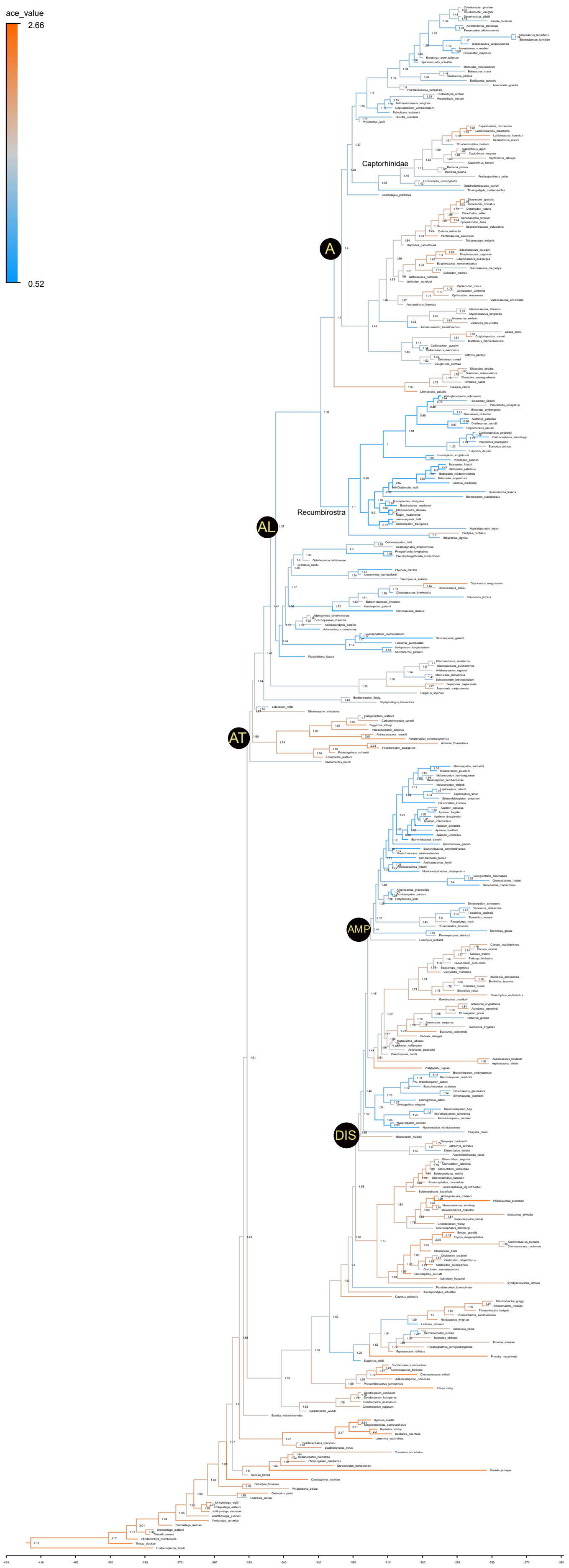
ace_value
2.66
0.52
Captorhinidae
A
Recumbirostra
AL
AT
AMP
DIS

Fig. S5. log10 OSL mapped on dated majority rule consensus tree constrained as **topology 3**. **Abbreviations: AT, Amniotes Total Group; AL, Amniotes + Lepospondyli Clade; A, Amniotes; DIS, Dissorophoidea (including Amphibamiformes); AMP, Amphibamiformes.**

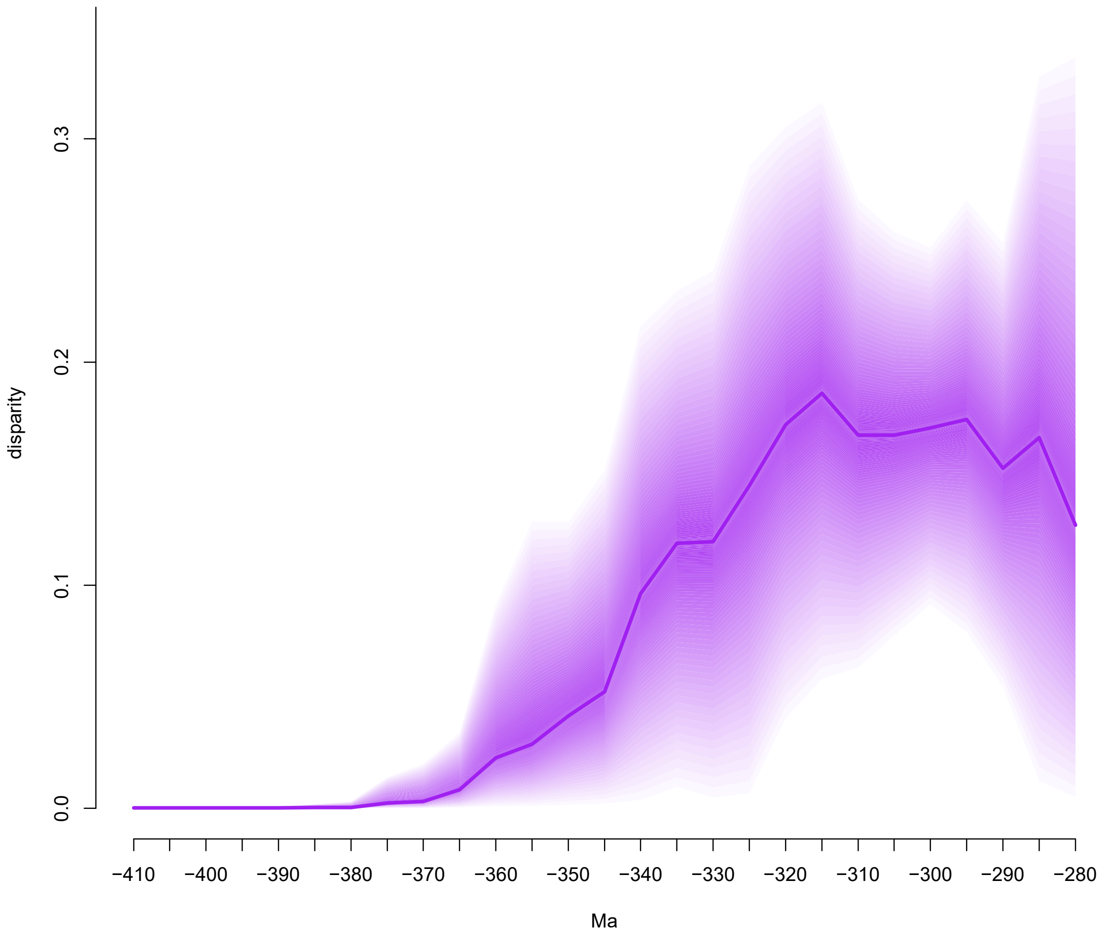

Fig. S6. log10 SL disparity summarized with equal-split model using dated trees constrained with **topology 1**.

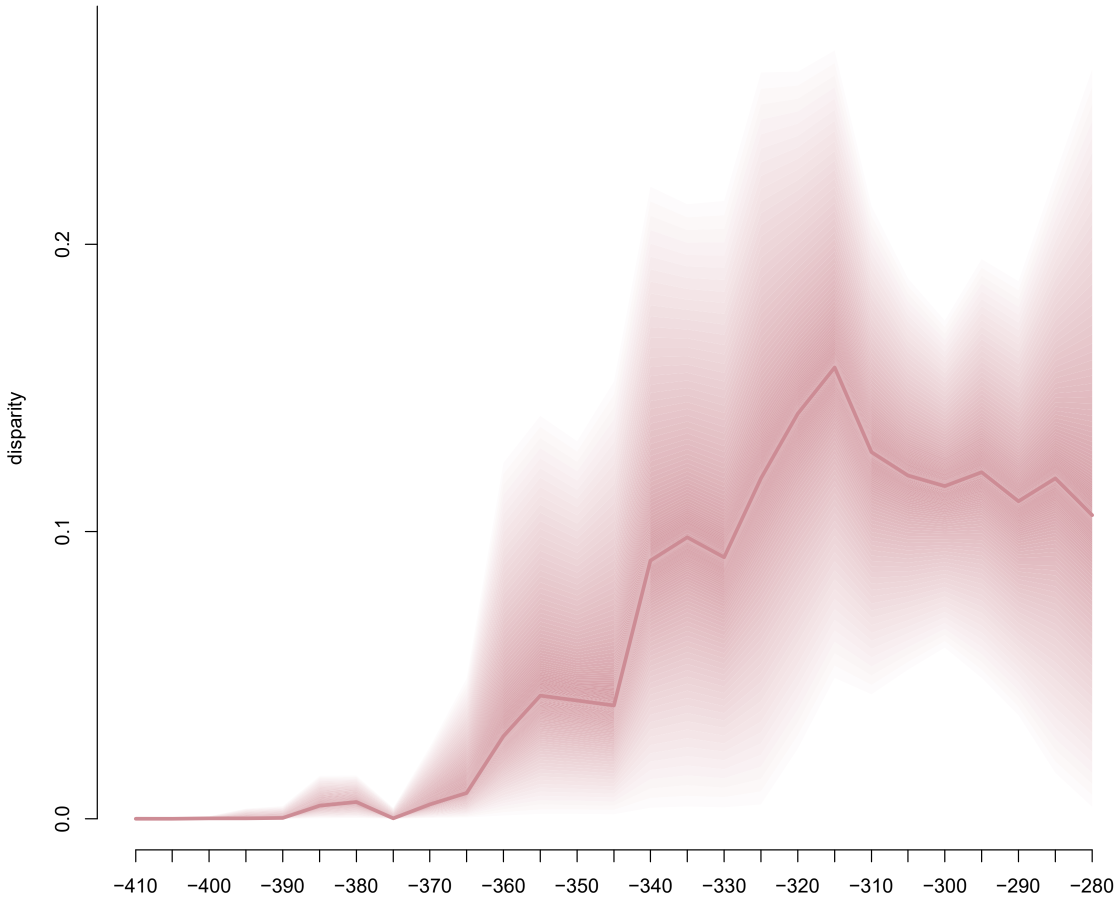

disparity
0.0
0.1
0.2
−410
−400
−390
−380
−370
−360
−350
−340
−330
−320
−310
−300
−290
−280

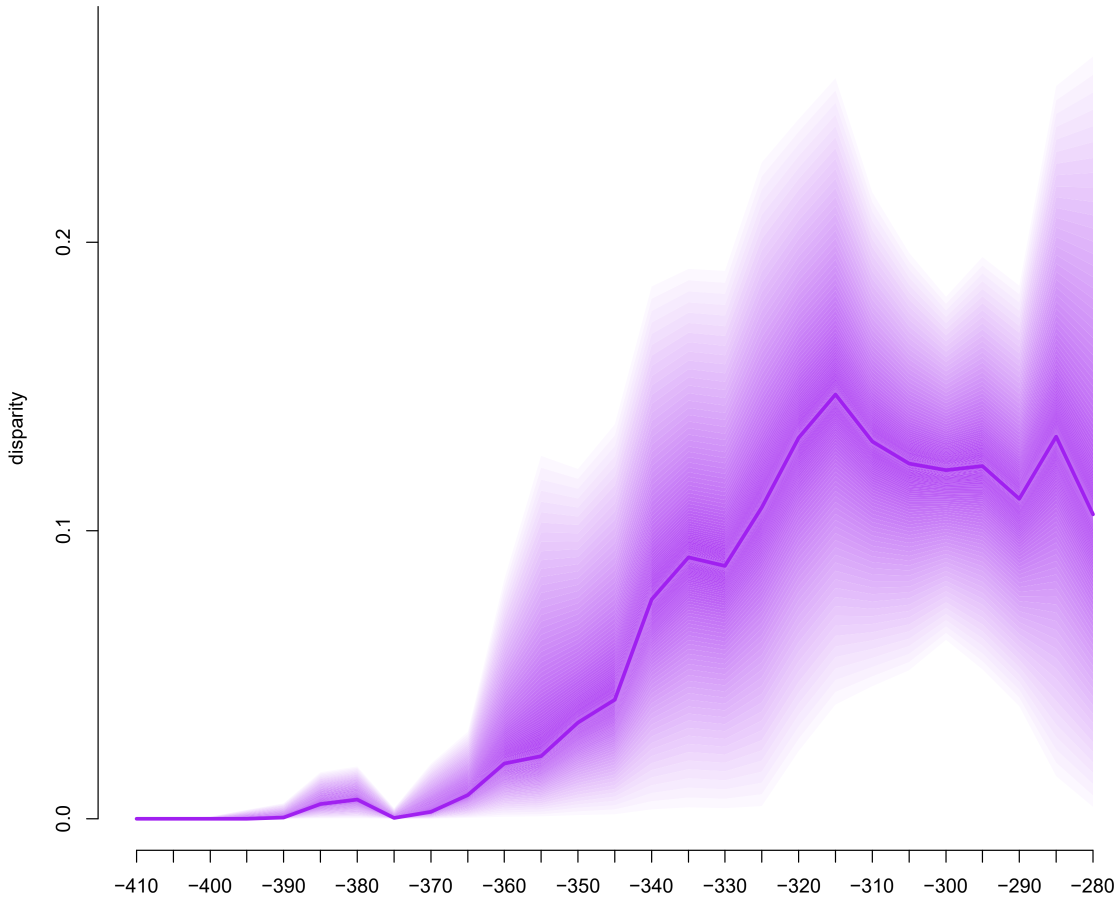

disparity
0.0
0.1
0.2
−410
−400
−390
−380
−370
−360
−350
−340
−330
−320
−310
−300
−290
−280

Fig. S7. log10 OSL disparity summarized with both gradual-split (upper) and equal-split model (lower) using dated trees constrained with **topology 1**.

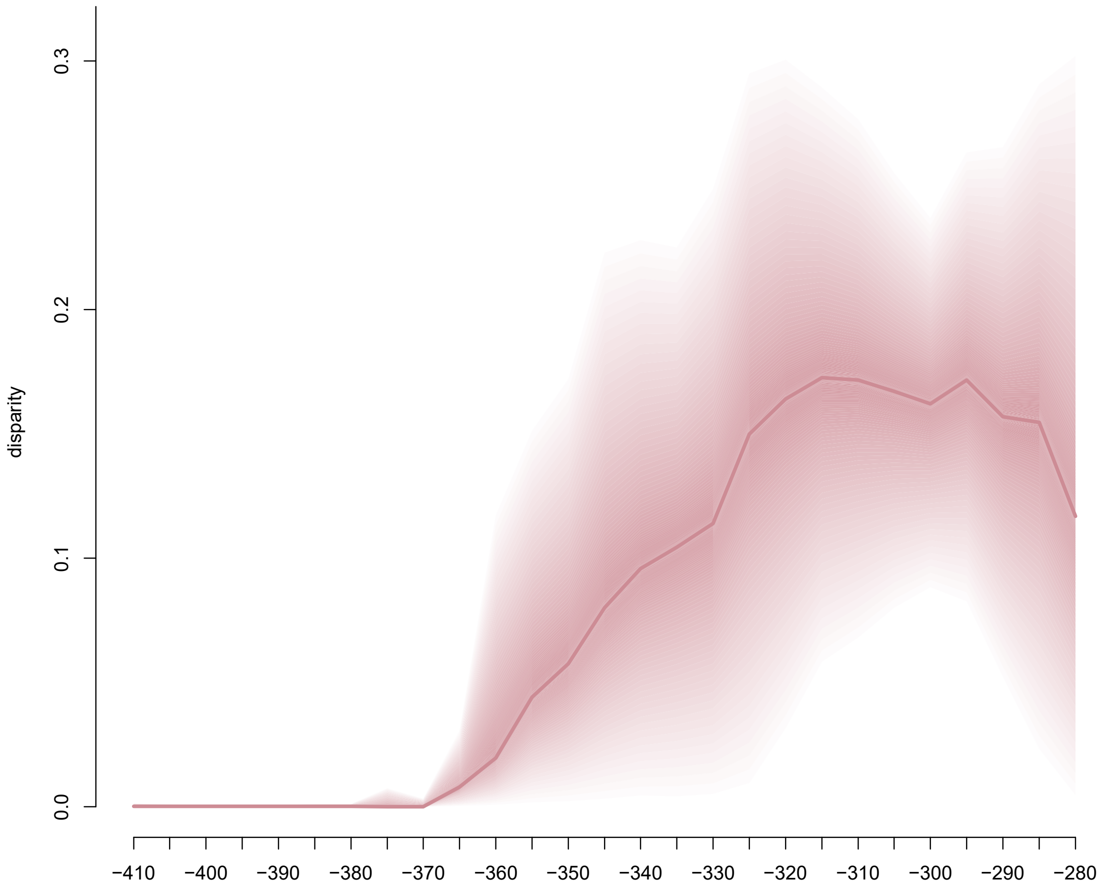
disparity
0.0
0.1
0.2
0.3
−410
−400
−390
−380
−370
−360
−350
−340
−330
−320
−310
−300
−290
−280

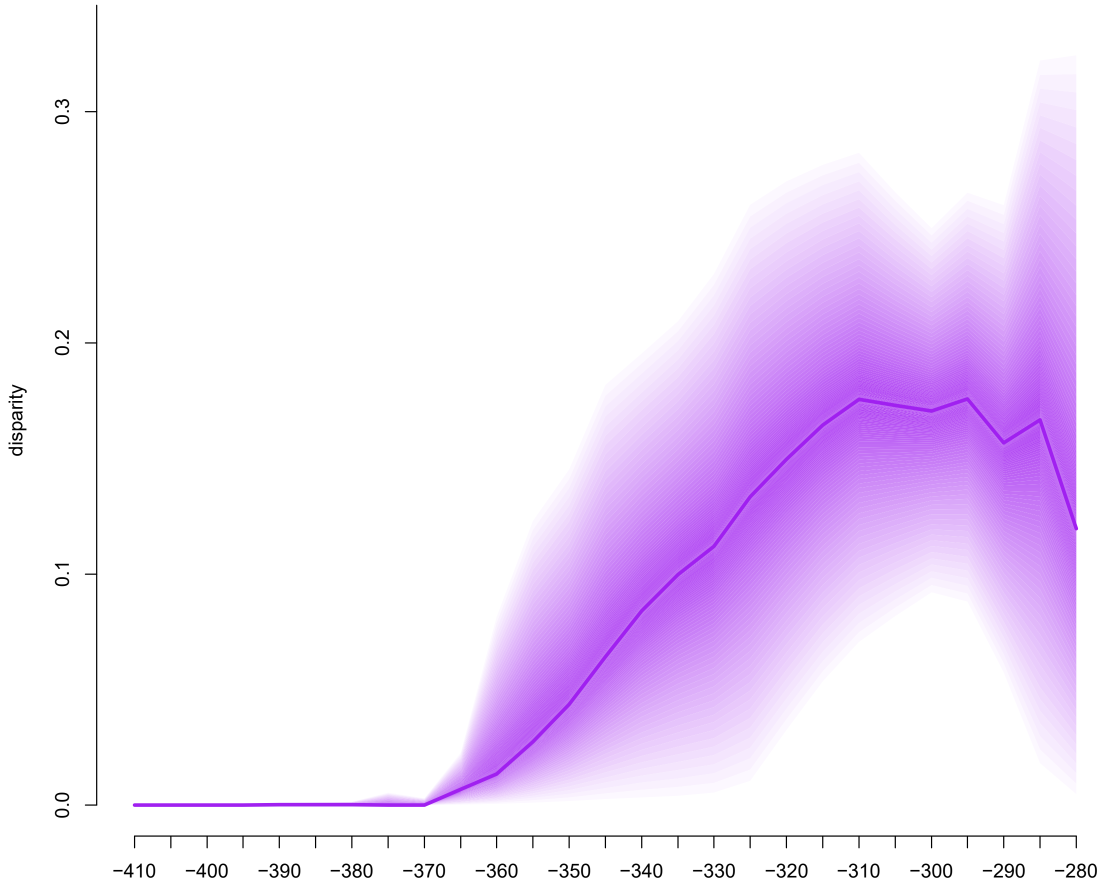
disparity
0.0
0.1
0.2
0.3
−410
−400
−390
−380
−370
−360
−350
−340
−330
−320
−310
−300
−290
−280

Fig. S8. log10 SL disparity summarized with both gradual-split (upper) and equal-split model (lower) using dated trees constrained with **topology 2**.

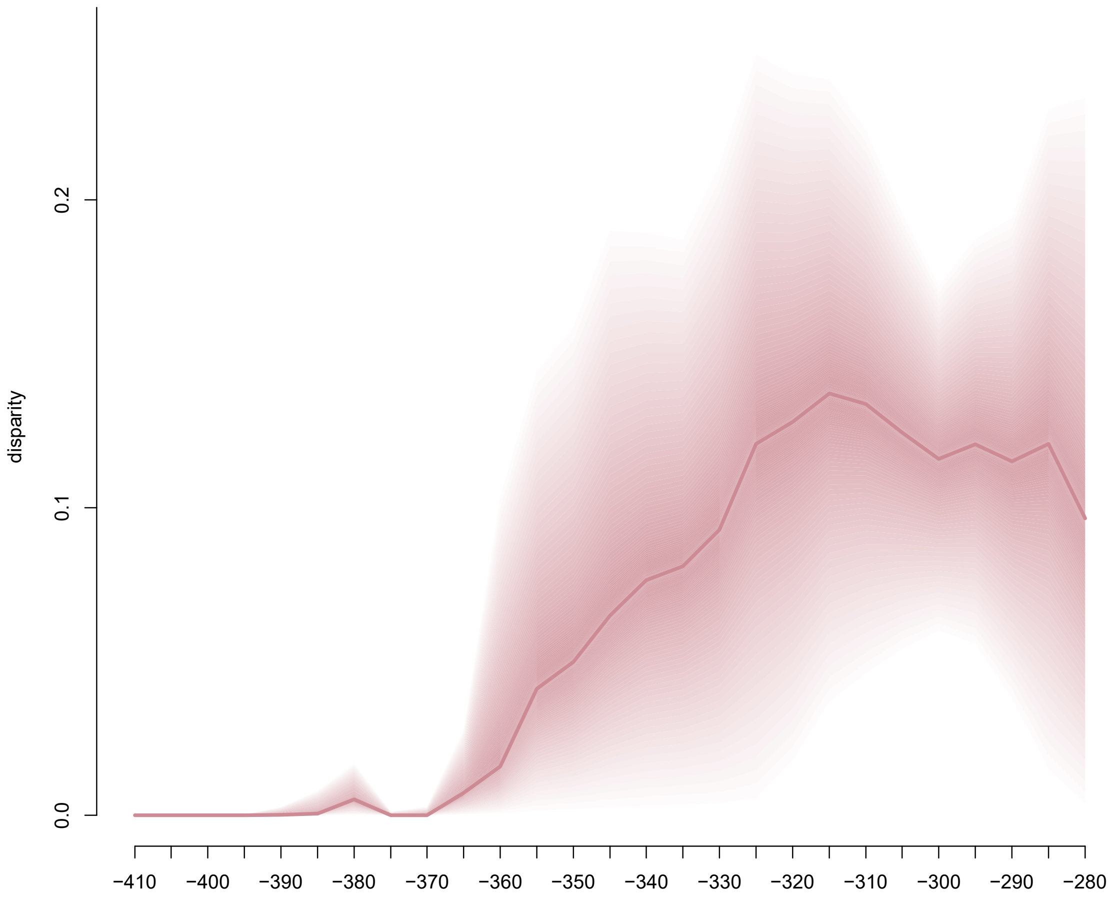

disparity
0.0
0.1
0.2
−410
−400
−390
−380
−370
−360
−350
−340
−330
−320
−310
−300
−290
−280

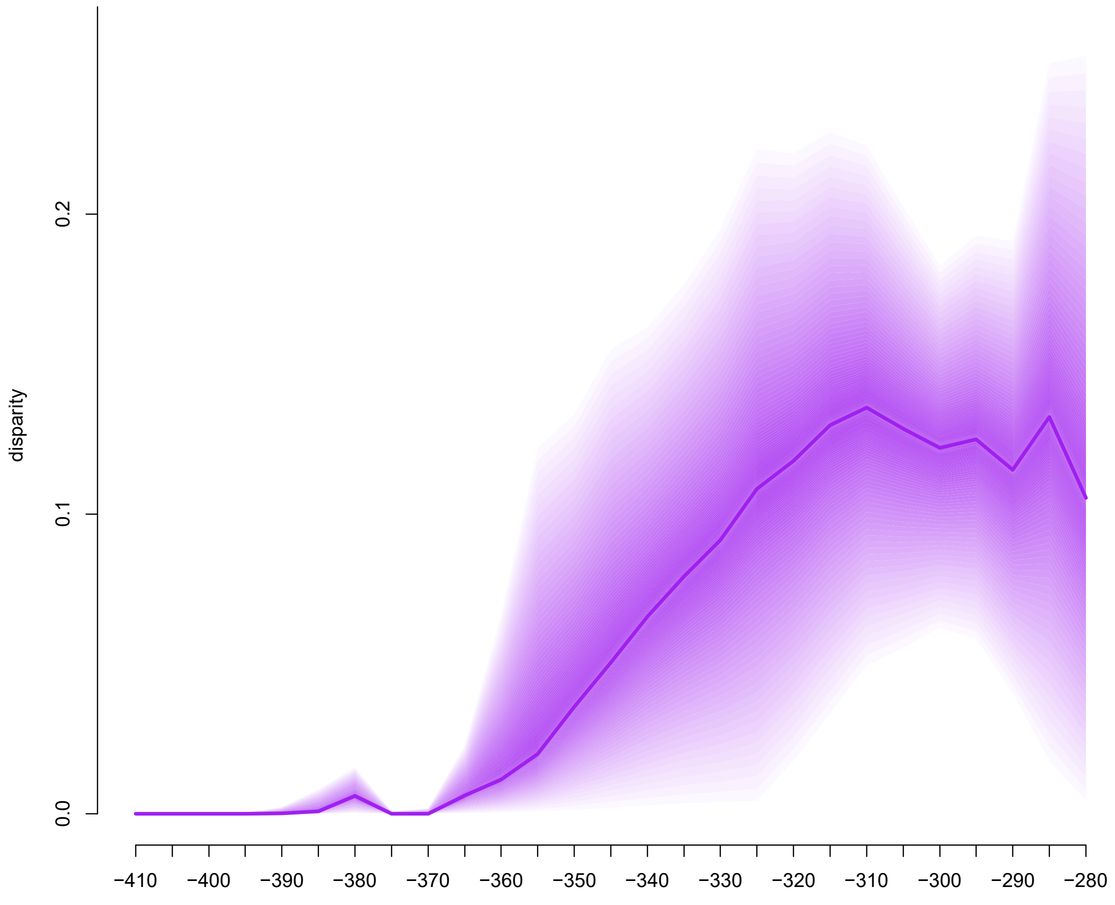

disparity
0.0
0.1
0.2
−410
−400
−390
−380
−370
−360
−350
−340
−330
−320
−310
−300
−290
−280

Fig. S9. log10 OSL disparity summarized with both gradual-split (upper) and equal-split model (lower) using dated trees constrained with **topology 2**.

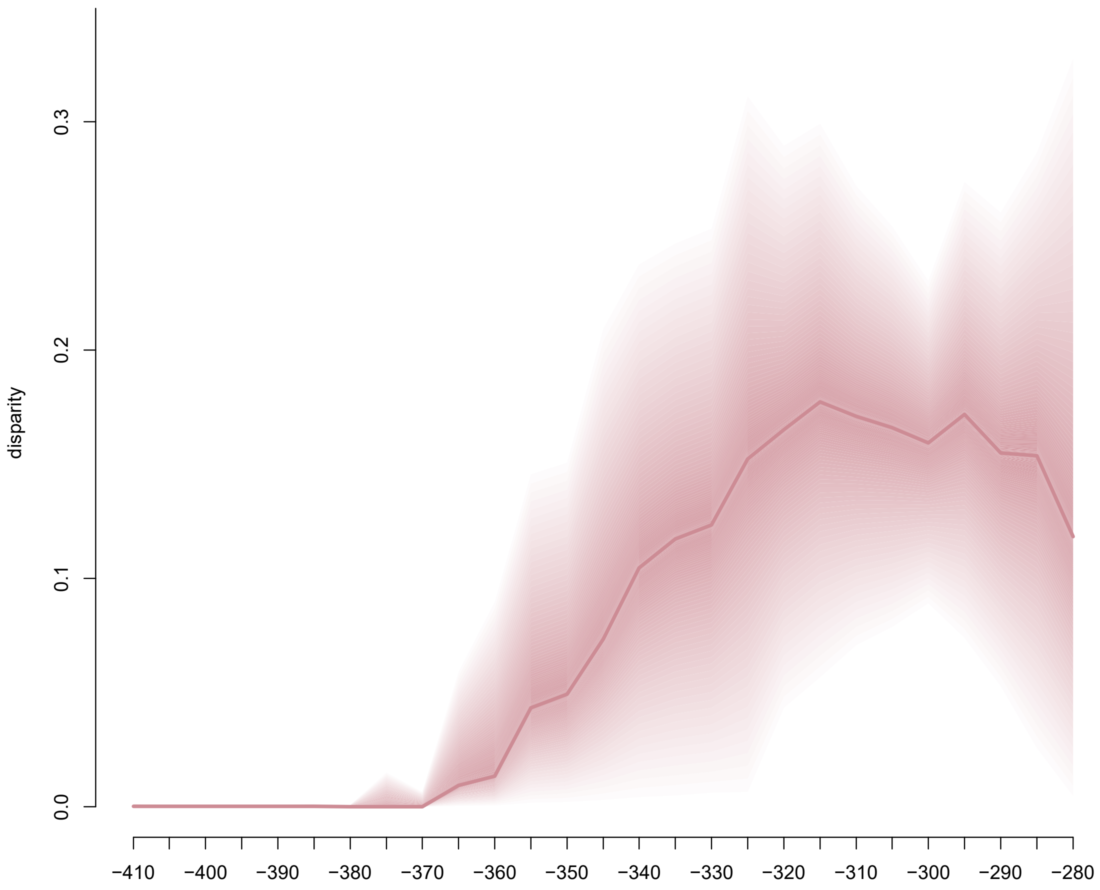

disparity
0.0
0.1
0.2
0.3
−410
−400
−390
−380
−370
−360
−350
−340
−330
−320
−310
−300
−290
−280

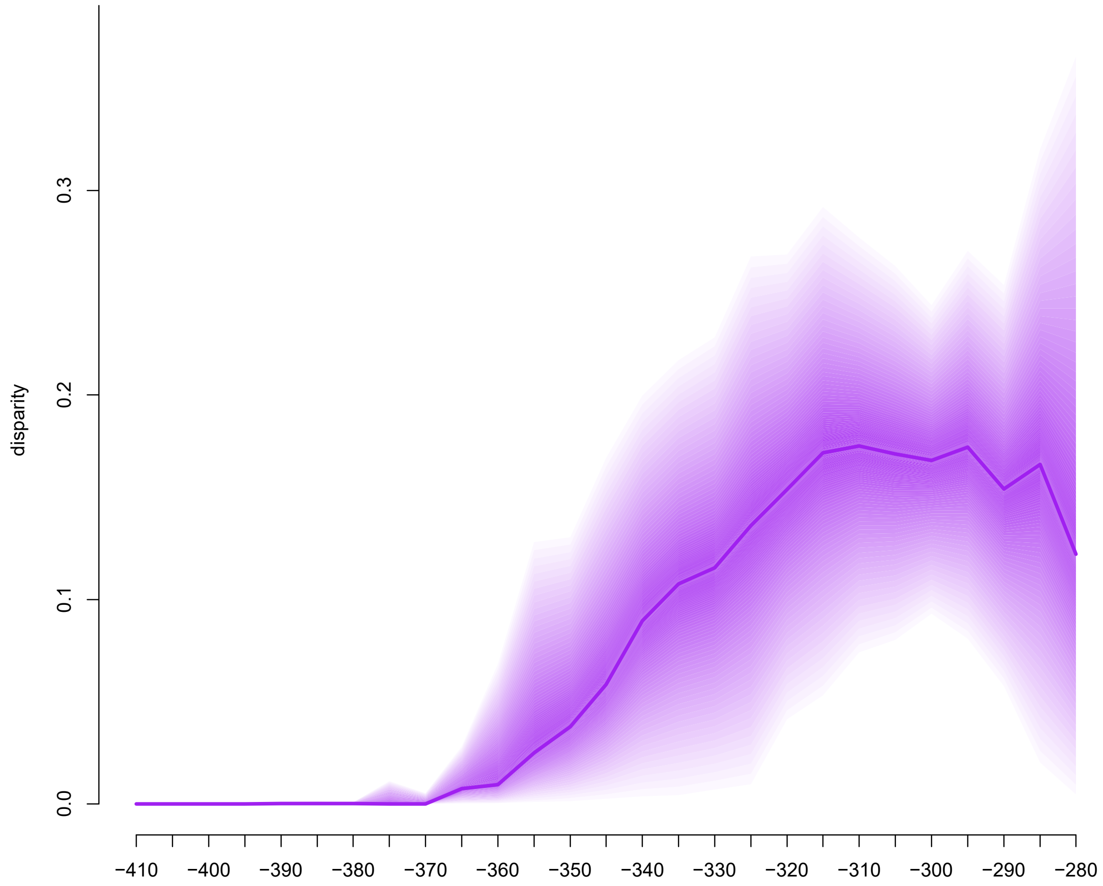

disparity
0.0
0.1
0.2
0.3
−410
−400
−390
−380
−370
−360
−350
−340
−330
−320
−310
−300
−290
−280

Fig. S10. log10 SL disparity summarized with both gradual-split (upper) and equal-split model (lower) using dated trees constrained with **topology 3**.

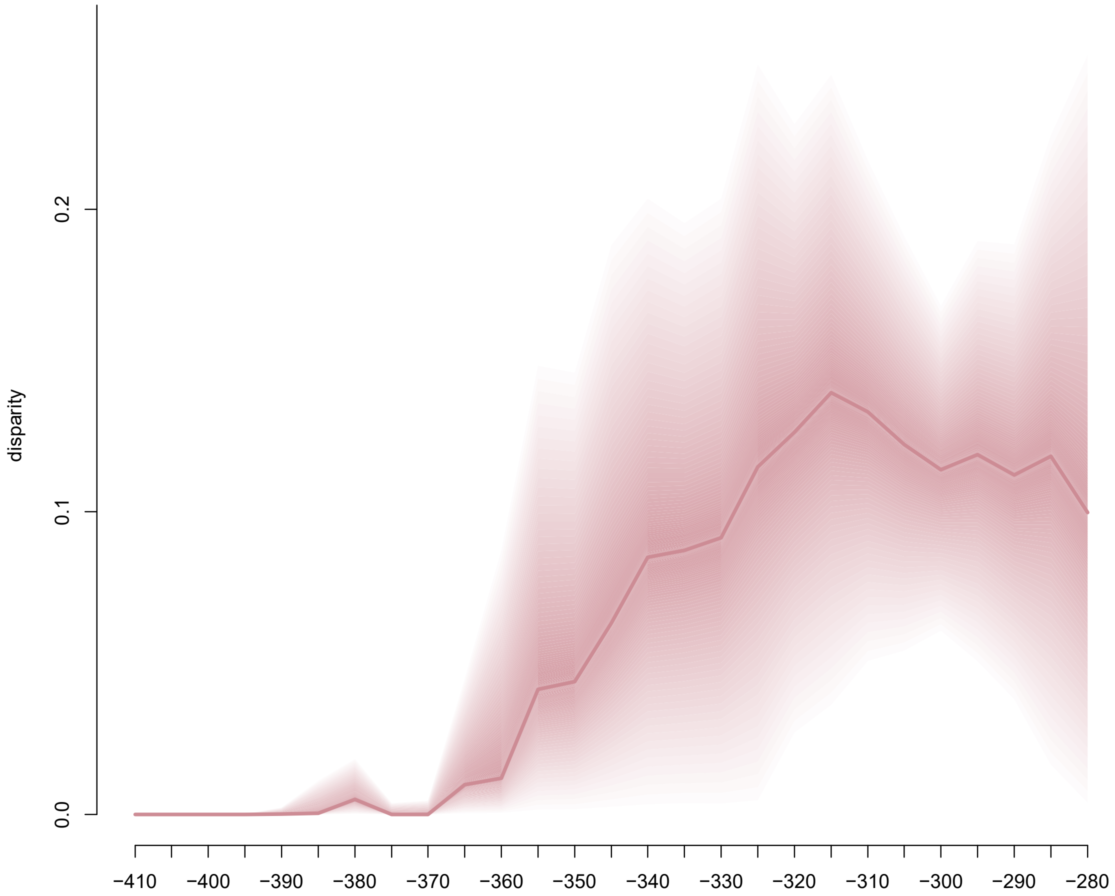
disparity
0.0
0.1
0.2
−410
−400
−390
−380
−370
−360
−350
−340
−330
−320
−310
−300
−290
−280

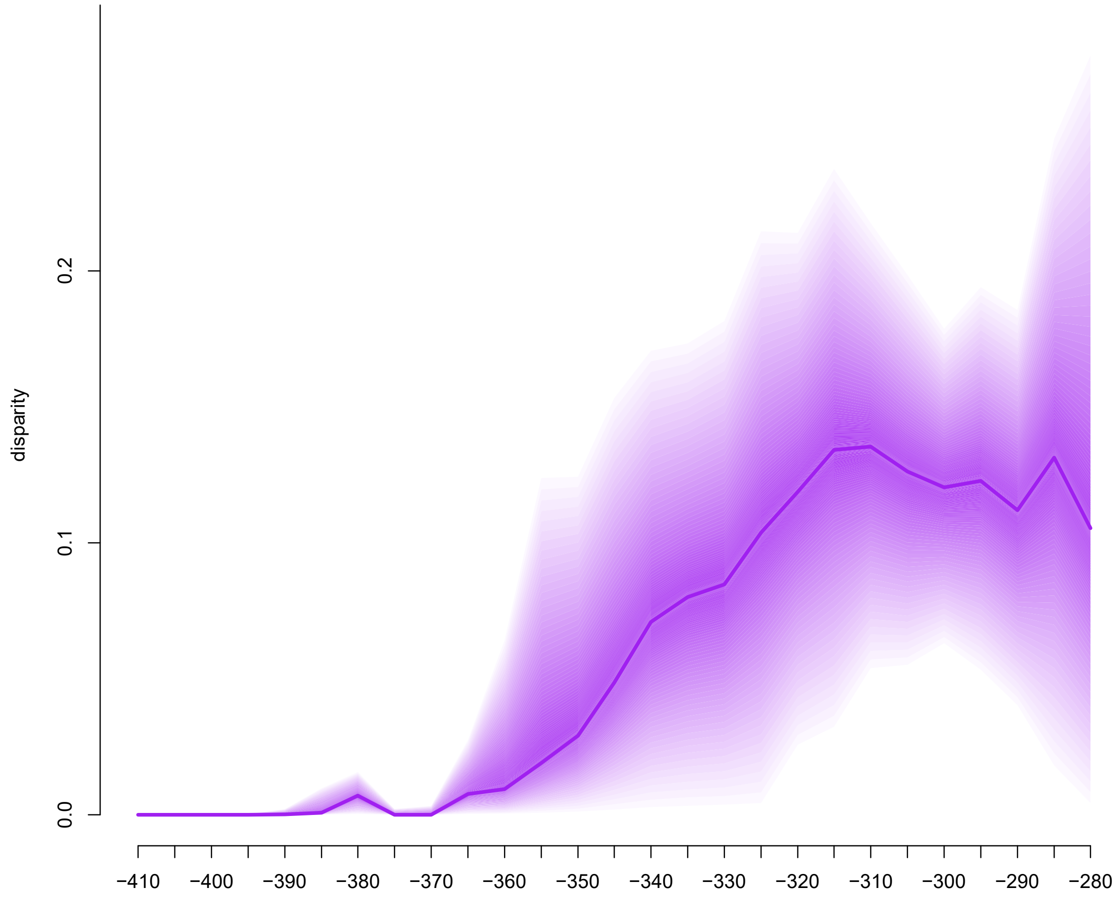
disparity
0.0
0.1
0.2
−410
−400
−390
−380
−370
−360
−350
−340
−330
−320
−310
−300
−290
−280

Fig. S11. log10 OSL disparity summarized with both gradual-split (upper) and equal-split model (lower) using dated trees constrained with **topology 3**.

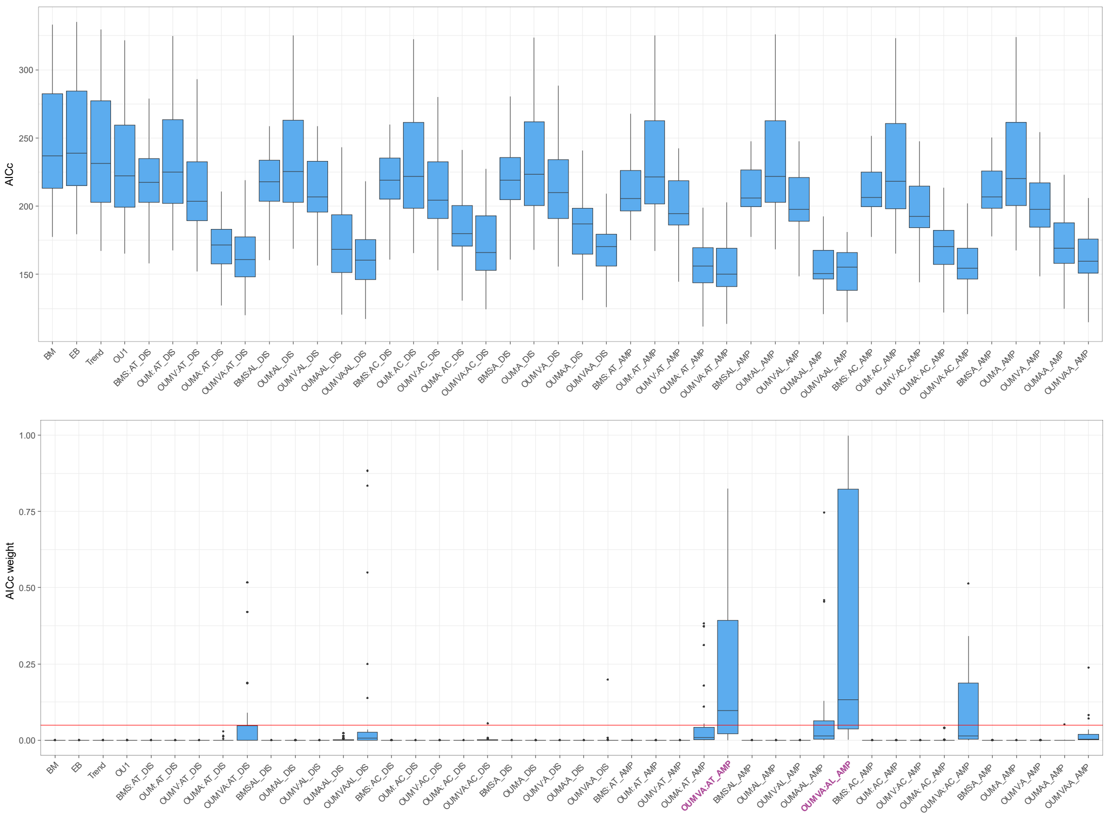

Fig. S12. AICc weight and AICc value of different uniform models and three-regime models, using Log 10 SL as body size index and dated trees constrained as **topology 1**. Red line occurs at 0.05 of AICc weight. **Abbreviations for nodes defining regime-shifts see Fig S1.**

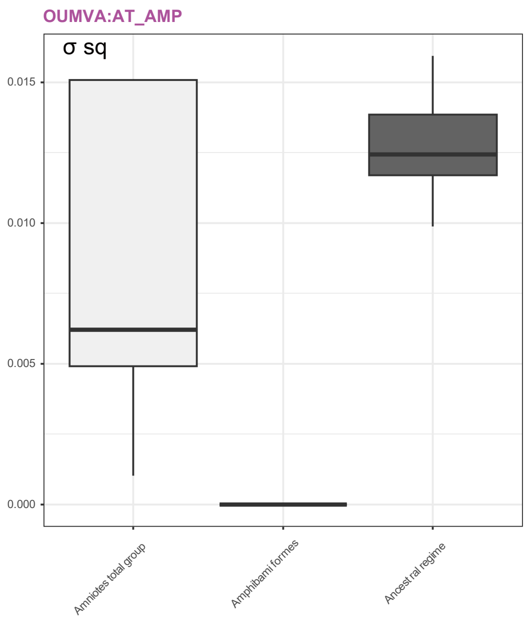

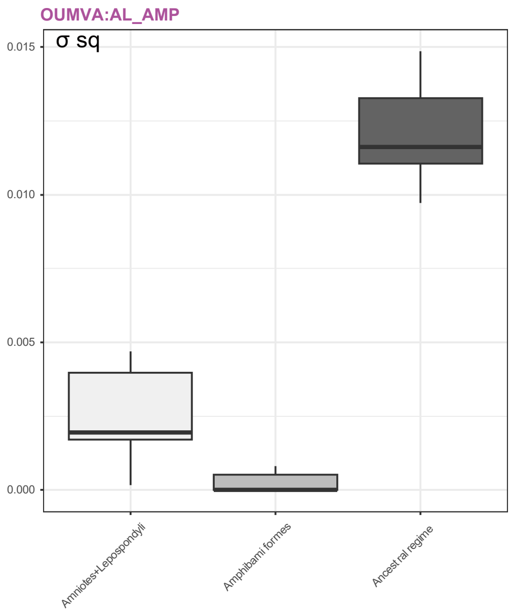

Fig. S13. Sigma estimated from non-negligible models (OUMVA allowing regime-shifts occurred at amniote total-group and Amphibamiformes; OUMVA allowing regime-shifts occurred at amniotes+lepospondyls and Amphibamiformes) of analyses based on log10 SL and dated trees constrained as **topology1**. **For other parameters, see main text figure 2.**

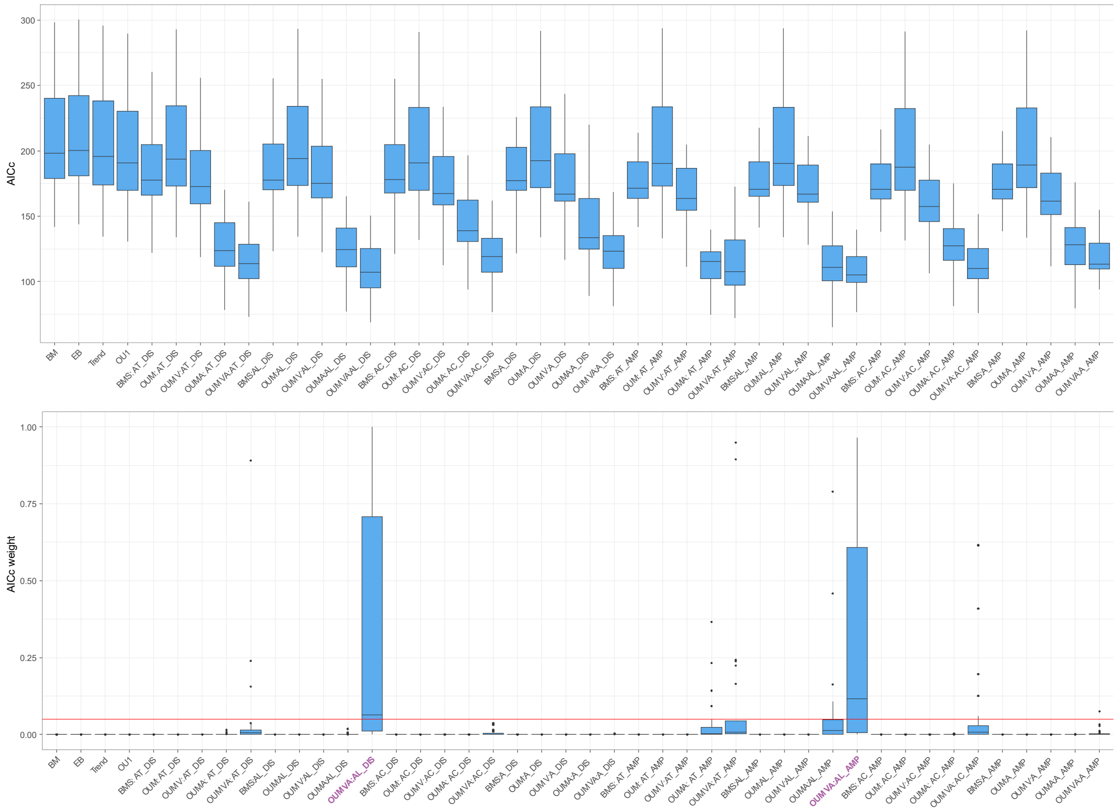

Fig. S14. AICc weight and AICc value of different uniform models and three-regime models, using Log 10 OSL as body size index and dated trees constrained as **topology 1**. Red line occurs at 0.05 of AICc weight. **Abbreviations for nodes defining regime-shifts see Fig S1.**

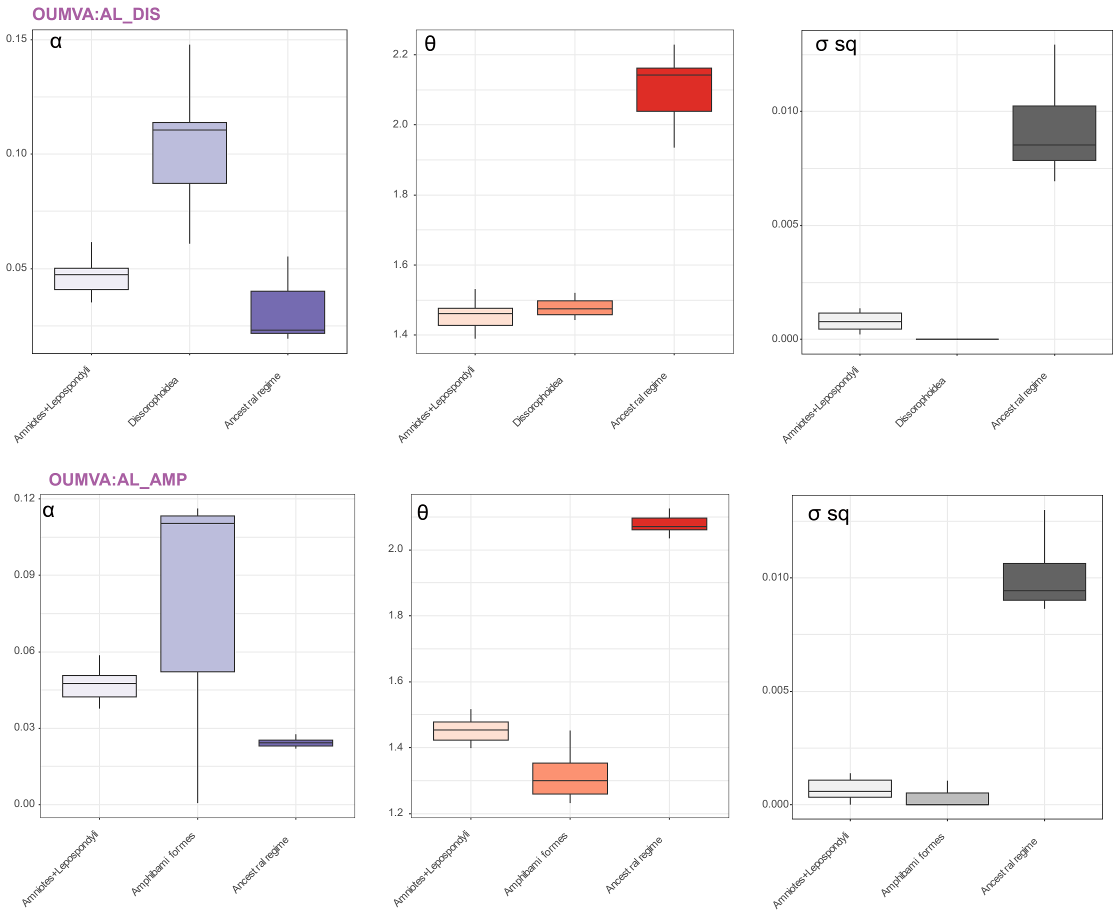

Fig. S15. Parameters estimated from non-negligible models (OUMVA allowing regime-shifts occurred at amniotes+lepospondyls and Dissorophoidea; OUMVA allowing regime-shifts occurred at amniotes+lepospondyls and Amphibamiformes), using Log 10 OSL as body size index and dated trees constrained as **topology 1. Abbreviations for nodes defining regime shifts see Fig S1.**

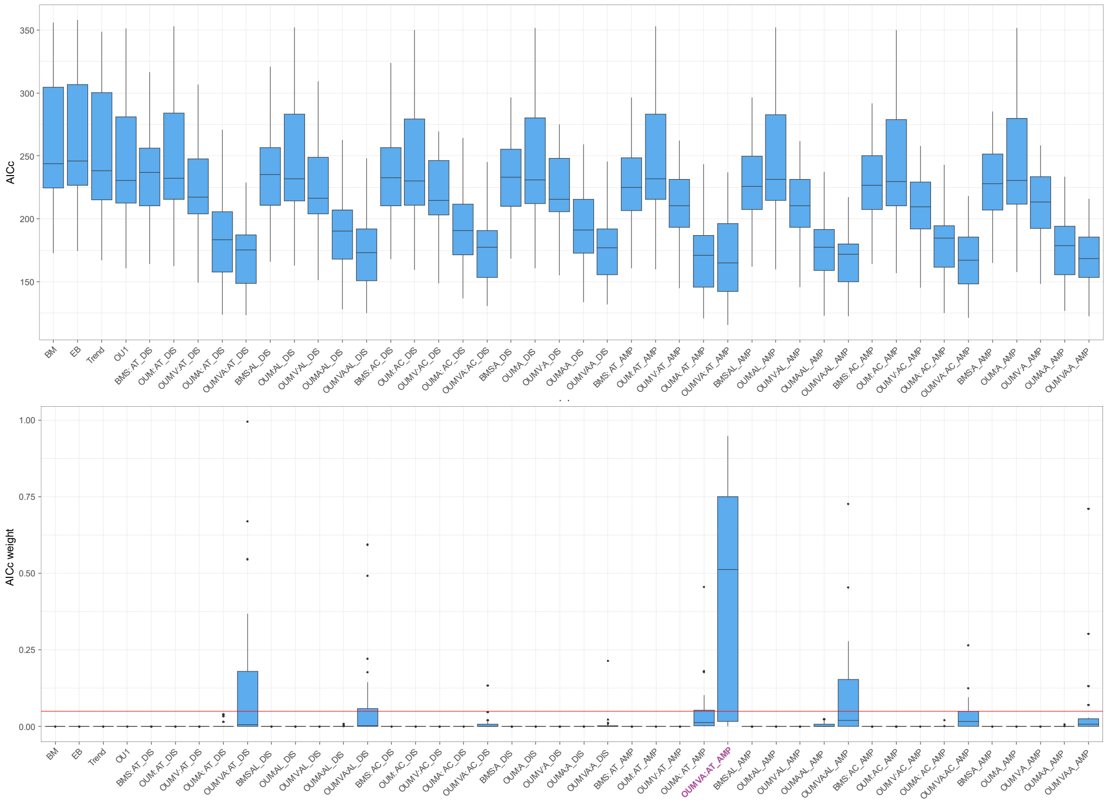

Fig. S16. AICc weight and AICc value of different uniform models and three-regime models, using Log 10 SL and dated trees constrained as **topology 2. Abbreviations for nodes defining regime-shifts see Fig S2.**

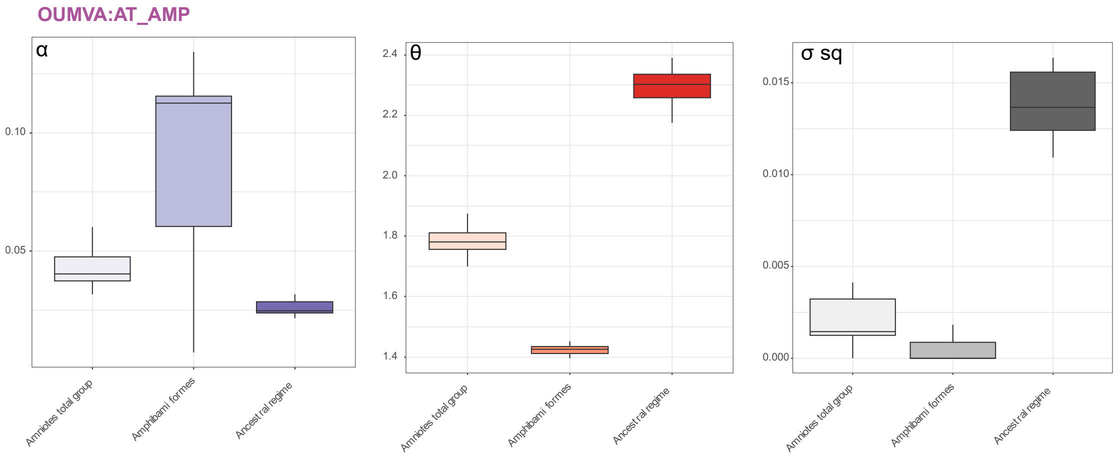

Fig. S17. Parameters estimated from non-negligible models (OUMVA allowing regime-shifts occurred at amniote total-group and Amphibamiformes), using Log 10 SL as body size index and dated trees constrained as **topology 2. Abbreviations for nodes defining regime-shifts see Fig S2.**

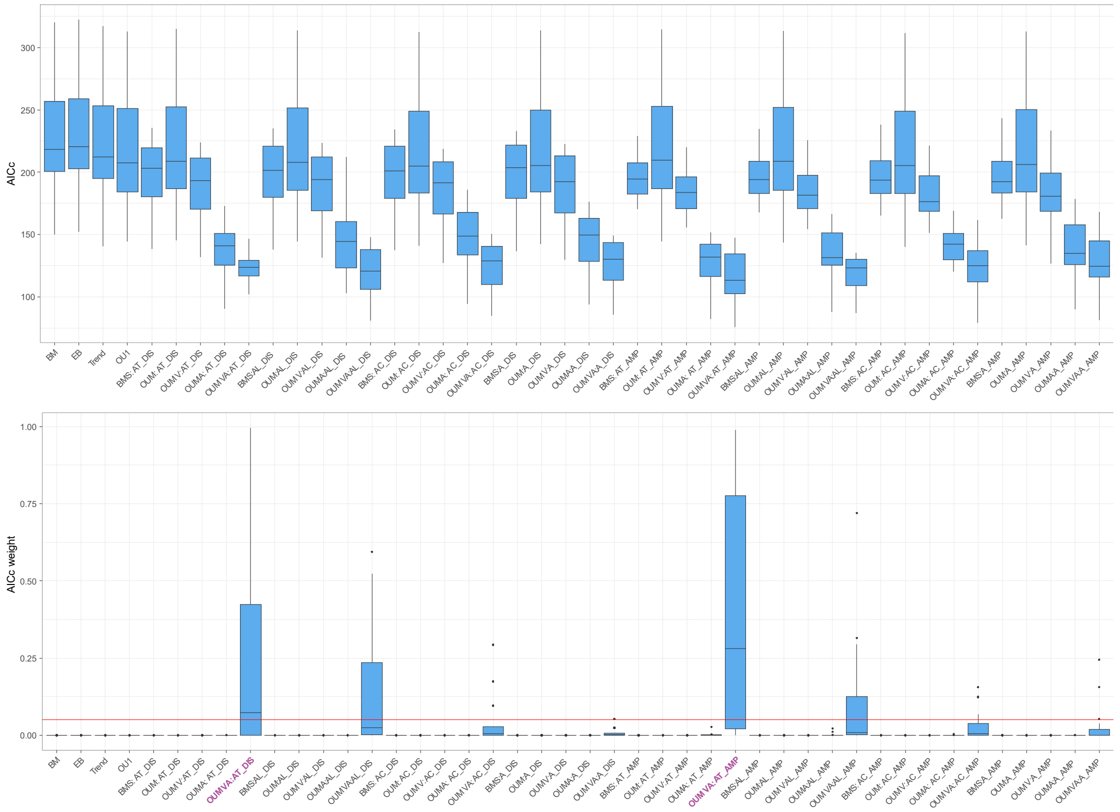

Fig. S18. AICc weight and AICc value of different uniform models and three-regime models, using Log 10 OSL as body size index and dated trees constrained as **topology 2**. Red line occurs at 0.05 of AICc weight. **Abbreviations for nodes defining regime-shifts see Fig S2.**

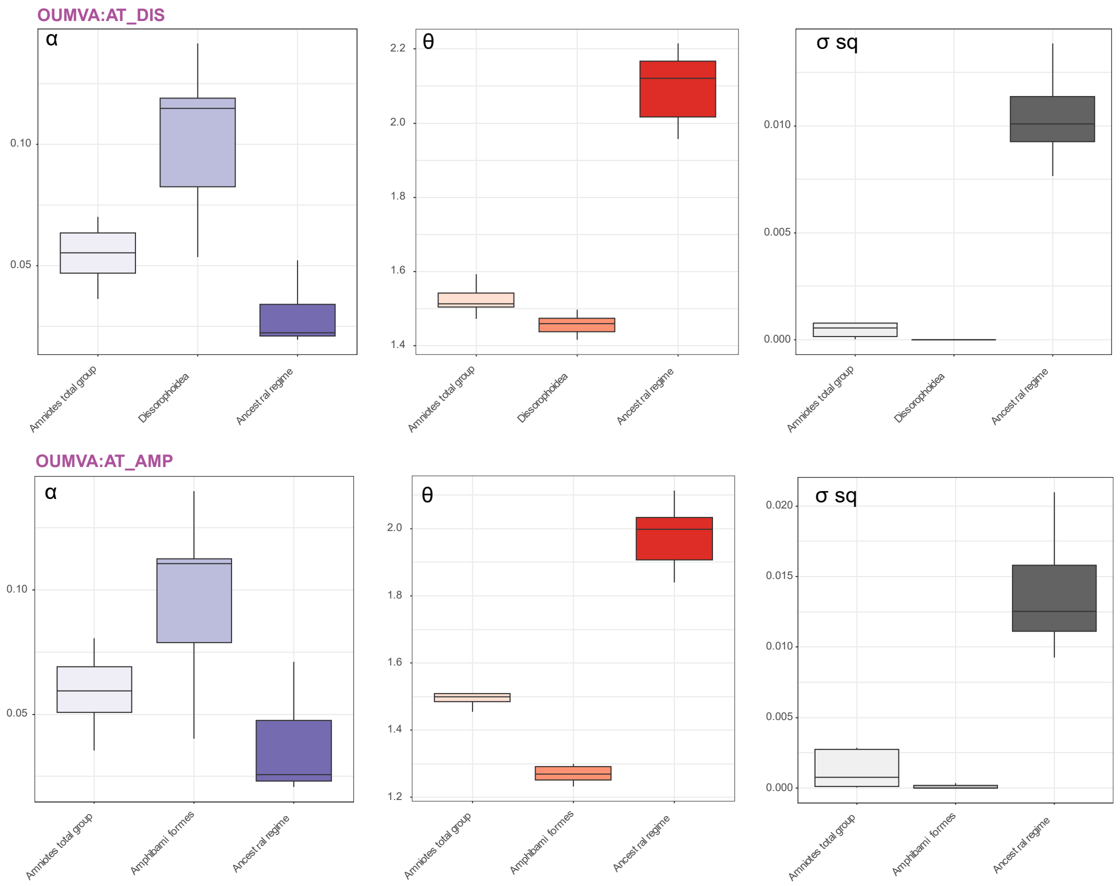

Fig. S19. Parameters estimated from non-negligible model (OUMVA allowing regime-shifts occurred at amniote total-group and Dissorophoidea; OUMVA allowing regime-shifts occurred at amniote total-group and Amphibamiformes), using Log 10 OSL and dated trees constrained as **topology 2**. **Abbreviations for nodes defining regime-shifts see Fig S2.**

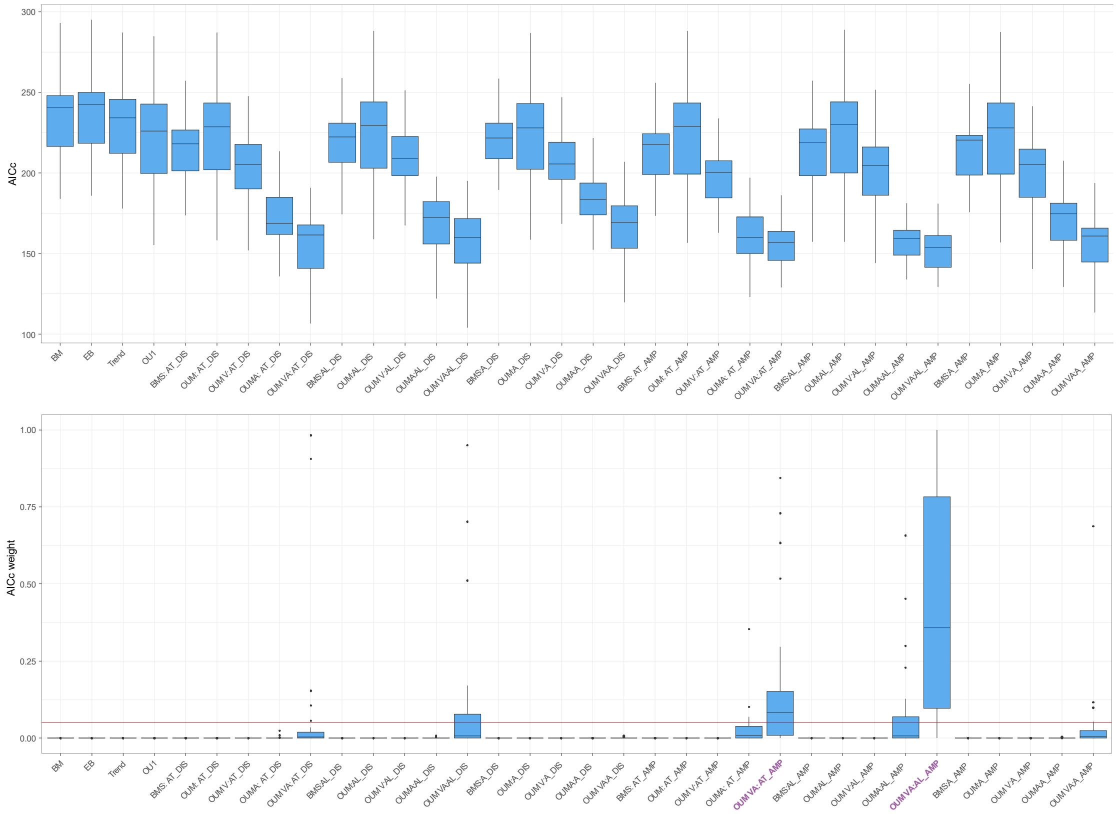

Fig. S20. AICc weight and AICc value of different uniform models and three-regime models, using Log 10 SL as body size index and dated trees constrained as **topology 3**. Red line occurs at 0.05 of AICc weight. **Abbreviations for nodes defining regime-shifts see Fig S4.**

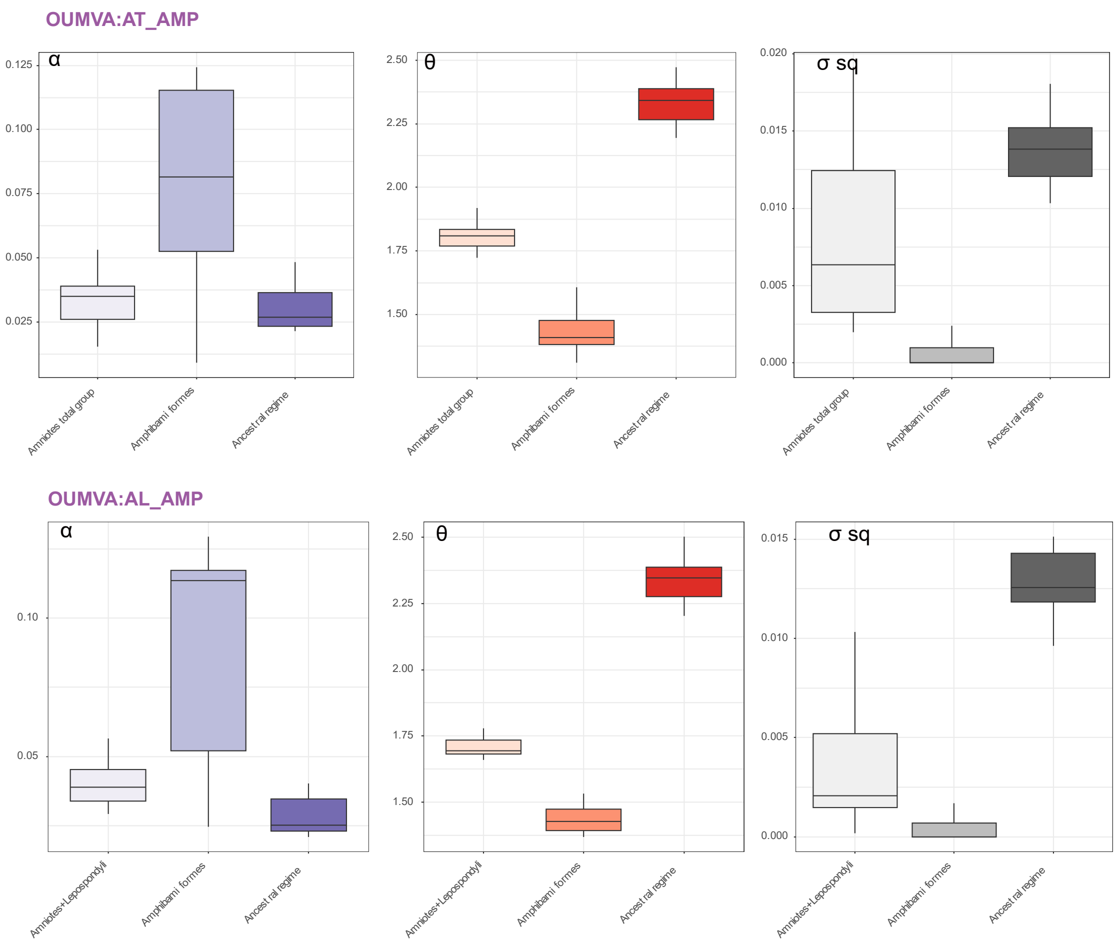

Fig. S21. Parameters estimated from non-negligible model (OUMVA allowing regime-shifts occurred at amniote total-group and Amphibamiformes; OUMVA allowing regime-shifts occurred at amniotes+lepospondyls and Amphibamiformes), using Log 10 SL and dated trees constrained as **topology 3**. **Abbreviations for nodes defining regime-shifts see Fig S4.**

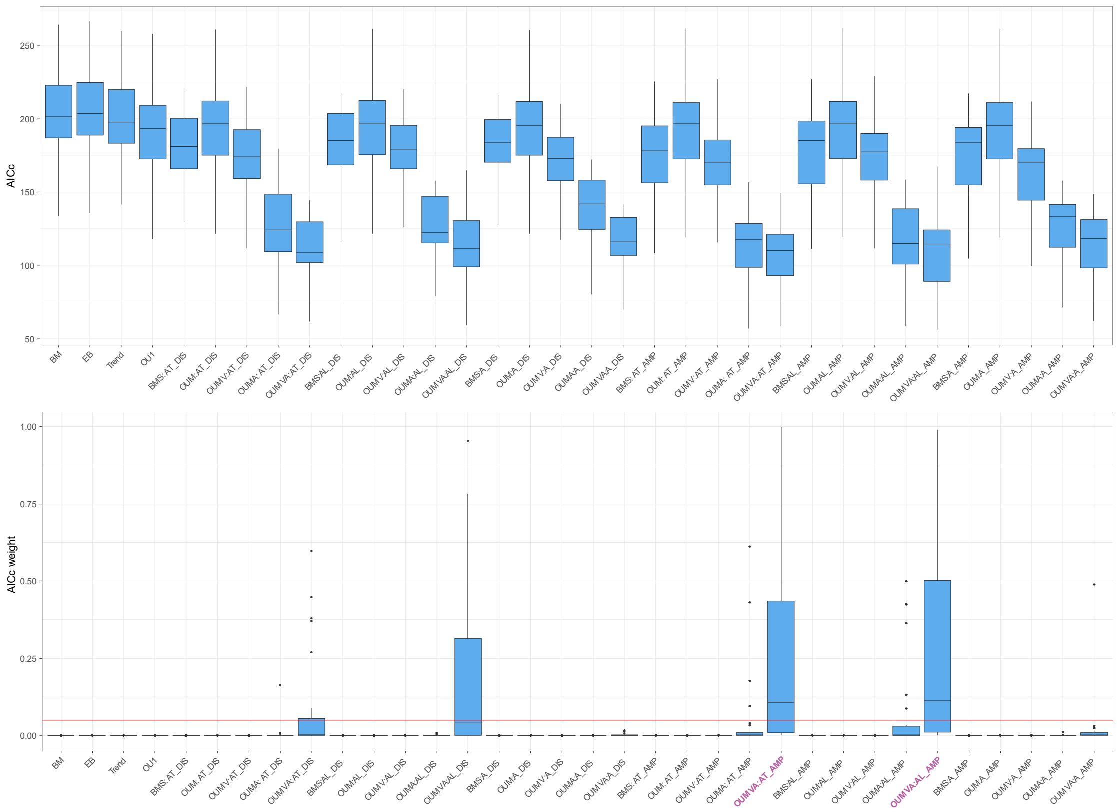

Fig. S22. AICc weight and AICc value of different uniform models and three-regime models, using Log 10 OSL as body size index and dated trees constrained as **topology 3**. Red line occurs at 0.05 of AICc weight. **Abbreviations for nodes defining regime-shifts see Fig S4.**

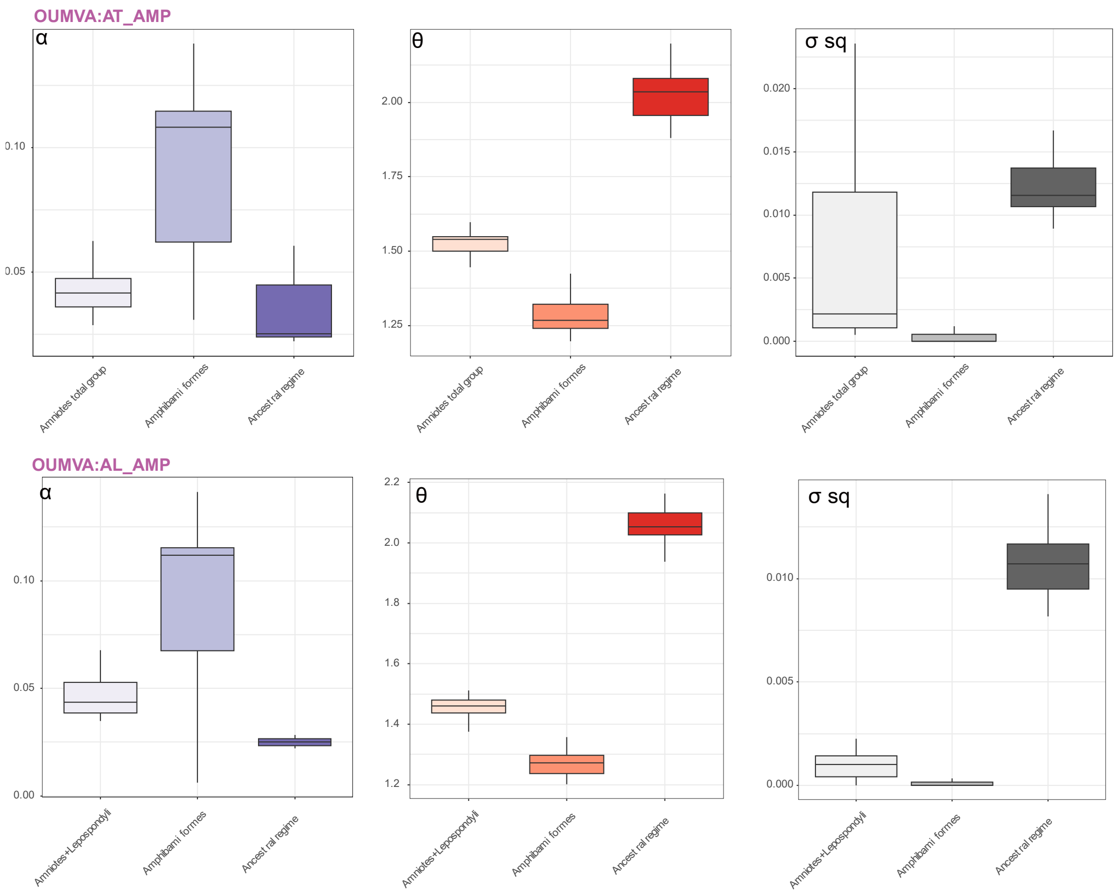

Fig. S23. Parameters estimated from non-negligible model (OUMVA allowing regime-shifts occurred at amniote total-group and Amphibamiformes; OUMVA allowing regime-shifts occurred at amniotes+lepospondyls and Amphibamiformes), using Log 10 OSL and dated trees constrained as **topology 3**. **Abbreviations for nodes defining regime-shifts see Fig S4.**

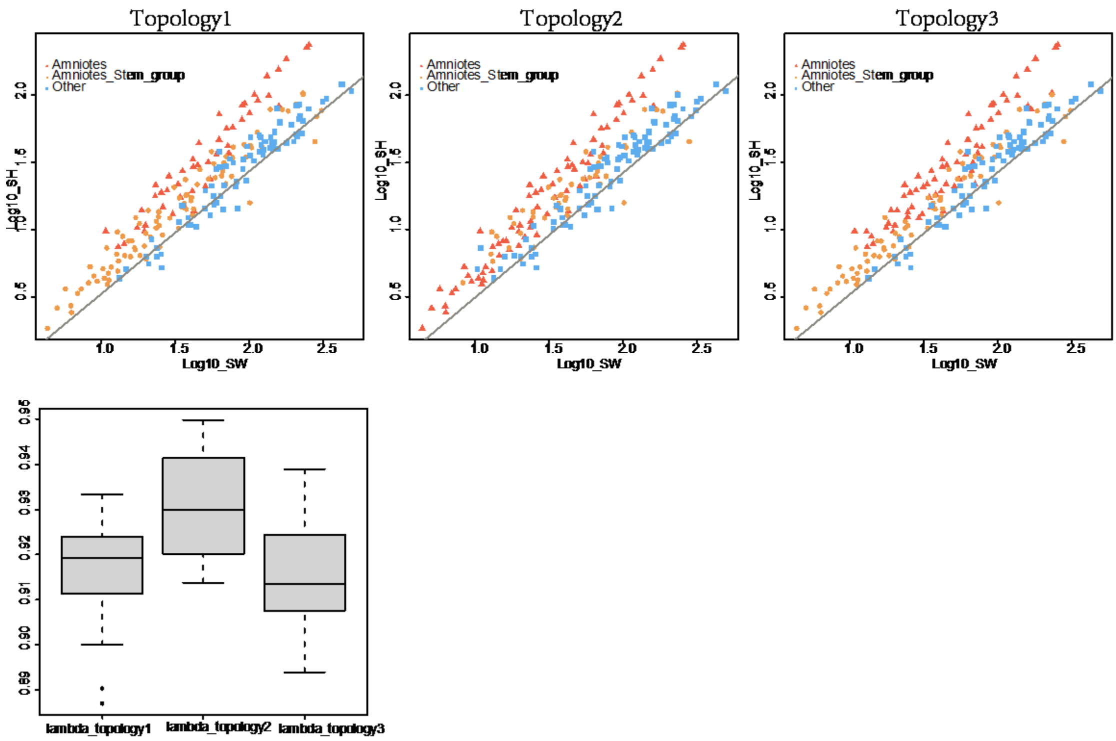

Fig. S24. Phylogenetic linear regression between log10 transformed skull height and skull width and estimated Pagel's lambda.

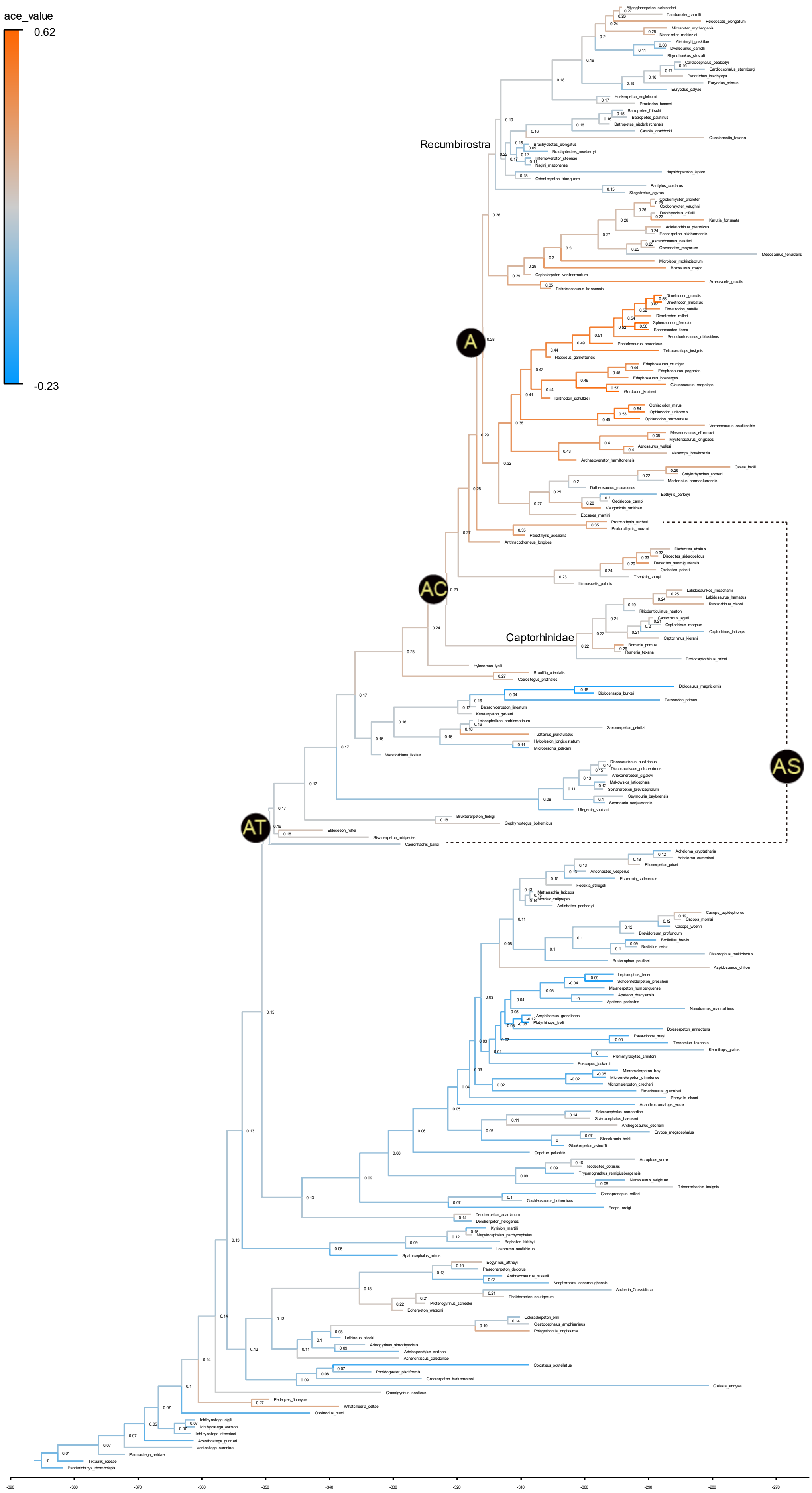
ace_value
0.62
-0.23
Recumbirostra
A
AC
Captorhinidae
AS
AT

Fig. S25. Relative skull height mapped on majority rule consensus tree constrained as **topology 2**. **Abbreviations: AT, Amniotes Total Group; AS, Amniotes Stem Group; AC, Amniotes + Captorhinidae; A, Amniotes.**

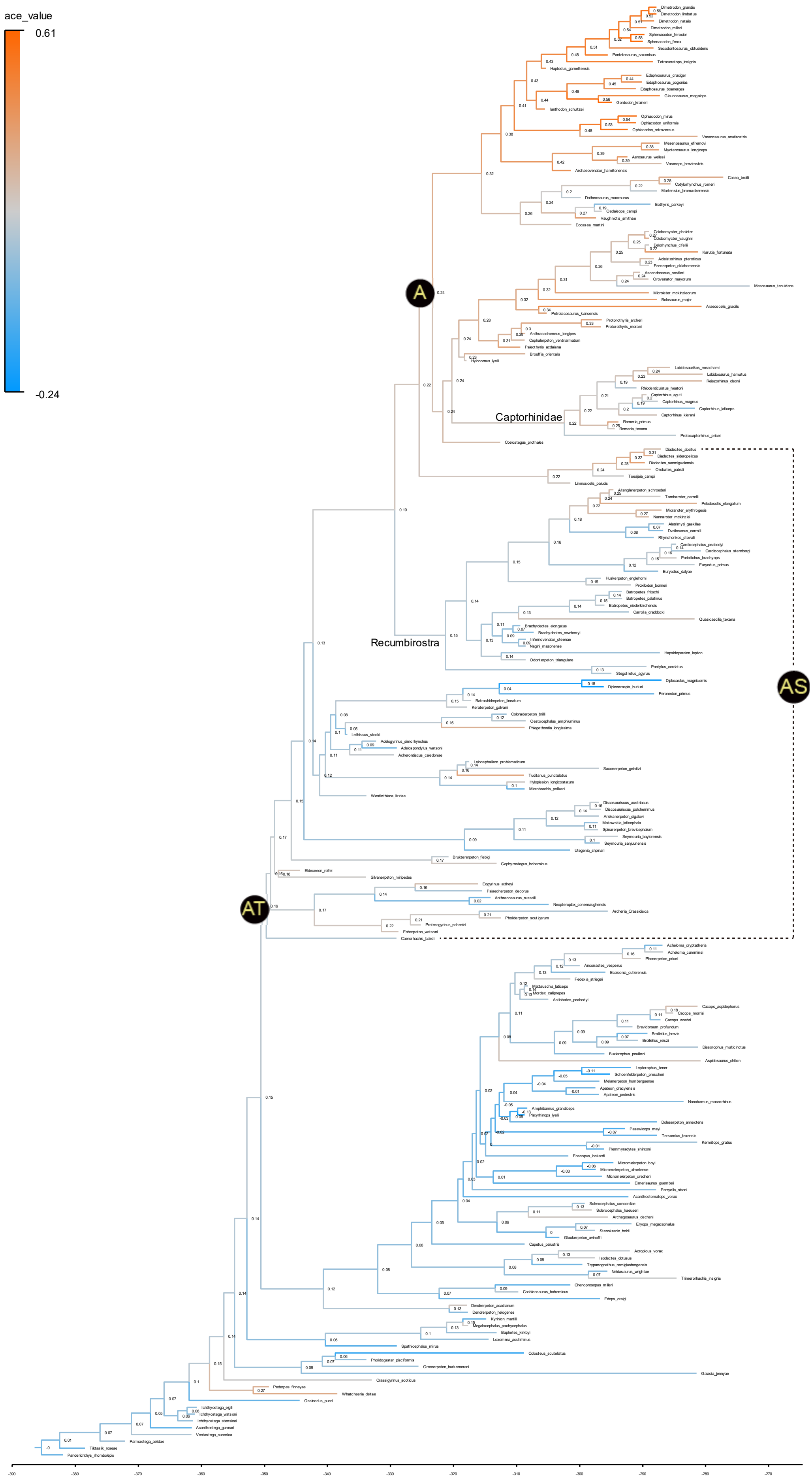
ace_value
0.61
-0.24
A
Captorhinidae
Recumbirostra
AS
AT

Fig. S26. Relative skull height mapped on majority rule consensus tree constrained as **topology 3**. **Abbreviations: AT, Amniotes Total Group; AS, Amniotes Stem Group; A, Amniotes.**

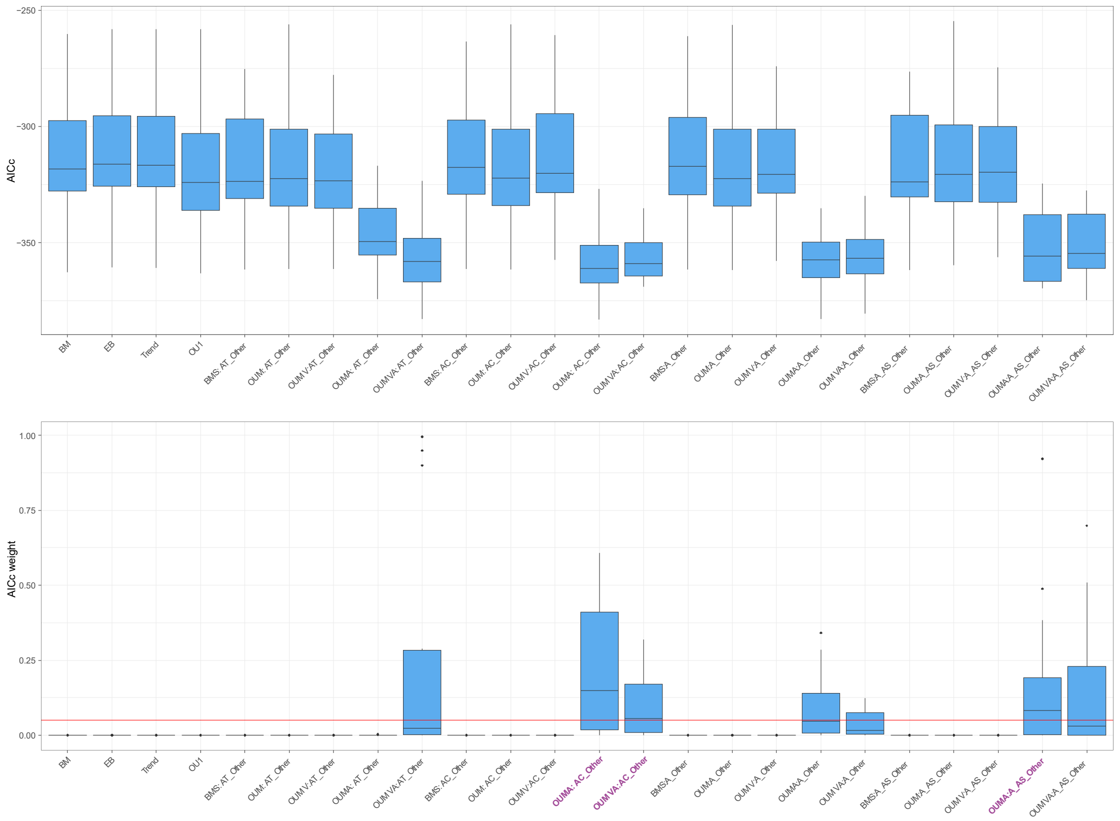

Fig. S27. AICc weight and AICc value from uniform models and multi-regime models, fitted with relative skull height and dated trees constrained as **topology 1**. Red dashed line occurs at 0.05 of AICc weight. **Abbreviations: as Fig S25.**

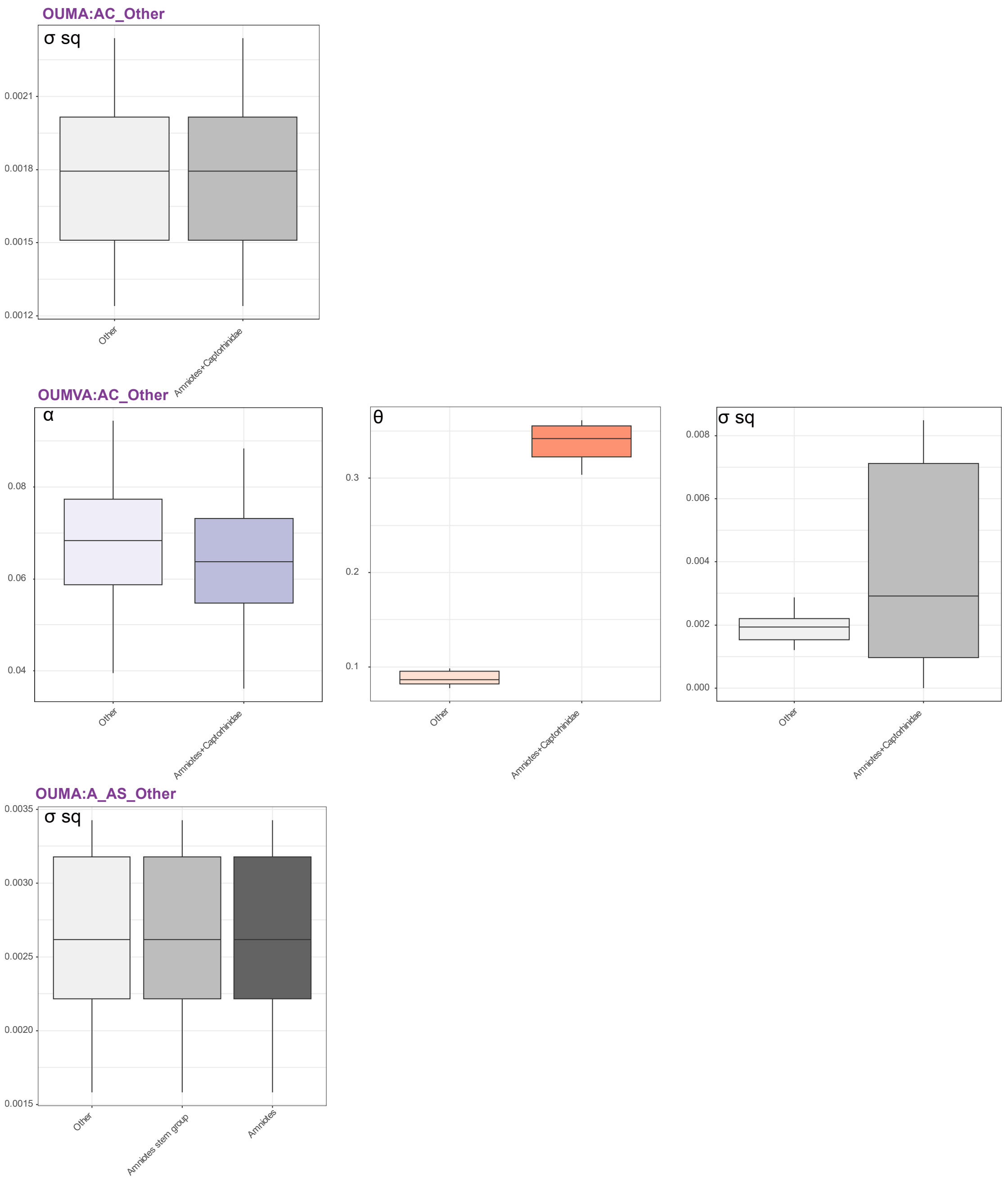

Fig. S28. Parameters estimated from non-negligible models (two-regime OUMA and OUMVA with regime-shift occurred at Amniotes + Captorhinidae; three-regime OUMA model with regime-shifts occurred at both amniotes total group and amniotes), fitted with relative skull height, using dated trees constrained as **topology 1**. **Abbreviations: as Fig S25.**

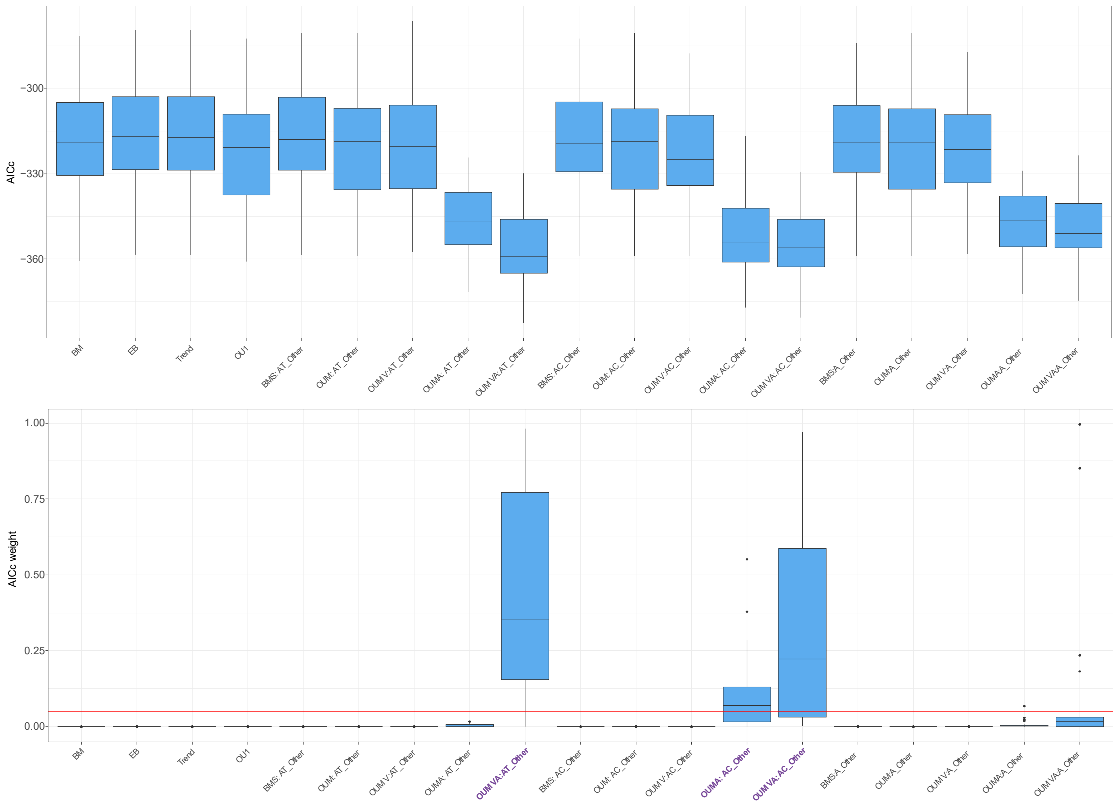

Fig. S29. AICc weight and AICc value from uniform models and multi-regime models, fitted with relative skull height and dated trees constrained as **topology 2**. Red dashed line occurs at 0.05 of AICc weight. **Abbreviations: as Fig S25.**

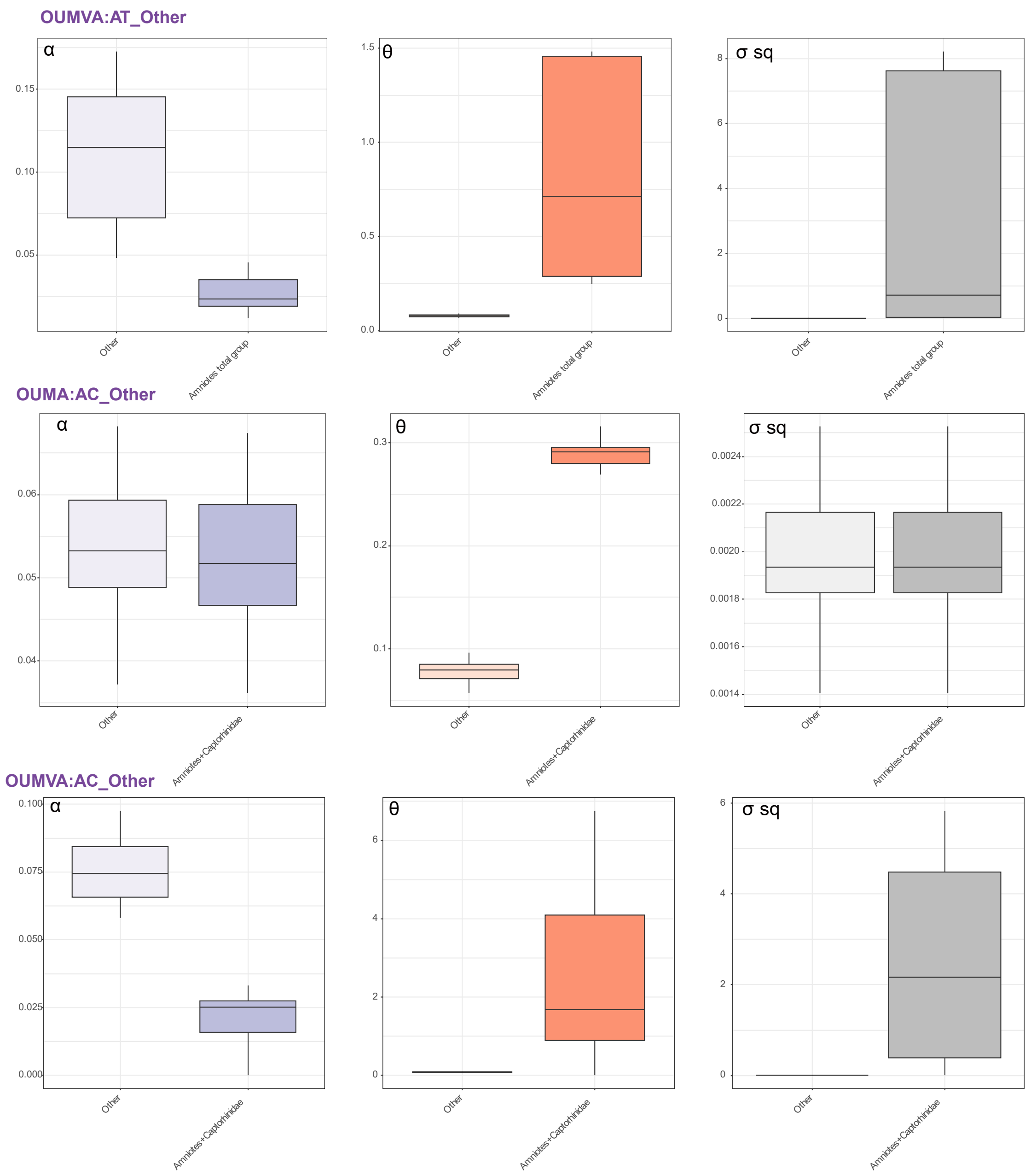

Fig. S30. Parameters estimated from non-negligible models (two-regime OUMVA with regime-shift occurred at Amniotes total group or Amniotes + Captorhinidae, and two-regime OUMA with regime-shift occurred at Amniotes + Captorhinidae), fitted with relative skull height, using dated trees constrained as **topology 2**. **Abbreviations: as Fig S25.**

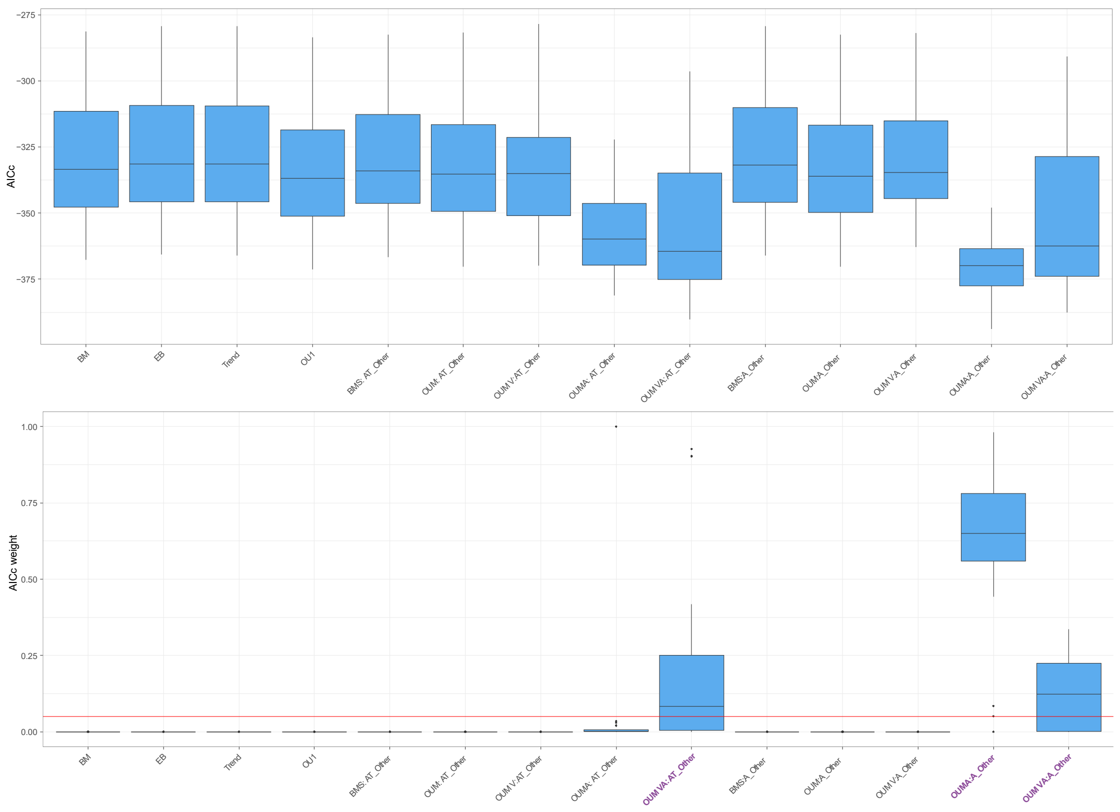

Fig. S31. AICc weight and AICc value from uniform models and multi-regime models, fitted with relative skull height and dated trees constrained as **topology 3**. Red dashed line occurs at 0.05 of AICc weight. **Abbreviations: as Fig S26.**

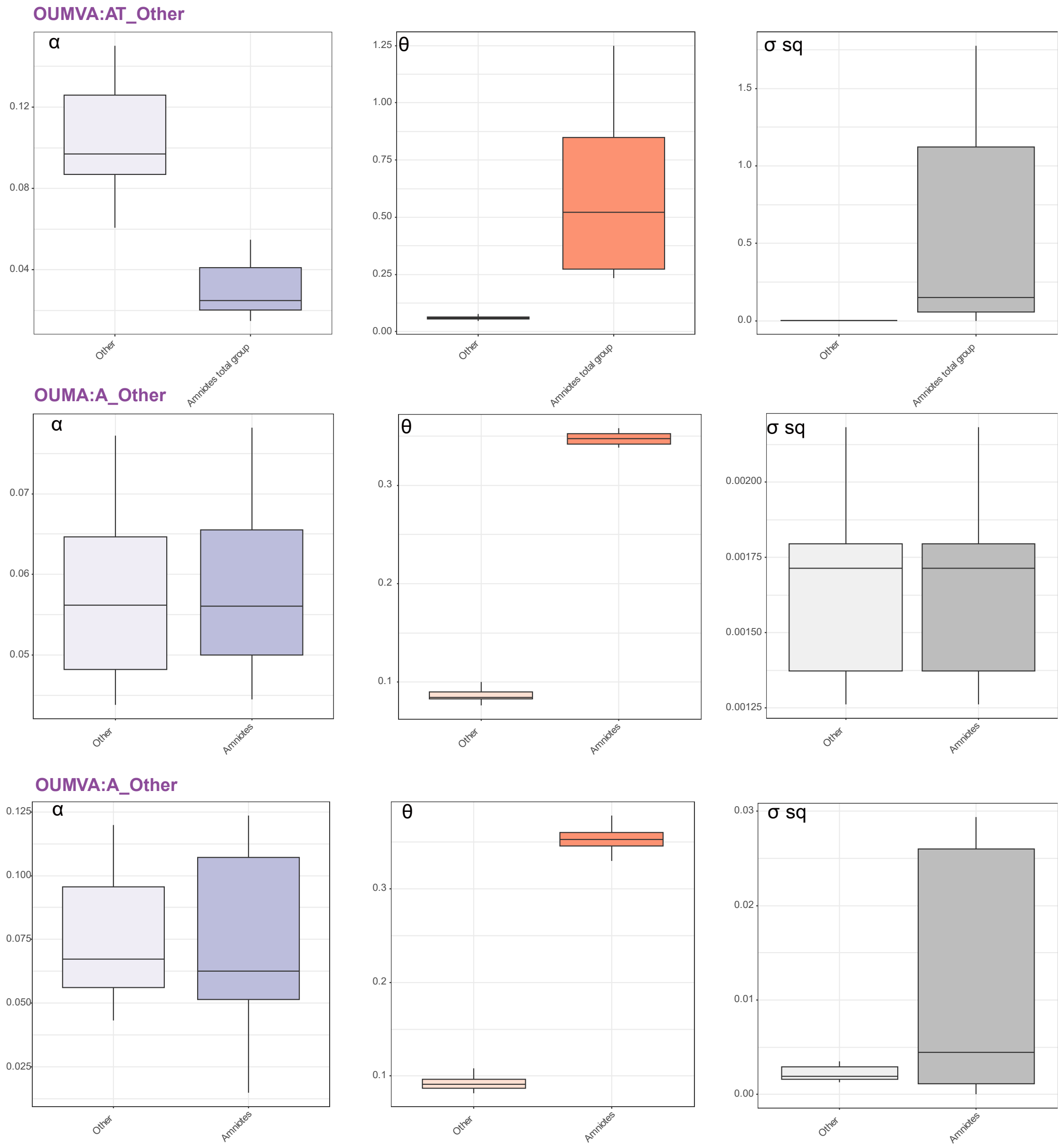

Fig. S32. Parameters estimated from non-negligible models (two-regime OUMA and OUMVA with regime-shift occurred at Amniotes, two-regime OUMVA with regime-shift occurred at amniotes total group), fitted with relative skull height, using dated trees constrained as **topology 3**. **Abbreviations: as Fig S26.**

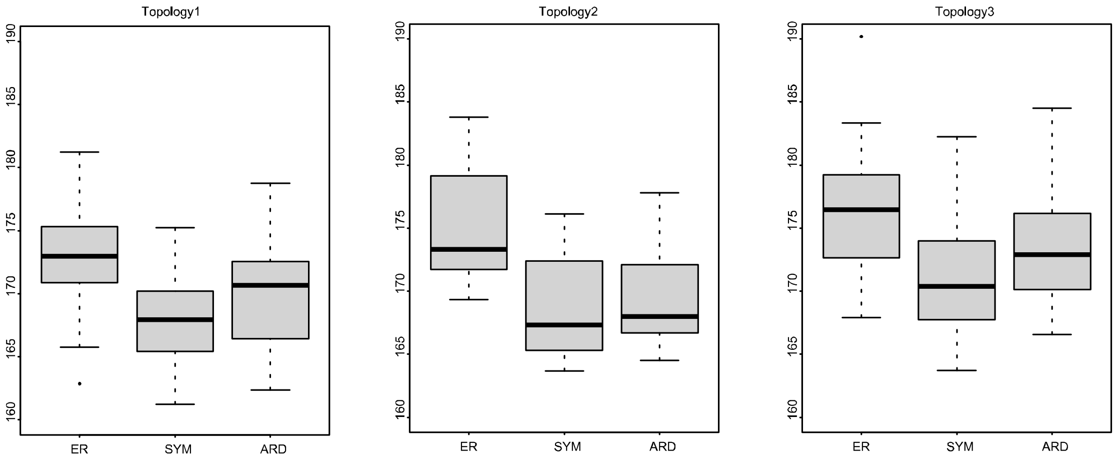

Fig. S33. AICc values of primary ancestral state reconstructions of cervical count with different rate transition matrix. **Abbreviations: ER, equal rate model; SYM, symmetric rate model; ARD, all rate difference model.**

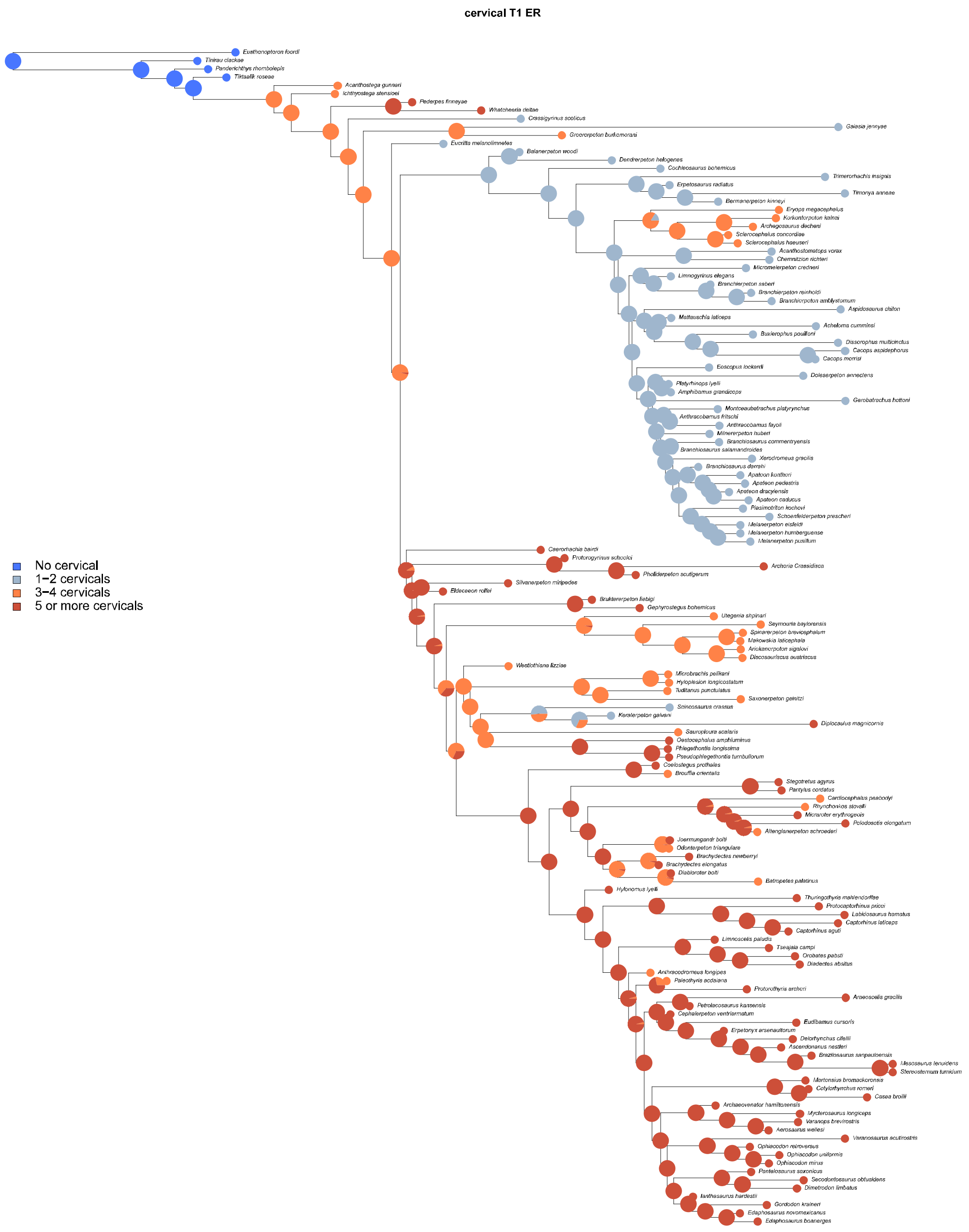

Fig. S34. Summarizing of Cervical count ancestral state reconstruction, using ER model and dated trees constrained as **topology 1**.

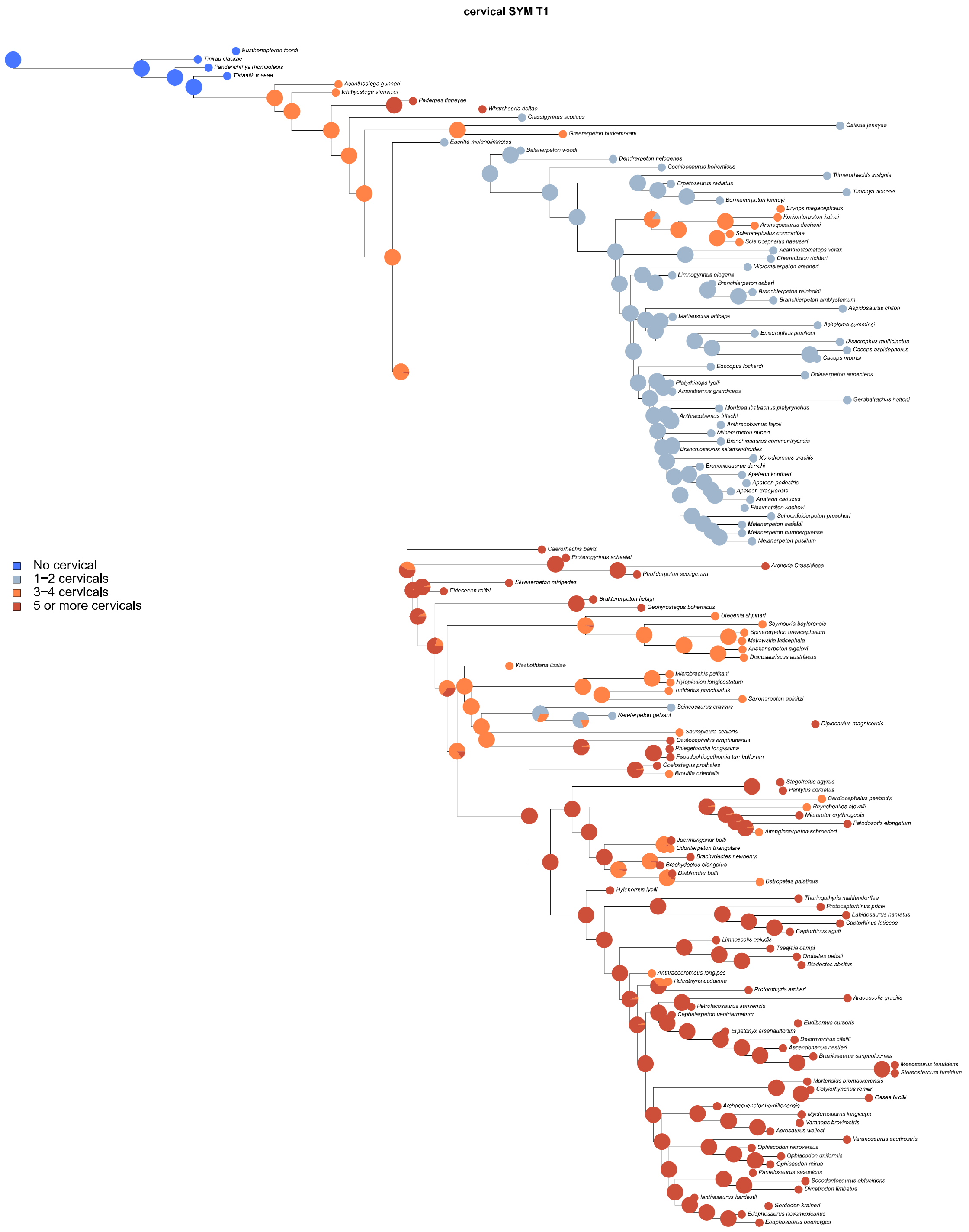

Fig. S35. Summarizing of Cervical count ancestral state reconstruction, using SYM model and dated trees constrained as **topology 1**.

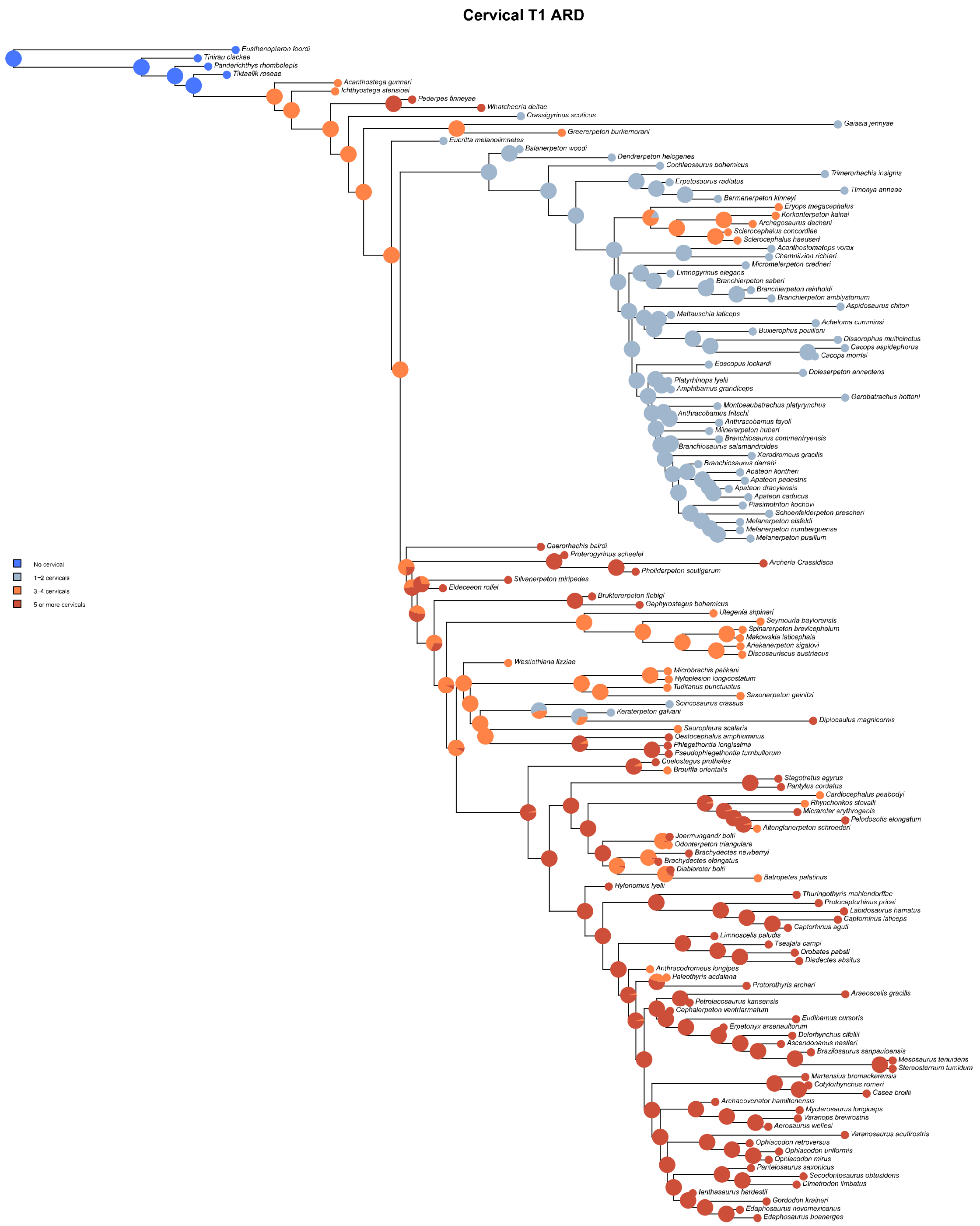

Fig. S36. Summarizing of Cervical count ancestral state reconstruction, using ARD model and dated trees constrained as **topology 1**.

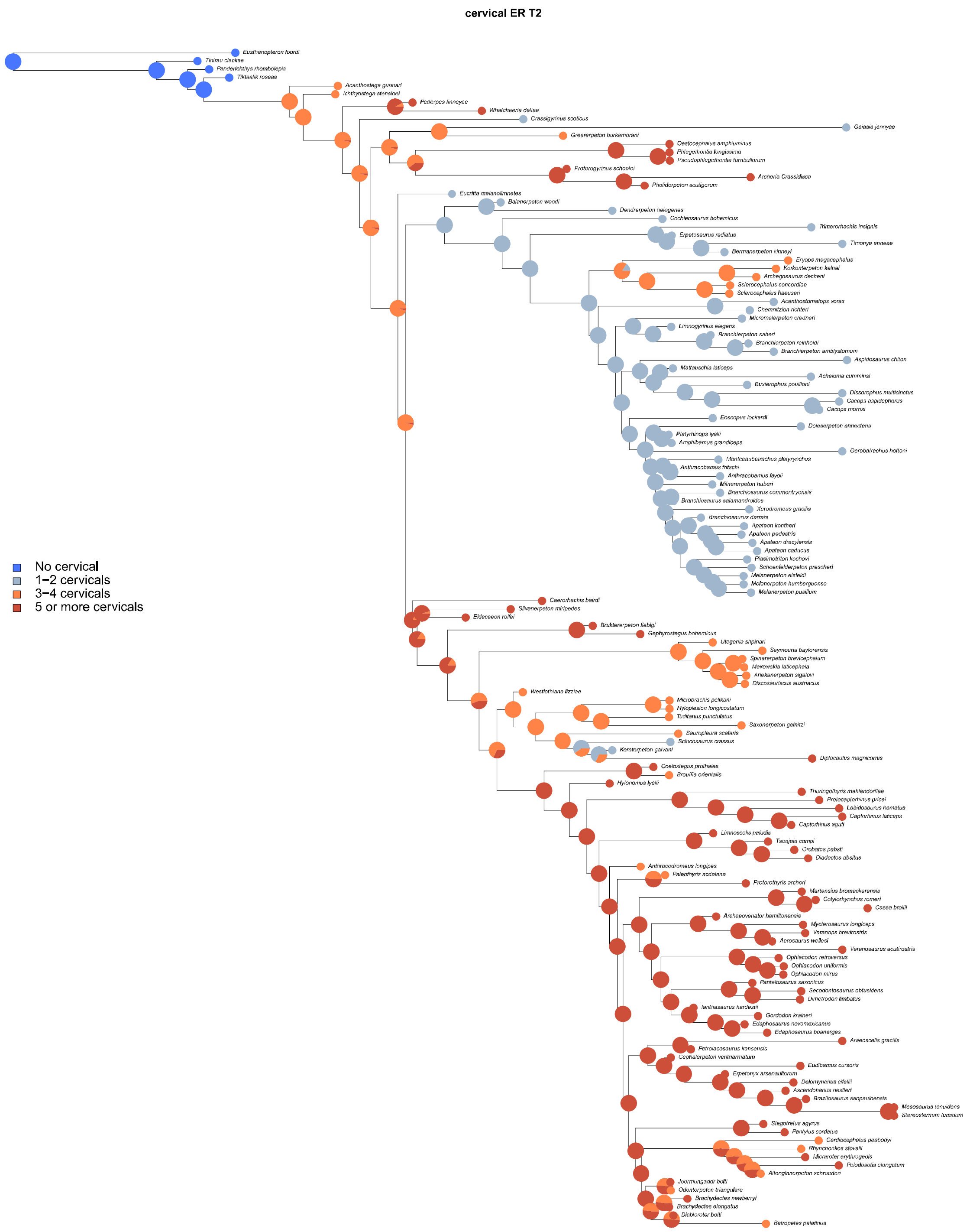

Fig. S37. Summarizing of Cervical count ancestral state reconstruction, using ER model and dated trees constrained as **topology 2**.

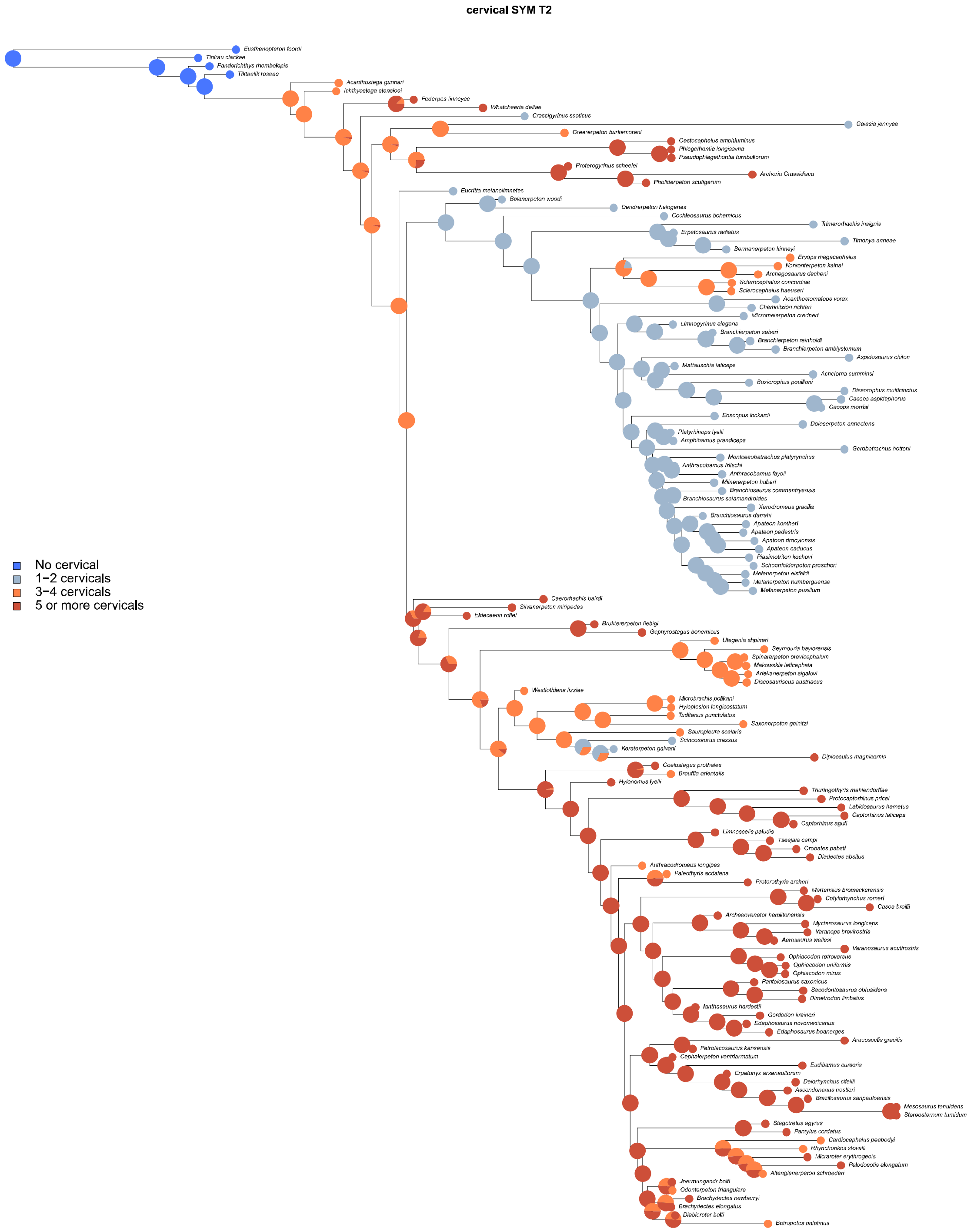

Fig. S38. Summarizing of Cervical count ancestral state reconstruction, using SYM model and dated trees constrained as **topology 2**.

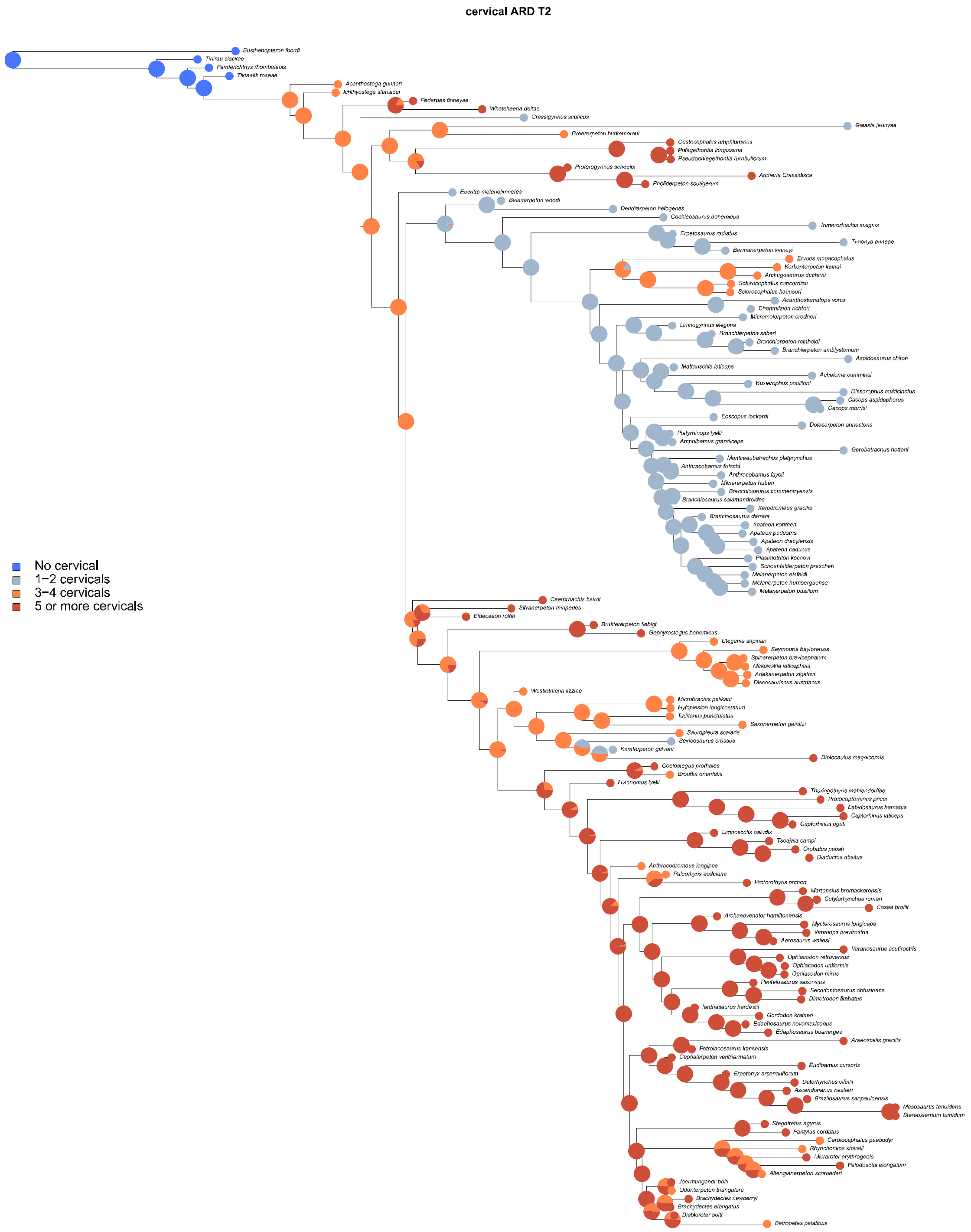

Fig. S39. Summarizing of Cervical count ancestral state reconstruction, using ARD model and dated trees constrained as **topology 2**.

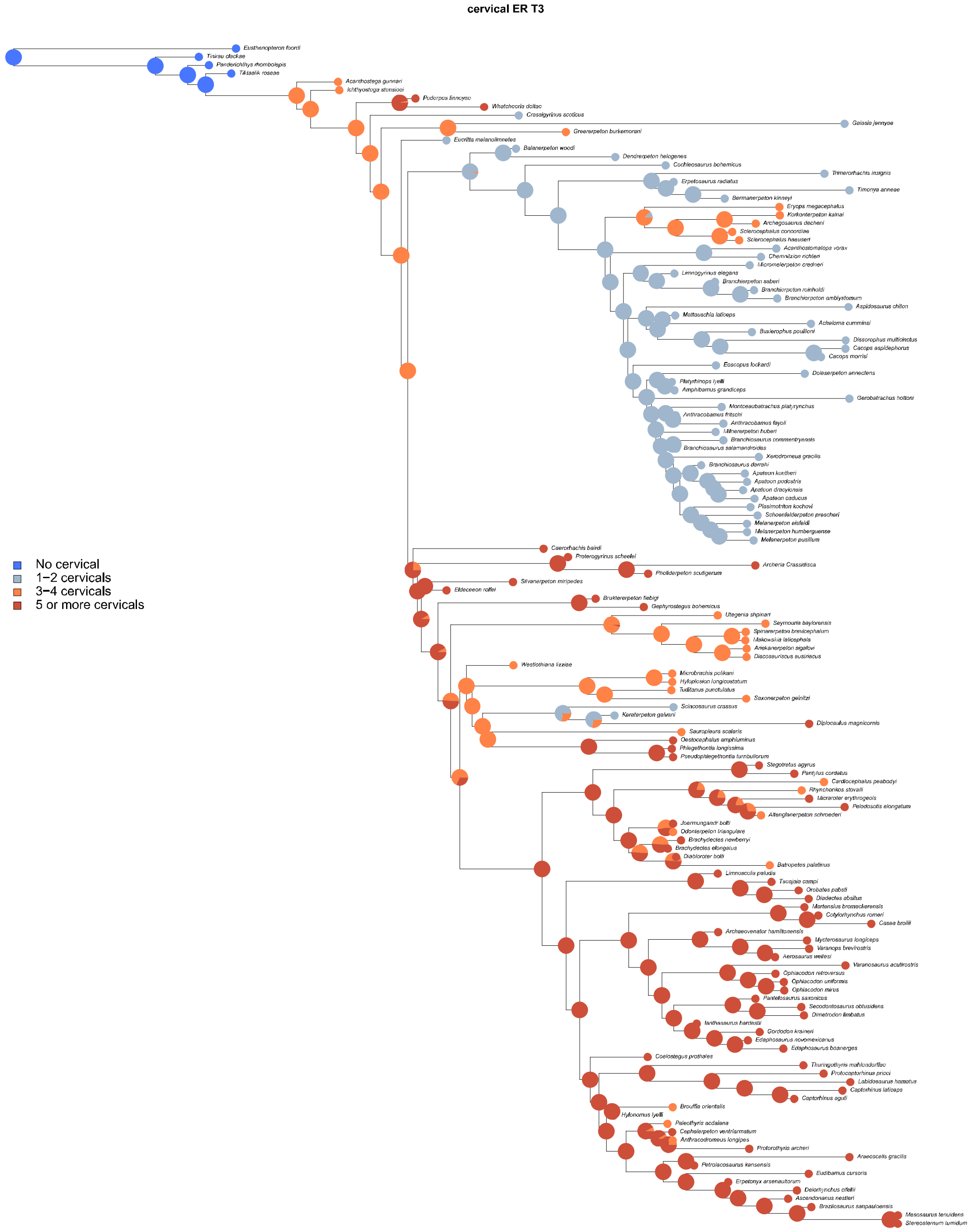

Fig. S40. Summarizing of Cervical count ancestral state reconstruction, using ER model and dated trees constrained as **topology 3**.

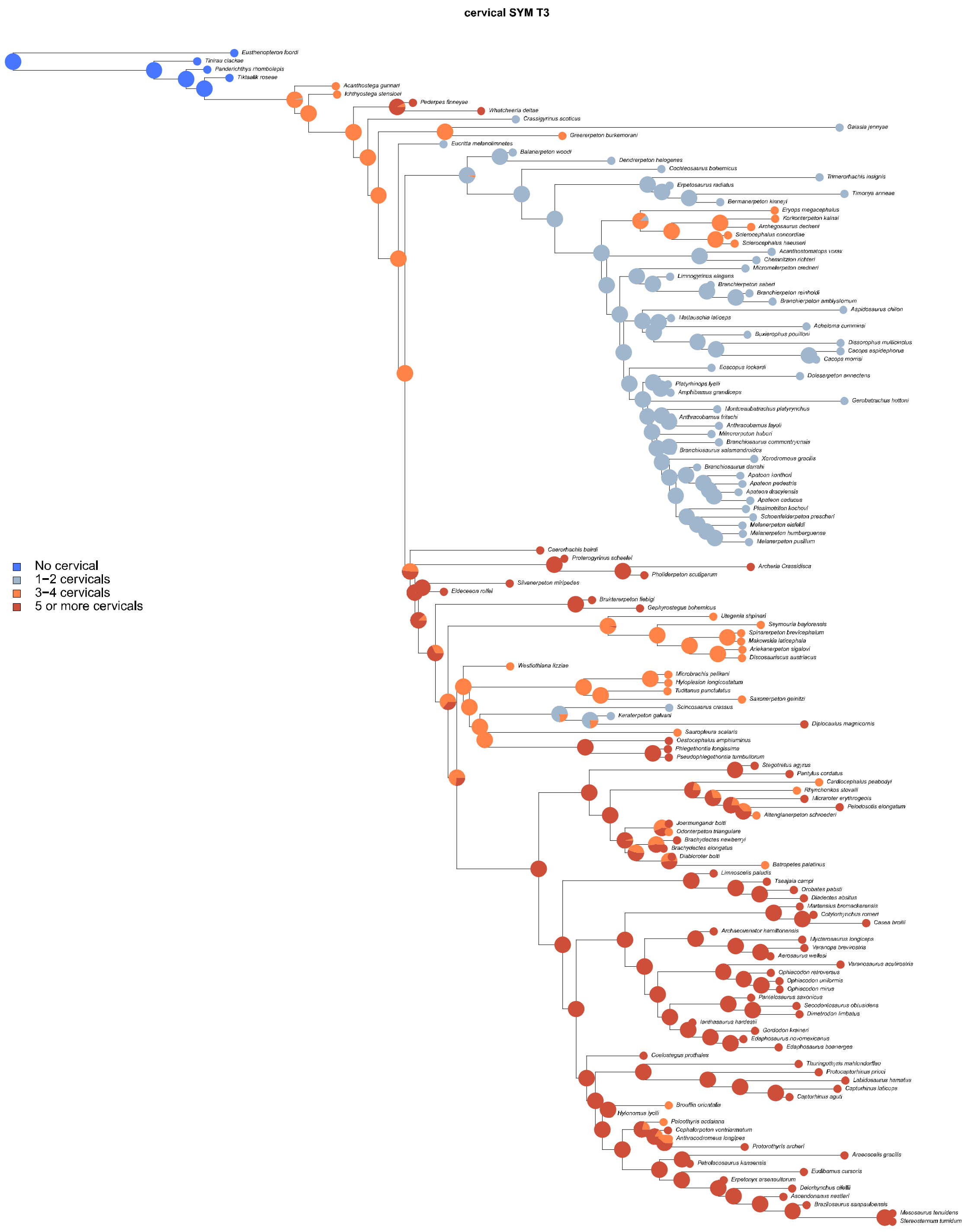

Fig. S41. Summarizing of Cervical count ancestral state reconstruction, using SYM model and dated trees constrained as **topology 3**.

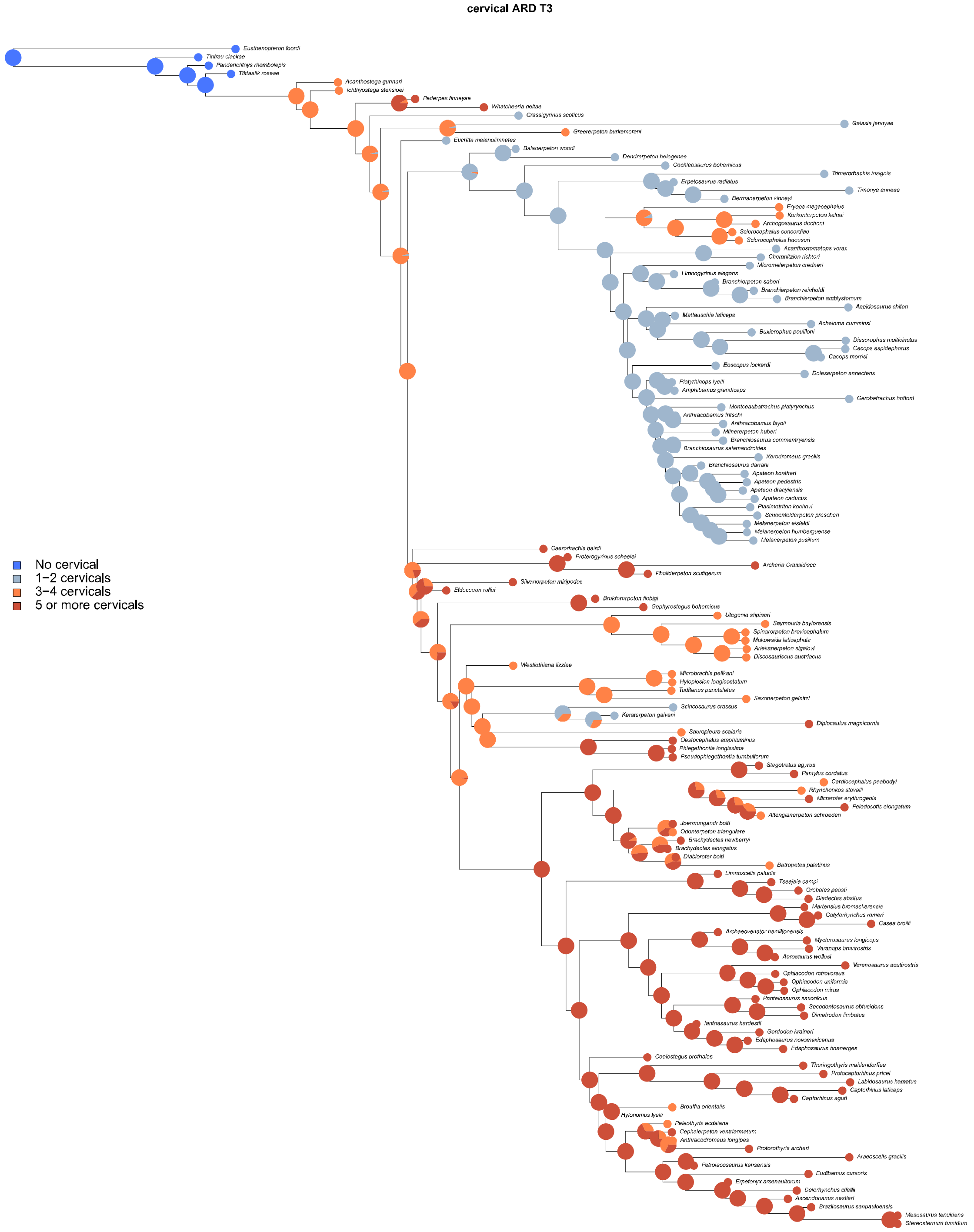

Fig. S42. Summarizing of Cervical count ancestral state reconstruction, using ARD model and dated trees constrained as **topology 3**.

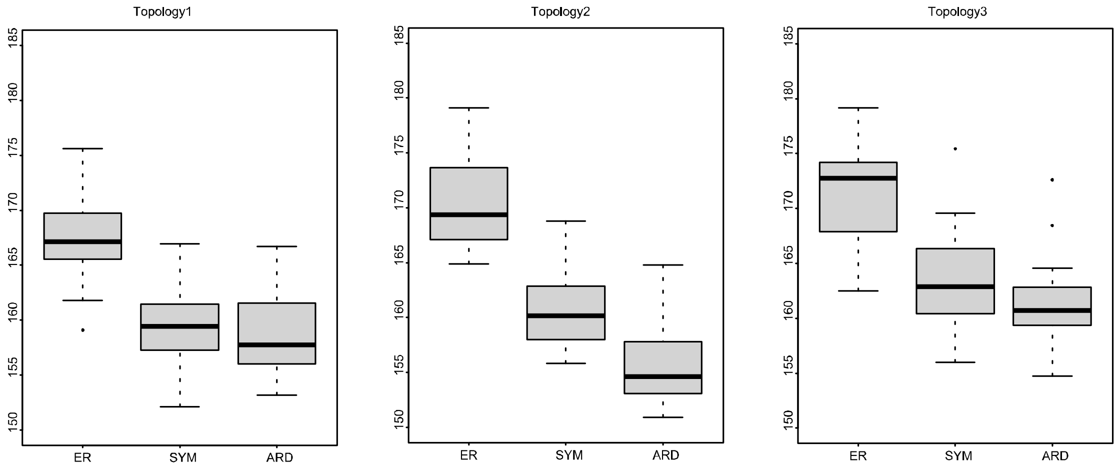

Fig. S43. AICc values of additional ancestral state reconstructions of cervical count **(Temnospondyls are coded as having only two cervical, the atlas and the axis)** with different rate transition matrix. Abbreviations: ER, equal rate model; SYM, symmetric rate model; ARD, all rate difference model.

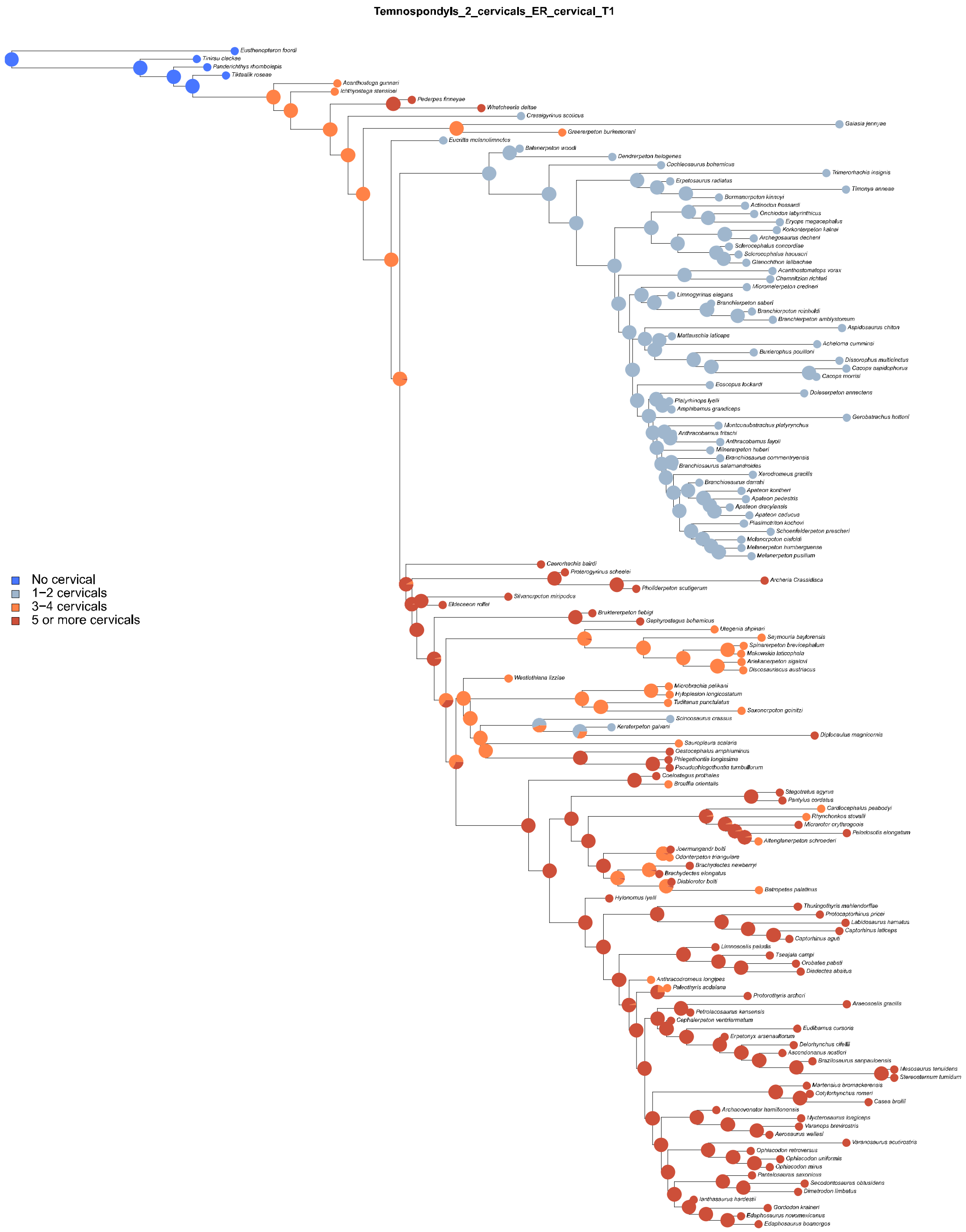

Fig. S44. Summarizing of Cervical count ancestral state reconstruction **(Temnospondyls are coded as having only two cervical, the atlas and the axis)**, using ER model and dated trees constrained as **topology 1**.

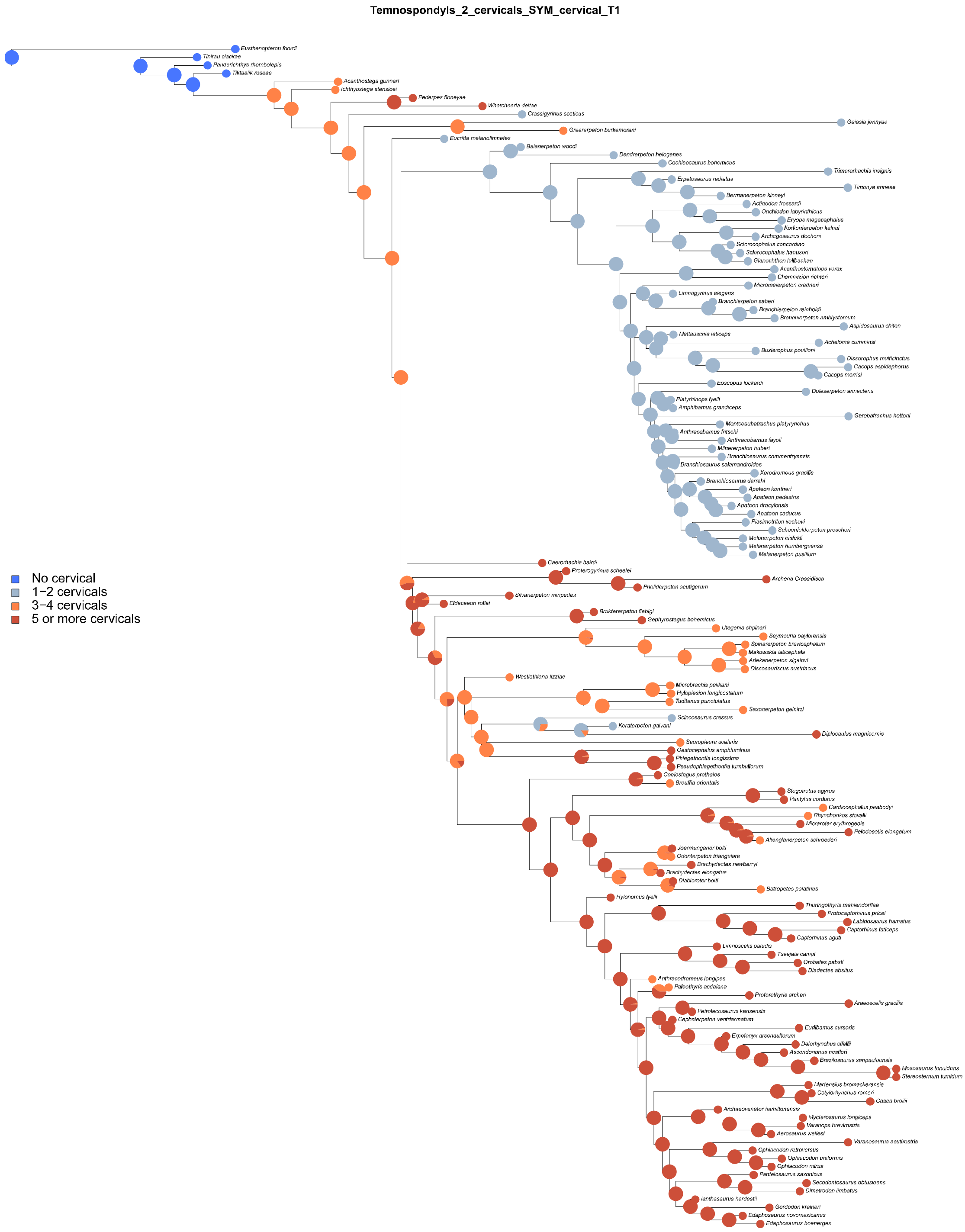

Fig. S45. Summarizing of Cervical count ancestral state reconstruction **(Temnospondyls are coded as having only two cervical, the atlas and the axis)**, using SYM model and dated trees constrained as **topology 1**.

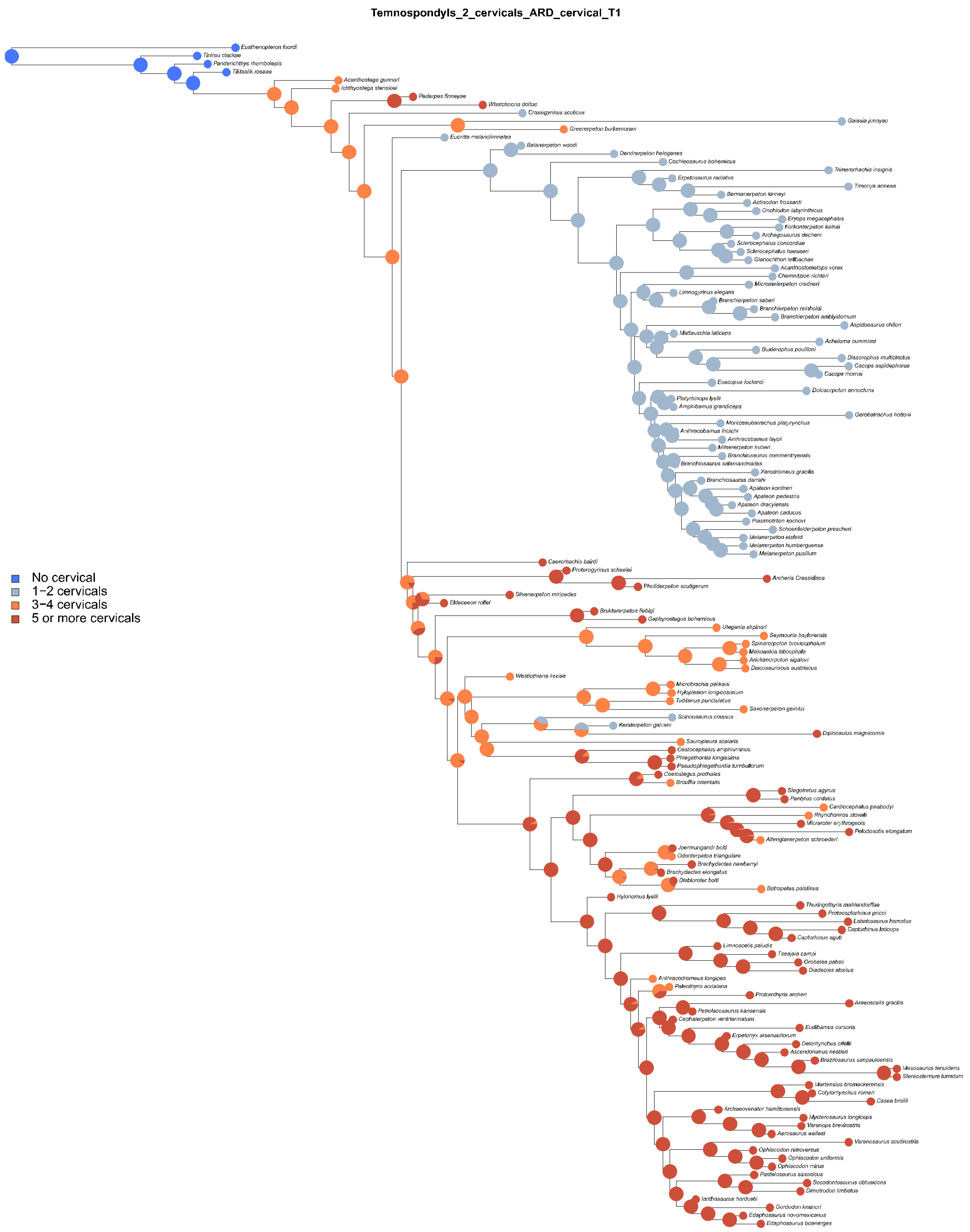

Fig. S46. Summarizing of Cervical count ancestral state reconstruction **(Temnospondyls are coded as having only two cervical, the atlas and the axis)**, using ARD model and dated trees constrained as **topology 1**.

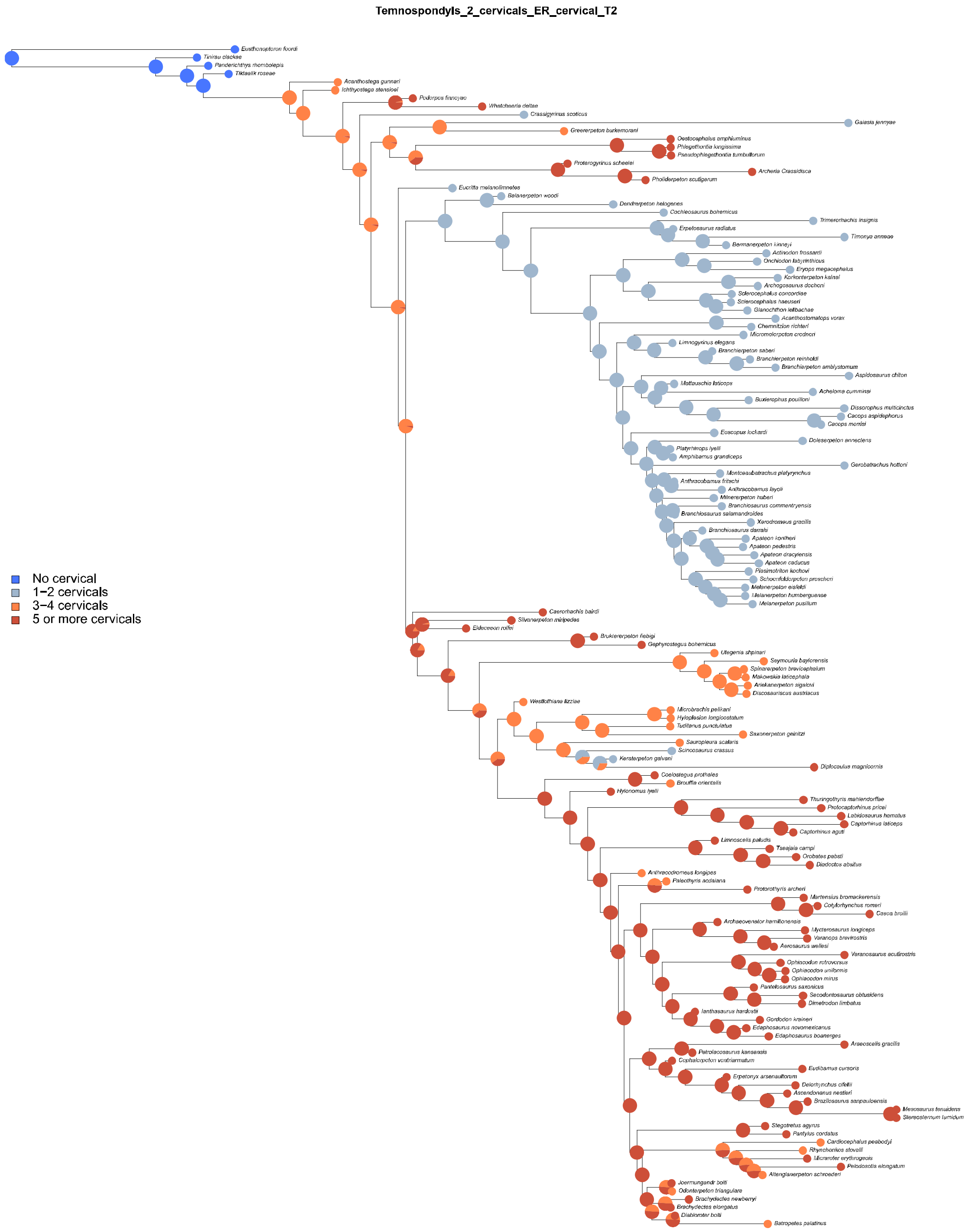

Fig. S47. Summarizing of Cervical count ancestral state reconstruction **(Temnospondyls are coded as having only two cervical, the atlas and the axis)**, using ER model and dated trees constrained as **topology 2**.

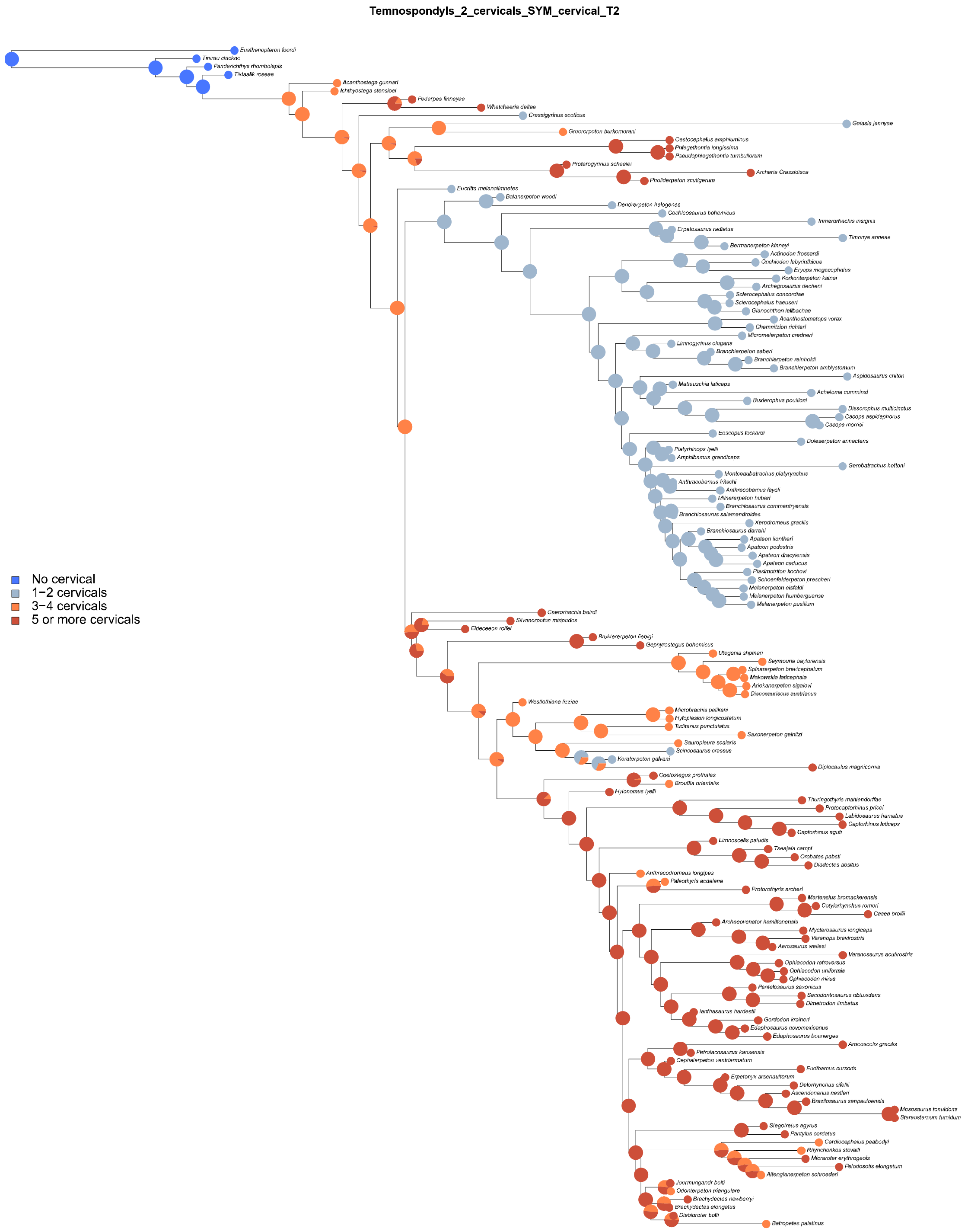

Fig. S48. Summarizing of Cervical count ancestral state reconstruction **(Temnospondyls are coded as having only two cervical, the atlas and the axis)**, using SYM model and dated trees constrained as **topology 2**.

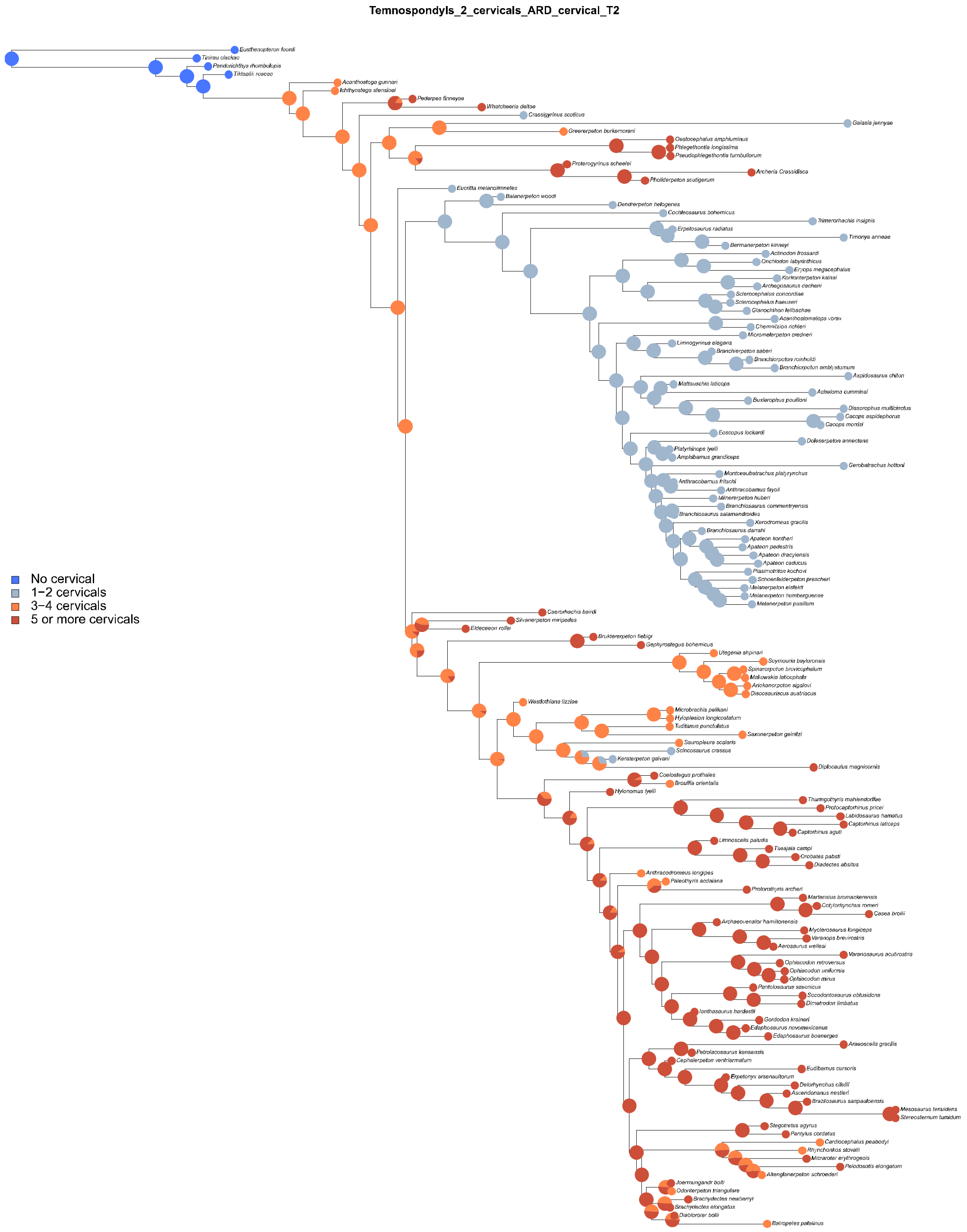

Fig. S49. Summarizing of Cervical count ancestral state reconstruction **(Temnospondyls are coded as having only two cervical, the atlas and the axis)**, using ARD model and dated trees constrained as **topology 2**.

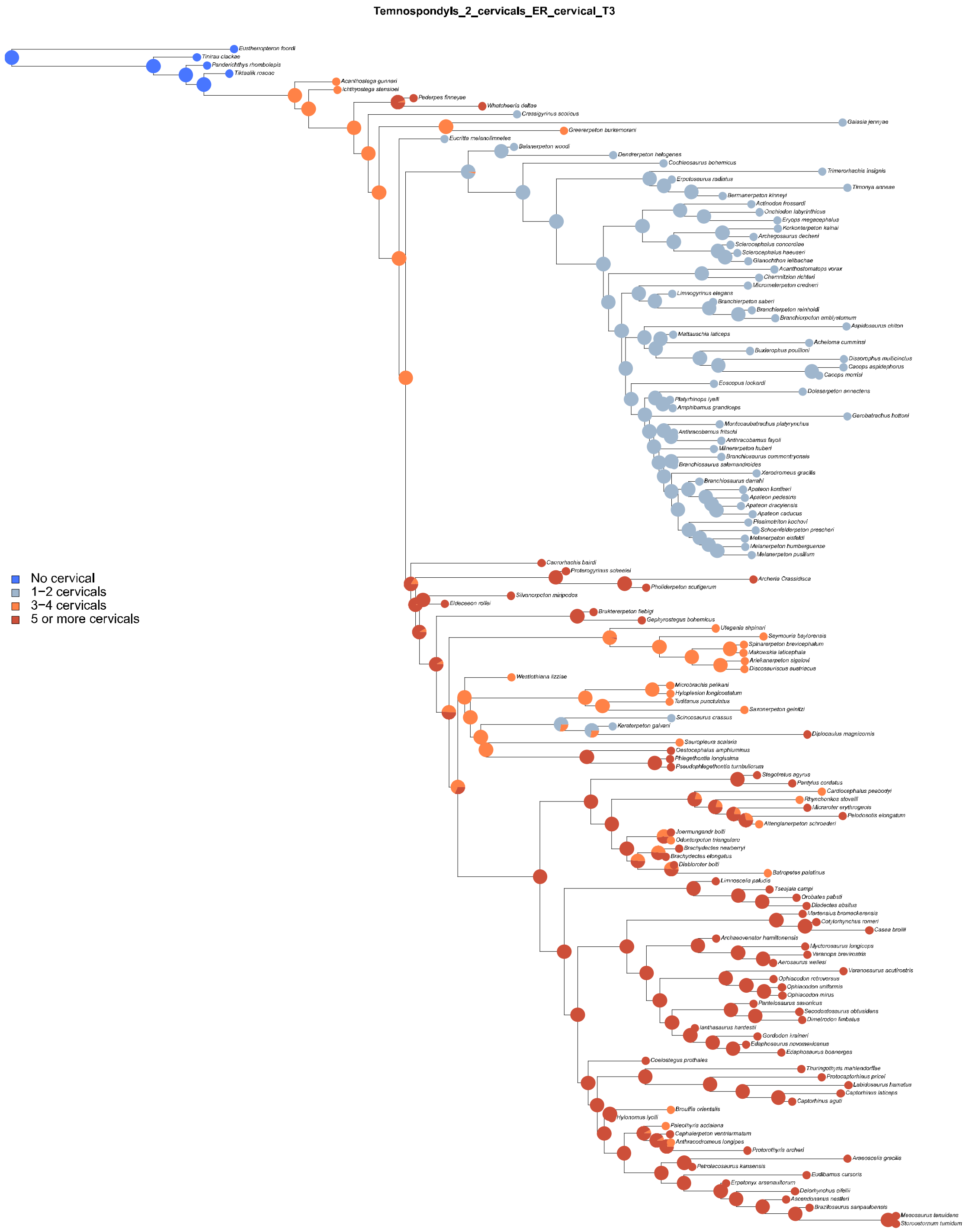

Fig. S50. Summarizing of Cervical count ancestral state reconstruction **(Temnospondyls are coded as having only two cervical, the atlas and the axis)**, using ER model and dated trees constrained as **topology 3**.

Temnospondyls_2_cervicals_SYM_cervical_T3

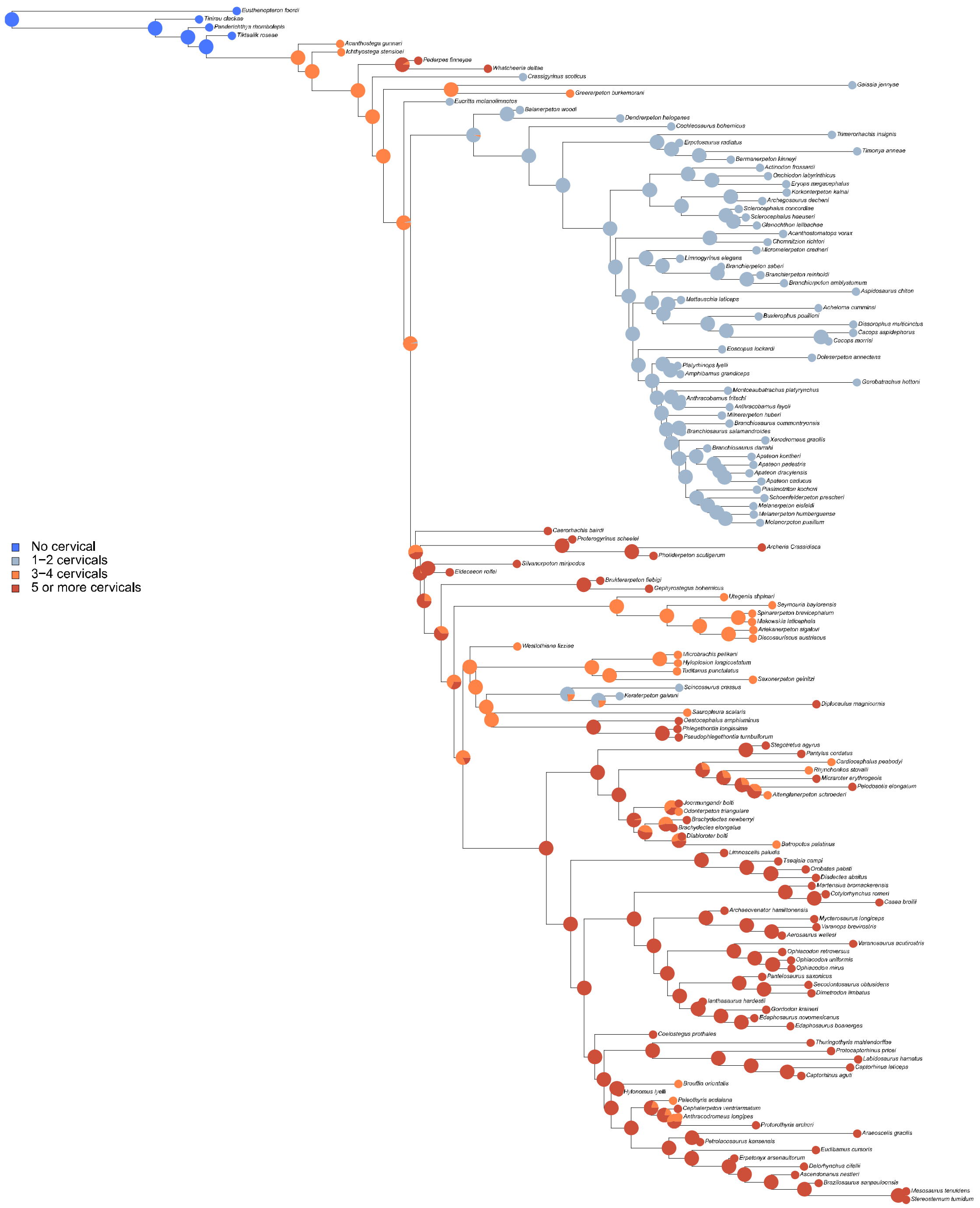

Fig. S51. Summarizing of Cervical count ancestral state reconstruction **(Temnospondyls are coded as having only two cervical, the atlas and the axis)**, using SYM model and dated trees constrained as **topology 3**.

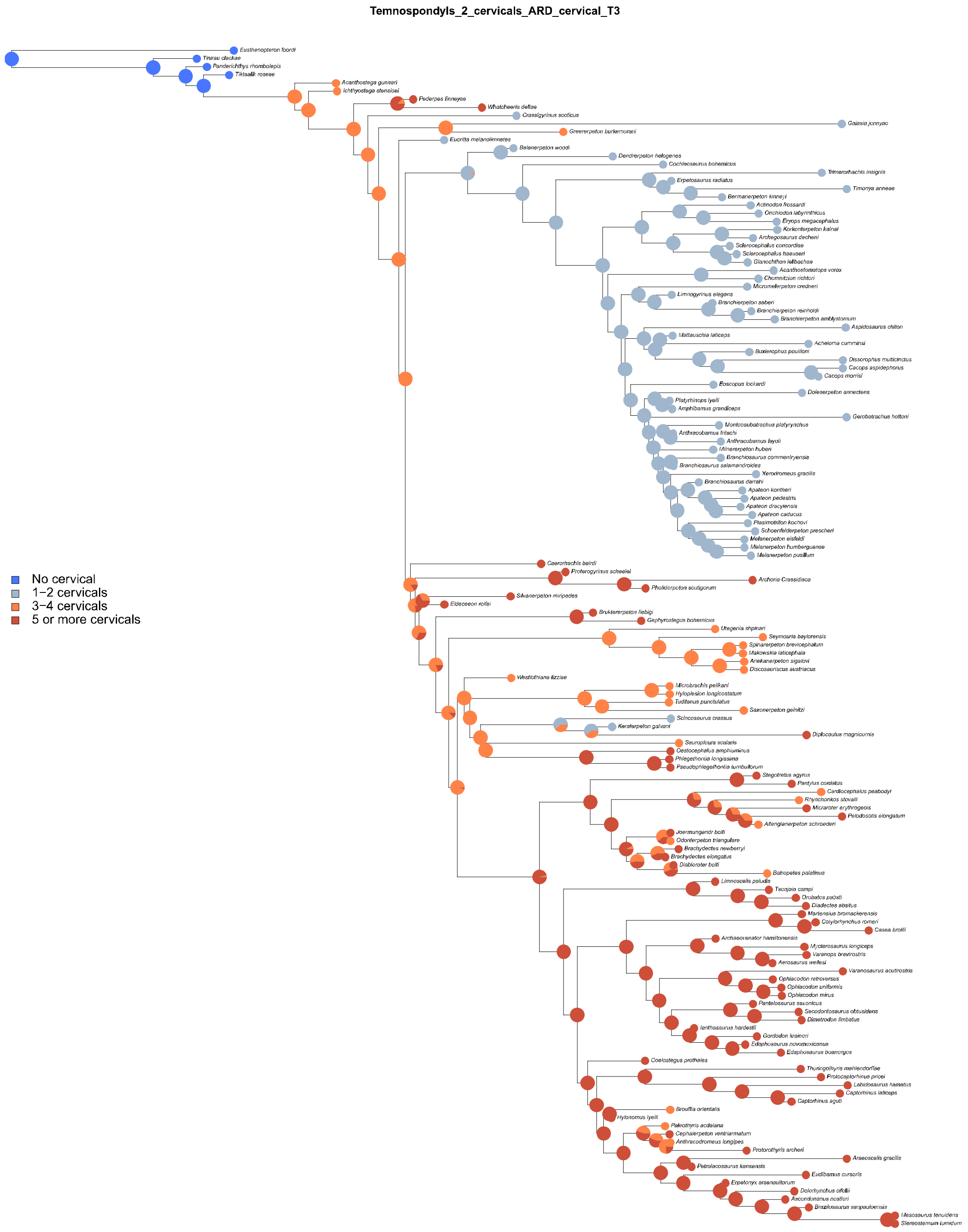

Fig. S52. Summarizing of Cervical count ancestral state reconstruction **(Temnospondyls are coded as having only two cervical, the atlas and the axis)**, using ARD model and dated trees constrained as **topology 3**.

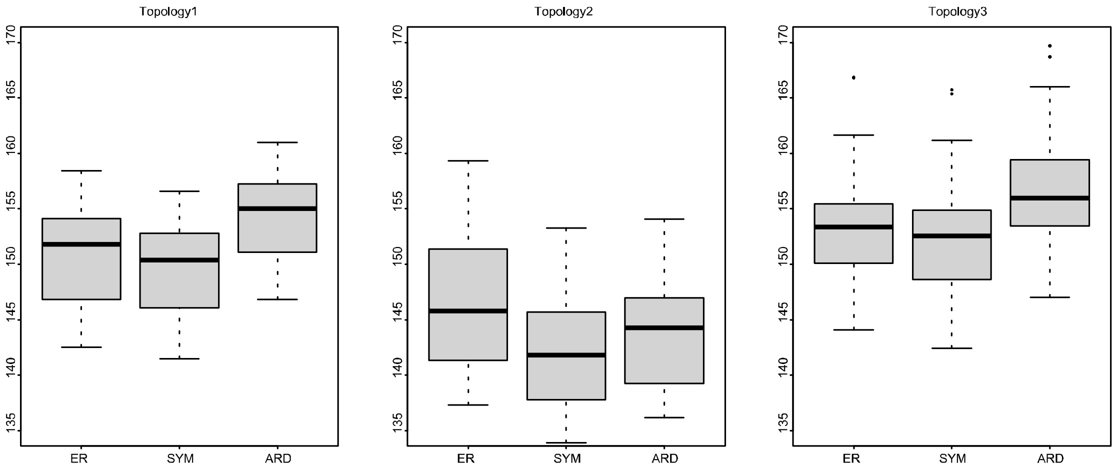

Fig. S53. AICc values of ancestral state reconstructions of trunk rib morphology with different rate transition matrix. **Abbreviations: ER, equal rate model; SYM, symmetric rate model; ARD, all rate difference model.**

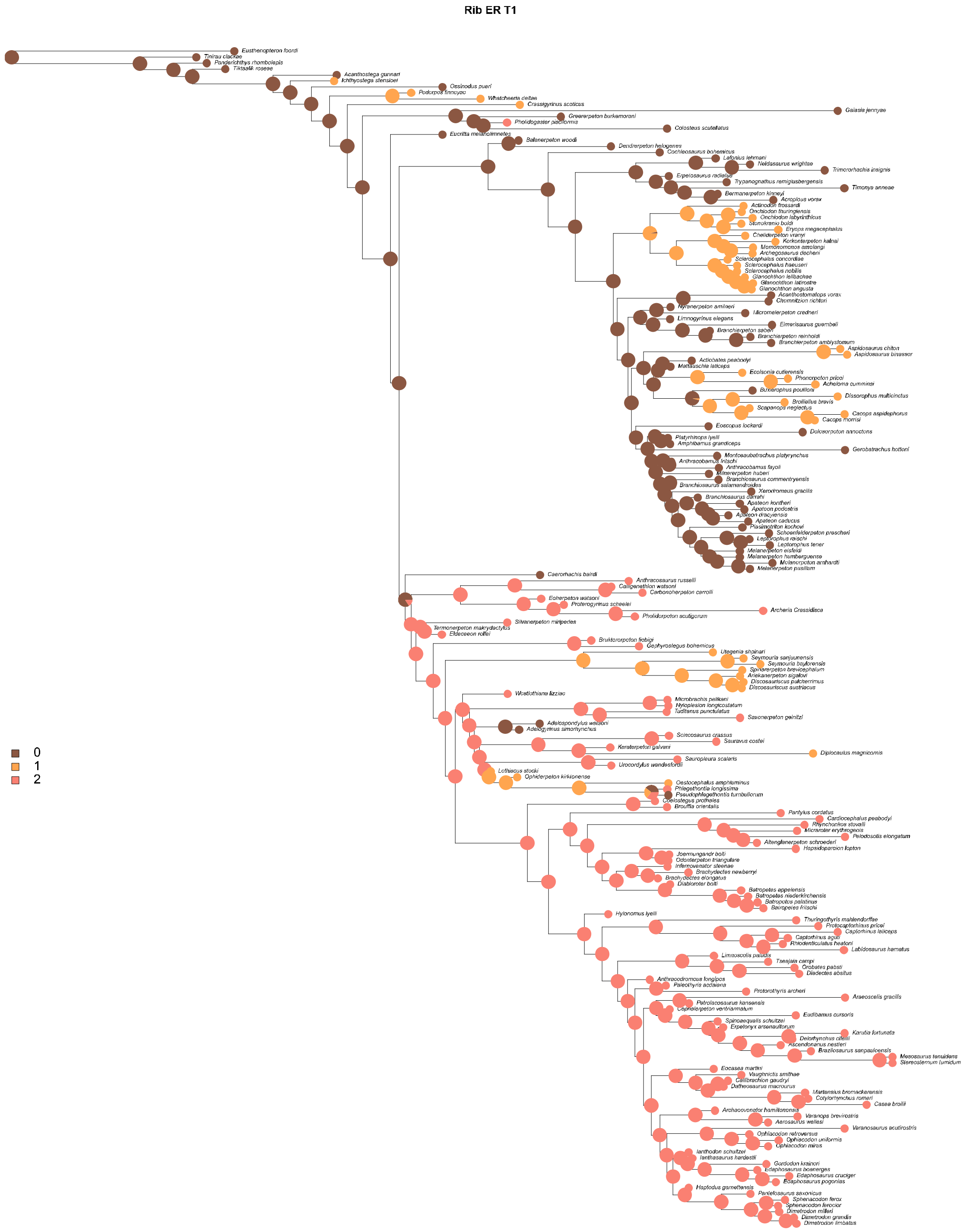

Fig. S54. Summarizing of trunk rib morphology ancestral state reconstruction, using ER model and dated trees constrained as **topology 1**.

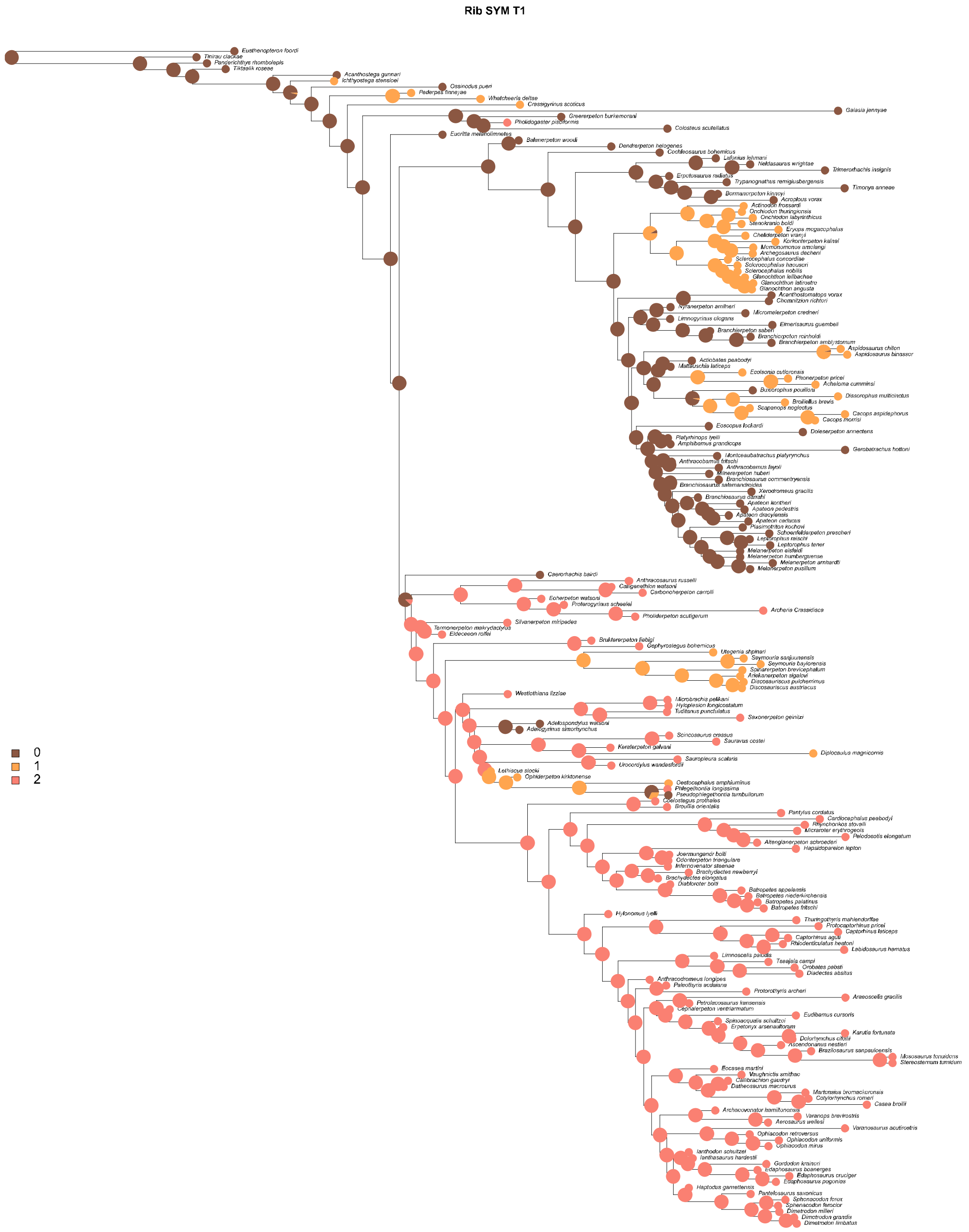

Fig. S55. Summarizing of trunk rib morphology ancestral state reconstruction, using SYM model and dated trees constrained as **topology 1**.

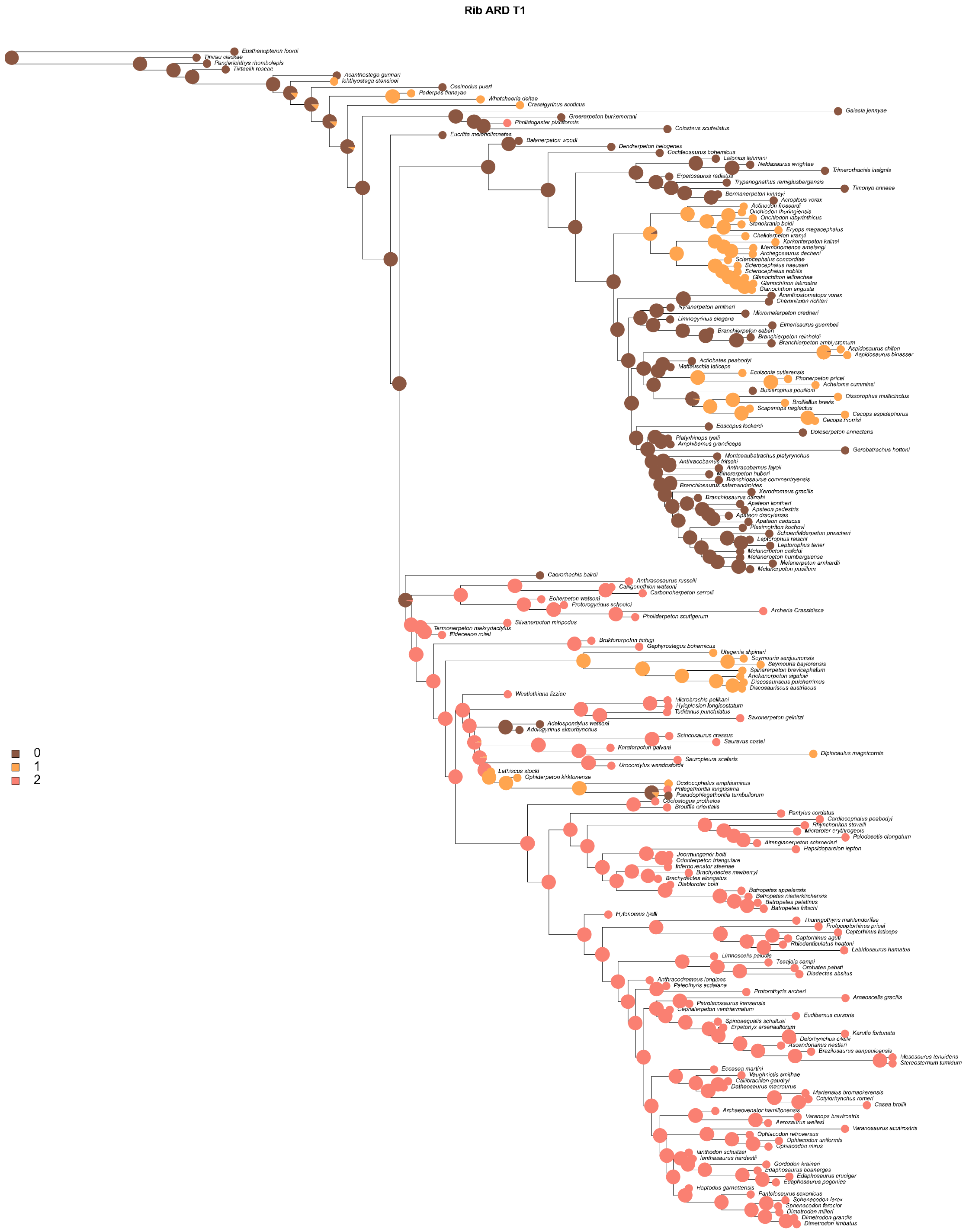

Fig. S56. Summarizing of trunk rib morphology ancestral state reconstruction, using ARD model and dated trees constrained as **topology 1**.

Rib ER T2

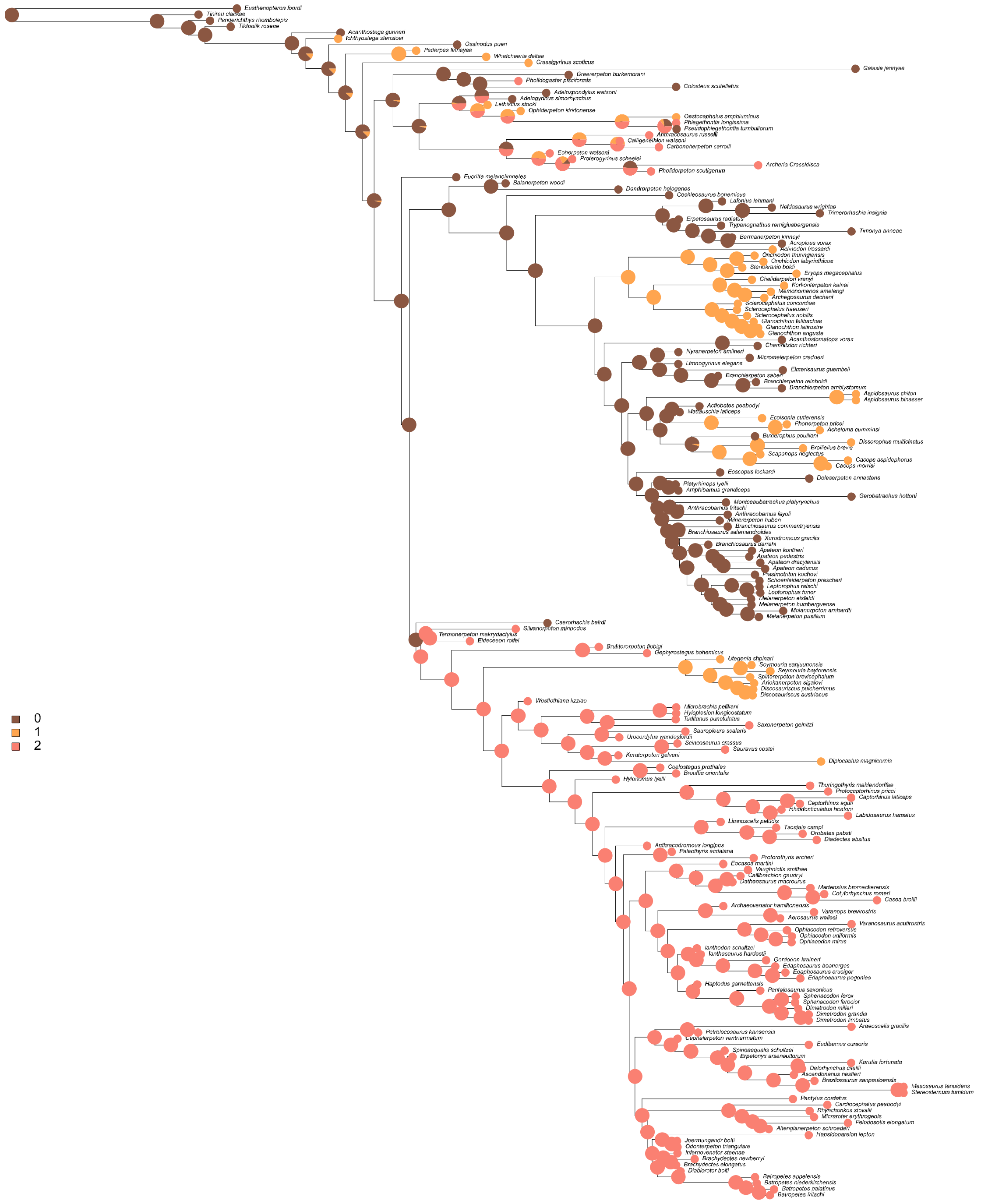

Fig. S57. Summarizing of trunk rib morphology ancestral state reconstruction, using ER model and dated trees constrained as **topology 2**.

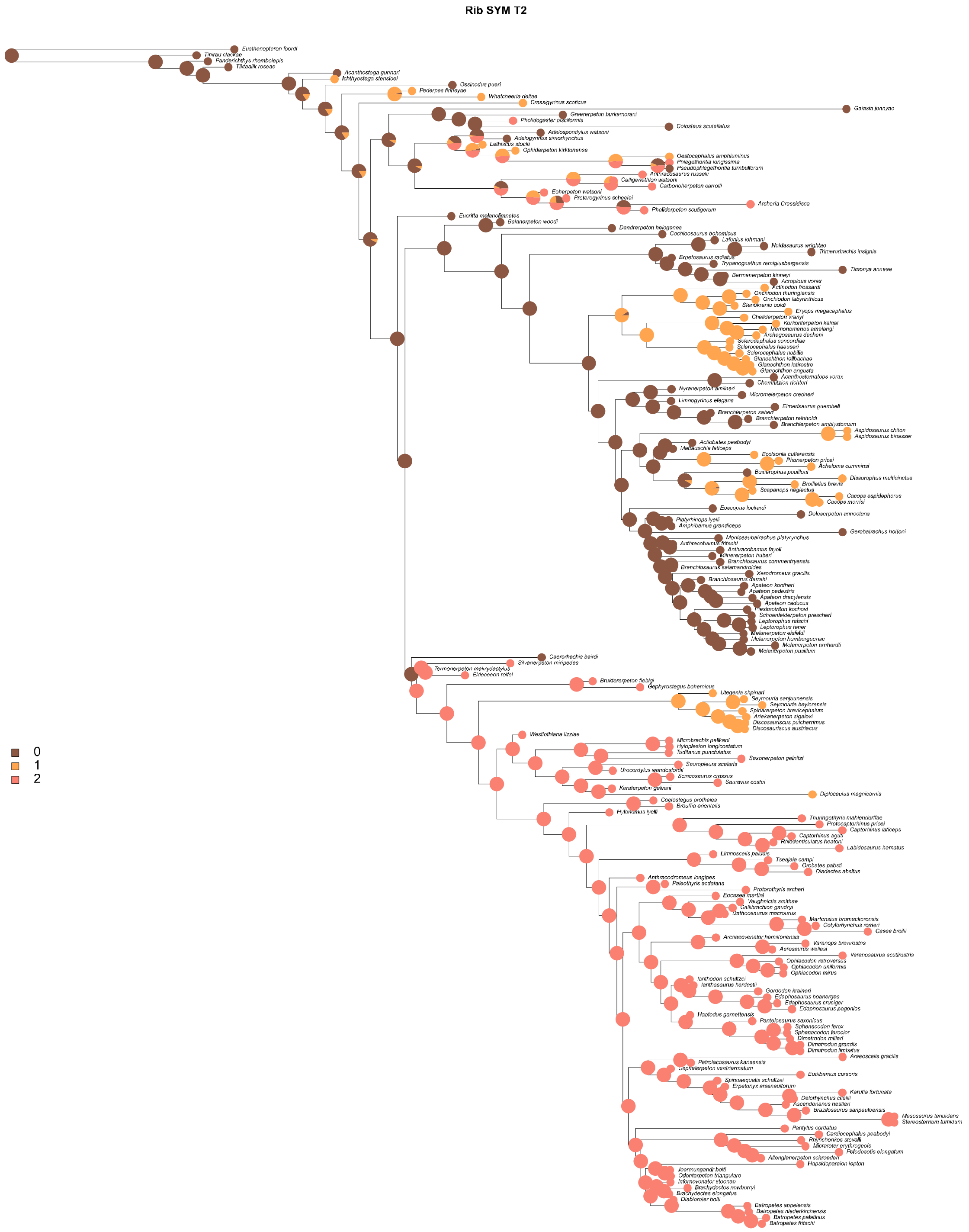

Fig. S58. Summarizing of trunk rib morphology ancestral state reconstruction, using SYM model and dated trees constrained as **topology 2**.

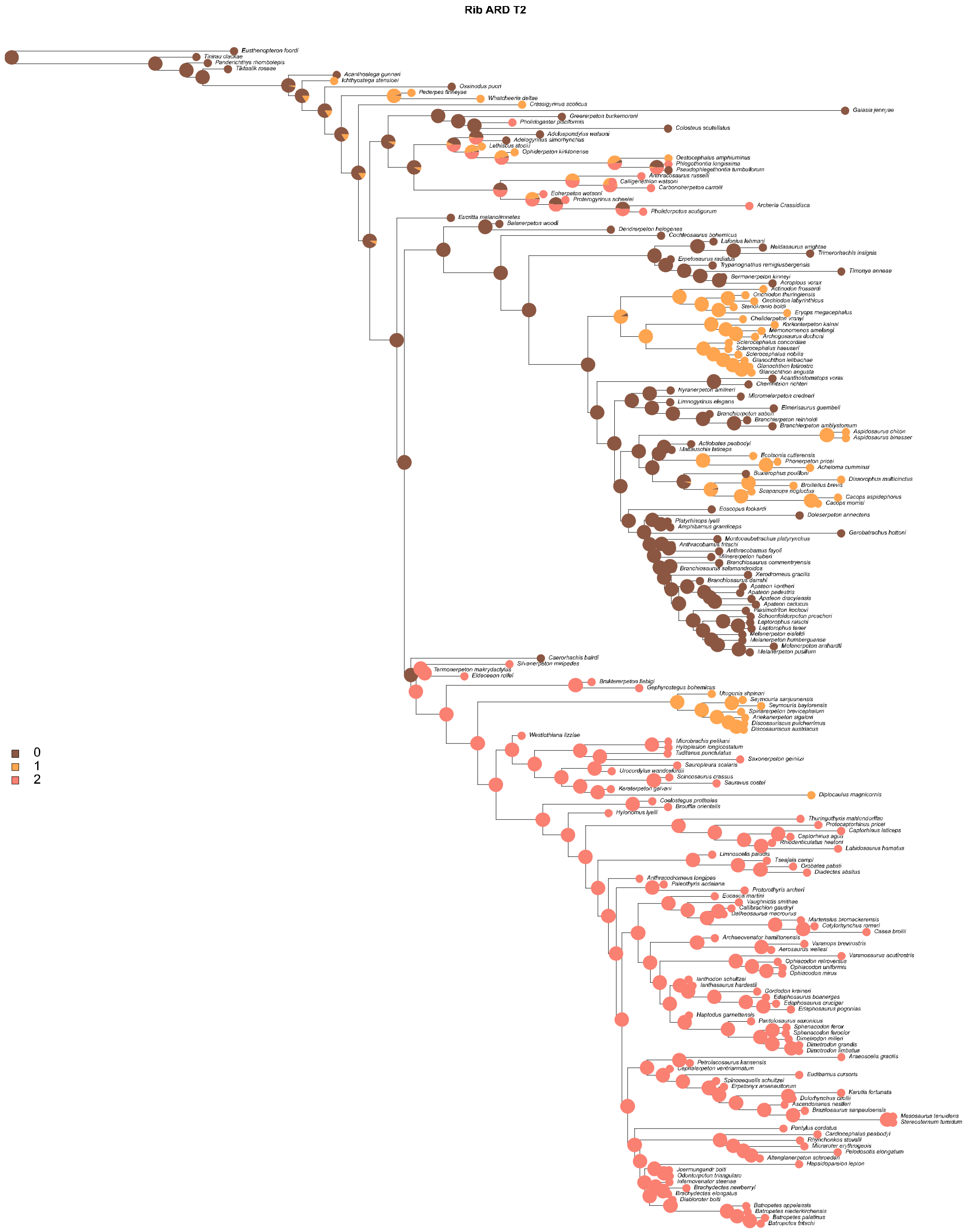

Fig. S59. Summarizing of trunk rib morphology ancestral state reconstruction, using ARD model and dated trees constrained as **topology 2**.

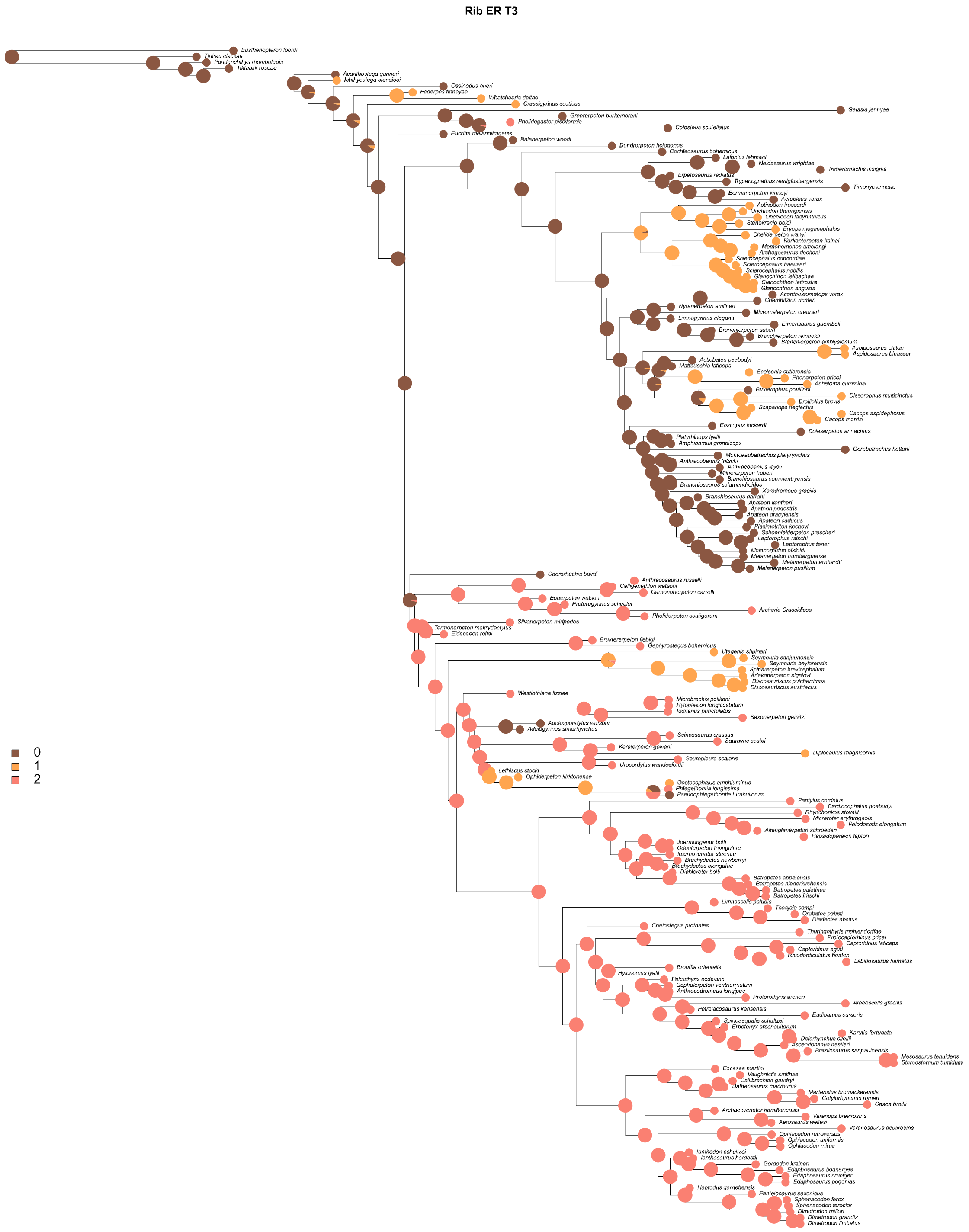

Fig. S60. Summarizing of trunk rib morphology ancestral state reconstruction, using ER model and dated trees constrained as **topology 3**.

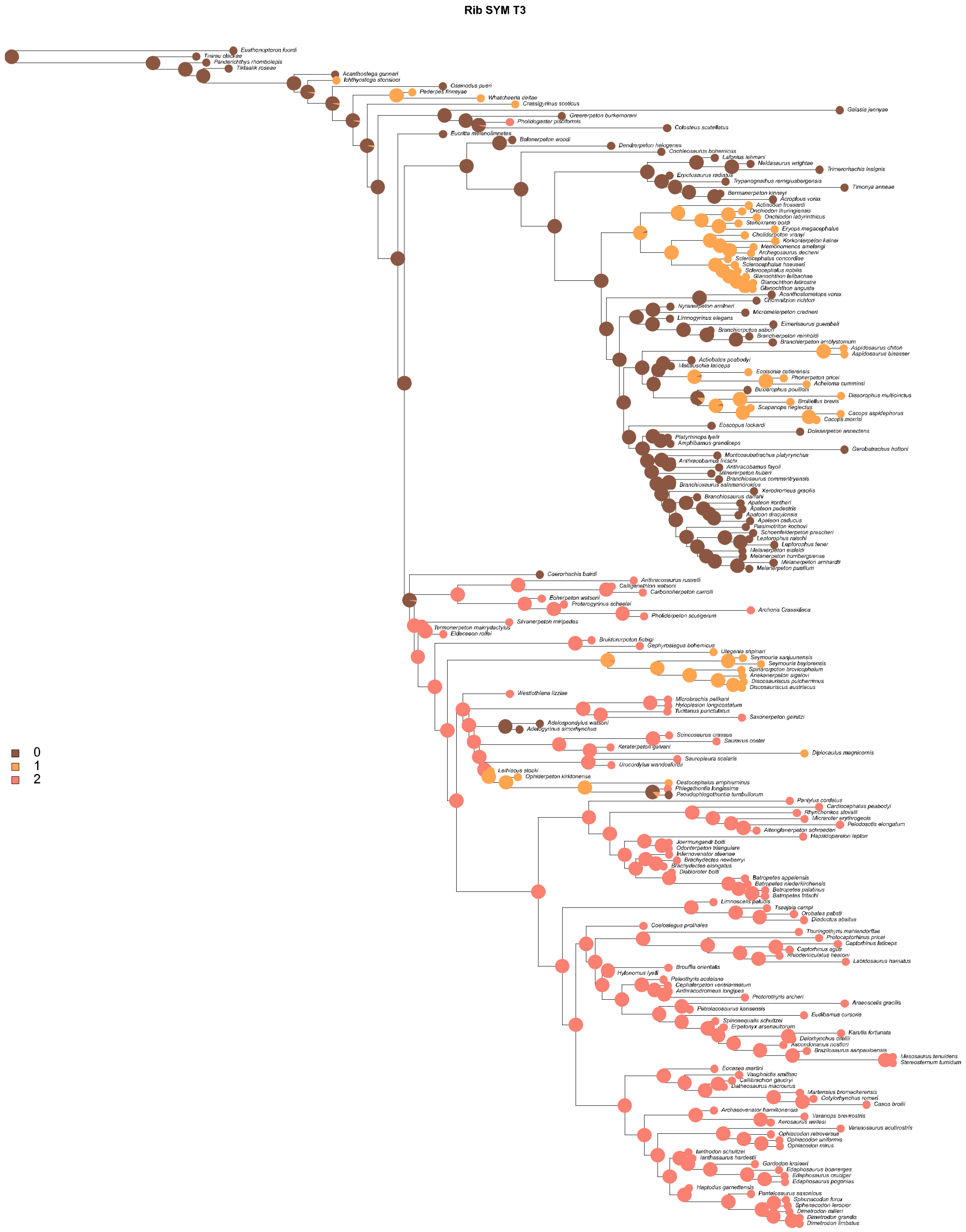

Fig. S61. Summarizing of trunk rib morphology ancestral state reconstruction, using SYM model and dated trees constrained as **topology 3**.

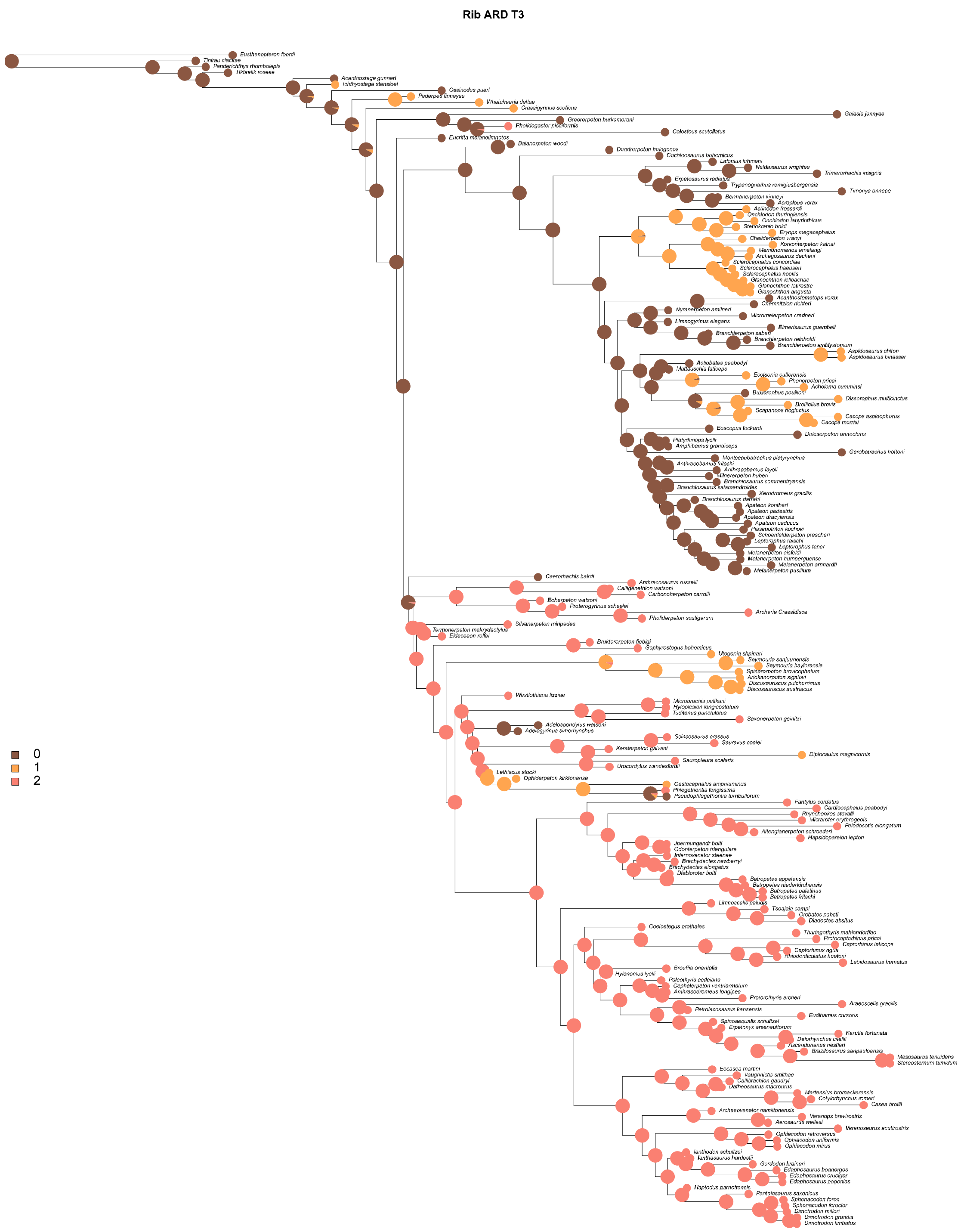

Fig. S62. Summarizing of trunk rib morphology ancestral state reconstruction, using ARD model and dated trees constrained as **topology 3**.

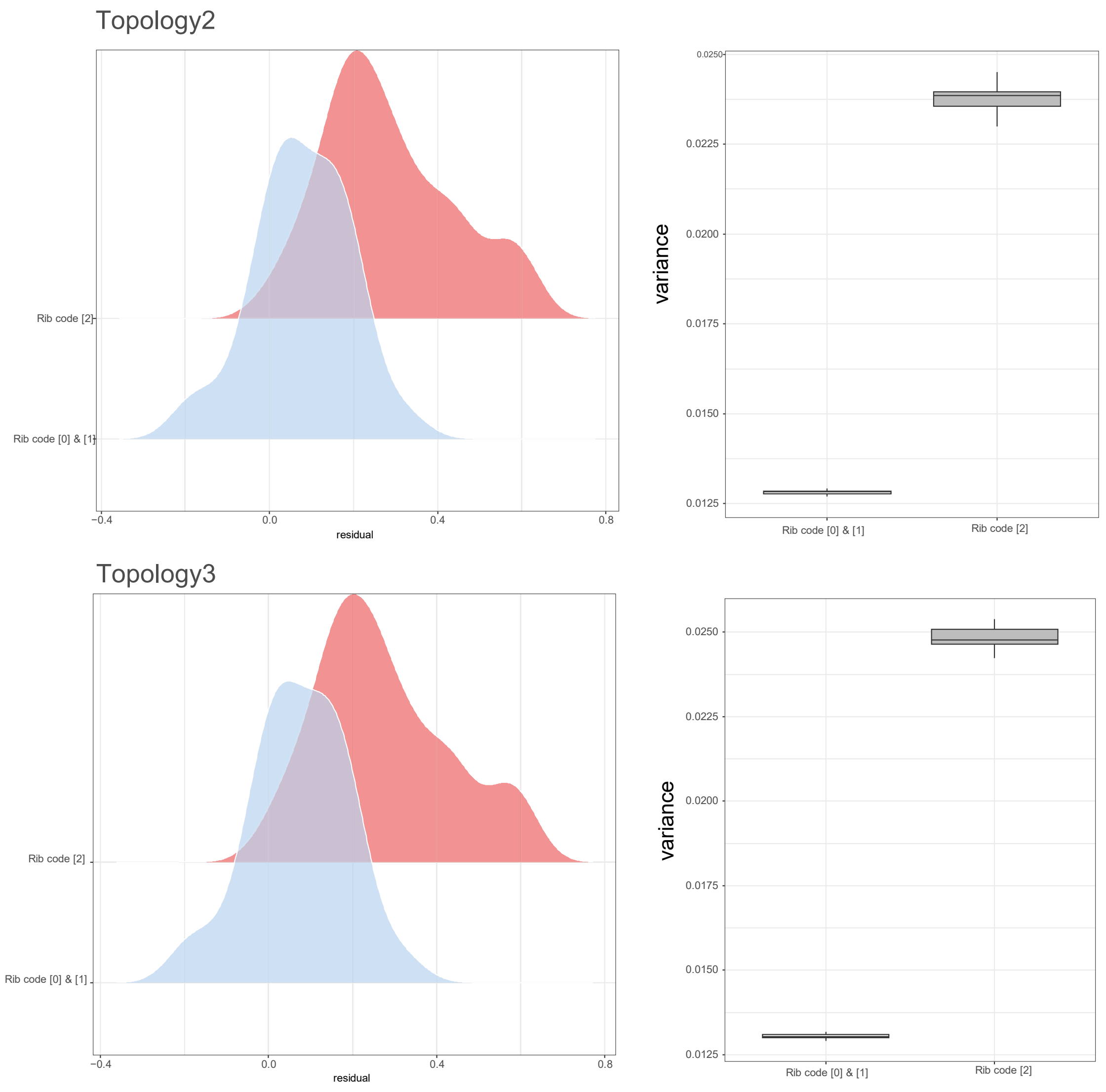

Fig. S63. Relative skull height density plot and variance boxplot of regimes defined by rib morphology.

Topology1

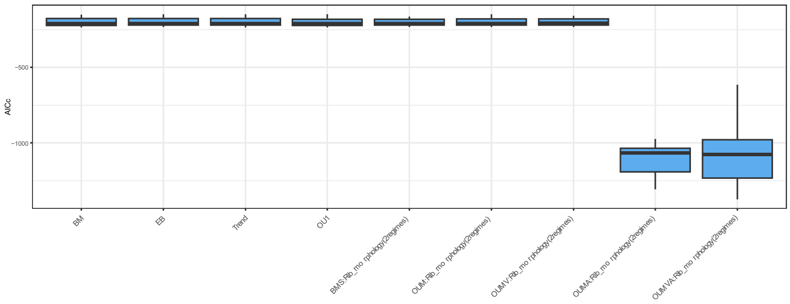

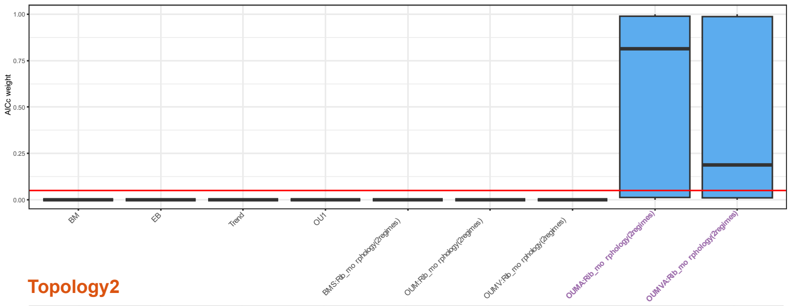

Topology2

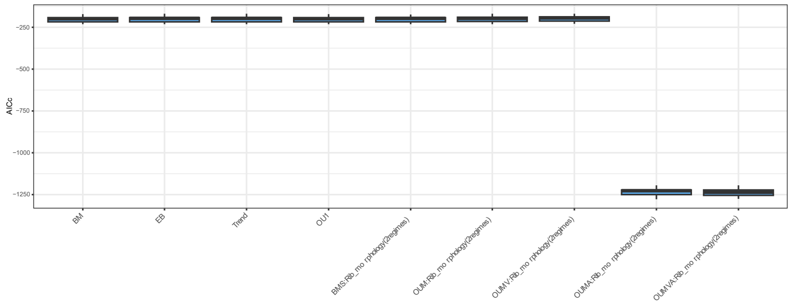

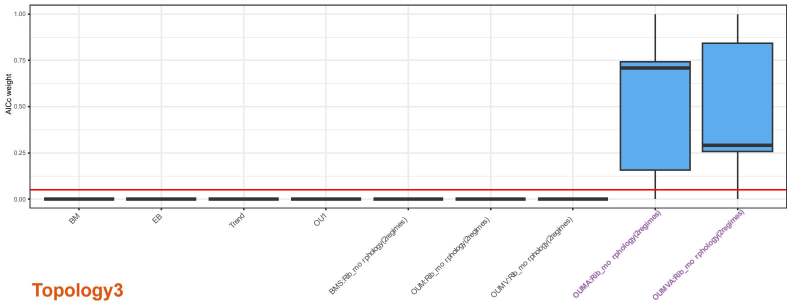

Topology3

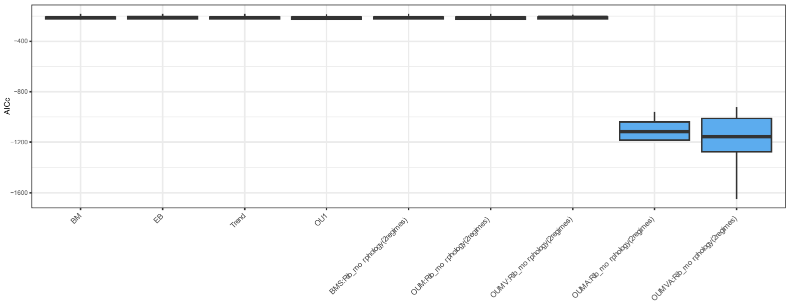

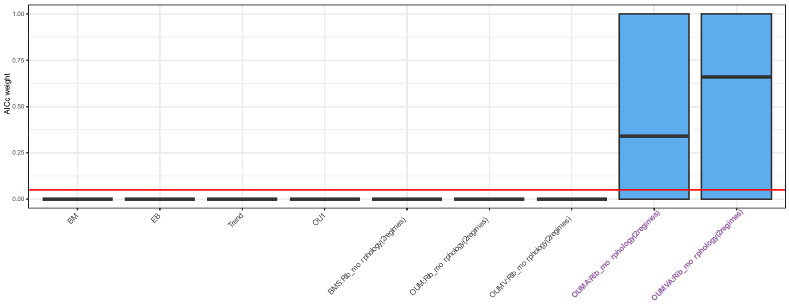

Fig. S64. AICc weight and AICc value from uniform models and 2-regime models with regimes defined by rib morphology, fitted with relative skull height and dated trees constraint as **topology 1, 2 and 3**.

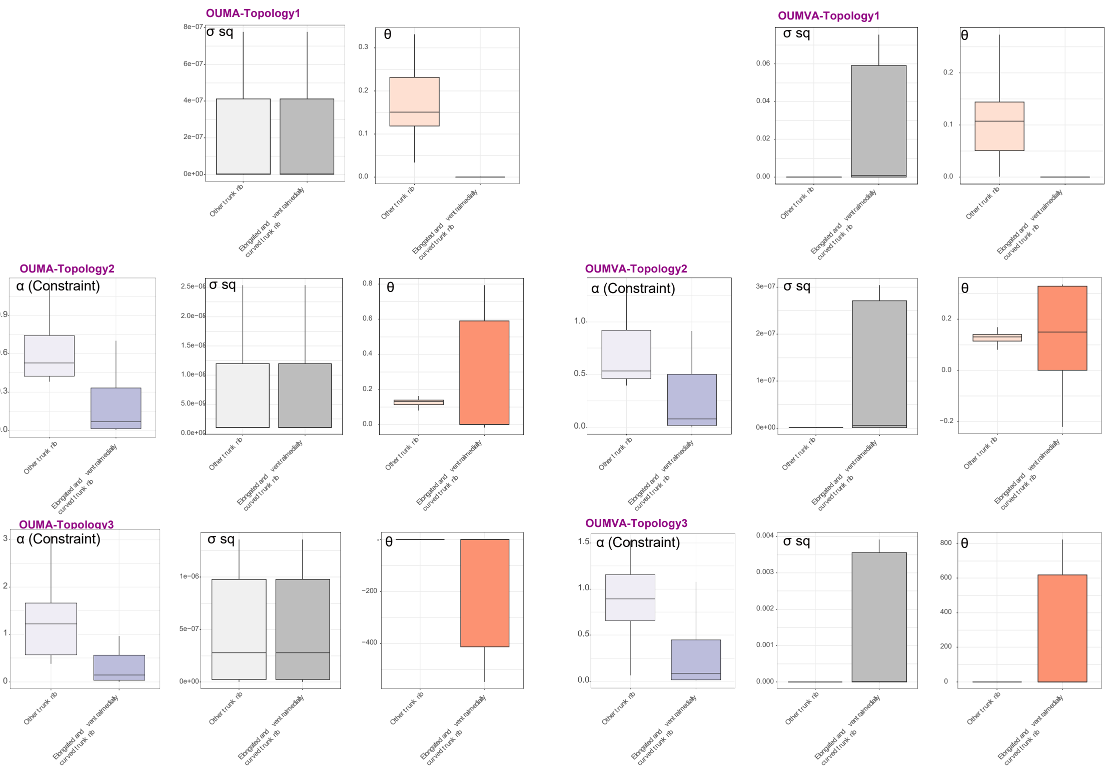

Fig. S65. Parameters estimated from non-negligible models, fitted with relative skull height, using dated trees constrained as **topology 1, 2 and 3**. Regimes are defined by rib morphology. For constraint(α), see main text figure4.

Caption for Data S1: This spreadsheet includes all data used in the current study, and descriptions of how the data were collected is given for each species. References for data collection are given in the same spreadsheet. Abbreviations : SL: midline skull length; OSL: postorbital skull length; SW: skull width; SH: skull height. Measurements are in millimetres. Cervical code: (0) No cervical vertebrae; (1) 1-2 cervical vertebrae; (2) 3-4 cervical vertebrae; (3) 5 or more cervical vertebrae. Trunk rib code: (0) extreme short rib, which is shorter than or approximately equal to twice of the transverse width of a centrum; (1) elongated ribs with relatively straight shaft, sometimes bearing extensive flanges or uncinate processes that create broad intercostal overlap; (2) mesiodistally curved and elongated ribs, with no or weak flanges.
